# Therapeutic HIV-1 Tat vaccination promotes durable immune reconstitution and reservoir reduction in ART-treated adults with clade C infection: a 12-year follow-up study

**DOI:** 10.3389/fimmu.2026.1769223

**Published:** 2026-04-10

**Authors:** Orietta Picconi, Cecilia Sgadari, Antonella Tripiciano, Sonia Moretti, Maria Teresa Maggiorella, Vittorio Francavilla, Anna Casabianca, Chiara Orlandi, Roberto Belli, Clelia Palladino, Massimo Campagna, Flavia Mancini, Leonardo Sernicola, Maria Rosaria Pavone Cossut, Fulvia Pimpinelli, Mauro Magnani, Fabrizio Ensoli, Matsontso Mathebula, Aurelio Cafaro, Paolo Monini, Maphoshane Nchabeleng, Barbara Ensoli

**Affiliations:** 1 National HIV/AIDS Research Center, Istituto Superiore di Sanità, Rome, Italy; 2 Department of Biomolecular Science, University of Urbino, Urbino, Italy; 3 Laboratory of Microbiology and Virology, San Gallicano Dermatological Institute, IRCCS, Rome, Italy; 4 Laboratory of Clinical Pathology and Microbiology, San Gallicano Dermatological Institute, IRCCS, Rome, Italy; 5 Mecru Clinical Research Unit (MeCRU), Sefako Makgatho Health Sciences University (SMU), Gauteng, South Africa; 6 National Health Service, Dr George Mukhari Microbiology Laboratory, Pretoria, South Africa

**Keywords:** aids, ART, CD4+ T cells, clinical trials, gender, HIV, proviral DNA, Tat

## Abstract

**Introduction:**

Antiretroviral therapy (ART) suppresses HIV replication but fails to eradicate viral reservoirs or fully restore immune competence. Therapeutic vaccination targeting HIV-1 Tat, a key viral protein persistently expressed also during ART, may enhance immune reconstitution and limit reservoir maintenance. Long-term data in individuals infected with HIV-1 clade C, particularly in sub-Saharan Africa, are lacking.

**Methods:**

We conducted a 12-year extended follow-up of 161 ART-treated adults previously enrolled in a randomized, placebo-controlled phase 2 trial of therapeutic HIV-1 Tat vaccination in South Africa (ISS T-003; SANCTR n. DOH-27-0211-3351; ClinicalTrials.gov: NCT01513135). No booster immunizations were administered. Longitudinal outcomes included anti-Tat antibody (Ab) persistence, CD4^+^ T-cell counts, plasma HIV RNA, and total HIV DNA in peripheral CD4^+^ T cells. Analyses were stratified by sex and ART adherence.

**Results:**

Ninety-seven percent of vaccinees developed anti-Tat Abs after immunization, and 70% were still positive at the beginning of the extended follow-up (3 years). Among these, 39% maintained Ab responses for their entire observation period (median duration 4 years), and 17 participants (20%) remained anti-Tat Ab seropositive up to year 12. Moreover, both Ab durability and peak titers were greater in vaccinees than in naturally occurring antibodies in seroconverter placebos. Compared with placebo, Tat vaccination was associated with earlier and sustained CD4^+^ T-cell recovery and accelerated decline of total HIV DNA, effects that persisted for more than a decade. These benefits were most pronounced in men, who were more immunocompromised at baseline, and in individuals with suboptimal ART adherence. Notably, vaccinated participants with intermittent viremia maintained stable or increasing CD4^+^ T-cell counts and continued reservoir reduction over time.

**Discussion:**

Therapeutic HIV-1 Tat vaccination induces long-lasting immunological benefits that extend beyond viral suppression achieved by ART alone, promoting durable immune reconstitution and progressive reservoir decay in clade C infection. These findings confirm results from a long-term follow-up of a parallel phase 2 trial in Italy, and both support Tat vaccination as a potential ART-intensifying strategy, particularly in populations with advanced immunosuppression or imperfect ART adherence.

**Trial registration:**

SANCTR n. DOH-27-0615–4948 and ClinicalTrials.gov: NCT02712489; and SANCTR n. DOH-27-072022–7347 and ClinicalTrials.gov: NCT05680948.

## Introduction

Antiretroviral therapy (ART) has substantially improved both life expectancy and quality of life among people with HIV (PWH). Nevertheless, ART does not fully restore immune competence, particularly in individuals who initiate treatment late, with severely depleted CD4^+^ T-cell counts, nor does it eradicate latent viral reservoirs, necessitating lifelong treatment to prevent viral rebound and disease progression (1). Moreover, HIV gene products continue to be expressed during ART (2), sustaining chronic immune activation and dysregulation that contribute to higher comorbidity and mortality rates compared with the general population (3). Suboptimal adherence to ART, often driven by drug-related side effects, further undermines treatment outcomes.

These limitations underscore the need for novel therapeutic strategies to complement ART, especially in sub-Saharan Africa, which accounts for more than 60% of the global PWH population (4). Long-acting ART formulations may help address adherence challenges; however, their high cost, restricted availability across the continent, and recent reductions in HIV/AIDS funding limit their widespread implementation (4). Therapeutic HIV vaccines used in combination with ART could provide a cost-effective and sustainable means to enhance ART efficacy by accelerating and strengthening virological and immunological recovery, while mitigating the negative effects of suboptimal ART adherence.

Despite promising safety and immunogenicity profiles, most therapeutic HIV vaccines have yielded limited clinical benefit (5, 6). To date, no therapeutic vaccine candidates have been evaluated in the context of C-clade HIV infection in sub-Saharan Africa, and studies exploring potential sex-based differences in vaccine response are very limited, even though men in this region experience poorer HIV outcomes than women, characterized by lower testing rates, delayed diagnosis, poorer ART adherence, and higher mortality (7).

Therapeutic vaccination with the HIV-1 Tat protein has shown promise in overcoming these challenges. Tat is a key viral regulatory protein that governs HIV gene expression, replication, and transmission, and contributes to disease progression and associated comorbidities (6). During ART, Tat continues to be expressed and released extracellularly, counteracting both virological and immunological recovery (6, 8, 9). Further, extracellular Tat, which is present on virions (10, 11), binds the envelope (Env) spikes forming a virus entry complex that favors infection of dendritic cells and T cells, key components of the virus reservoir (12). Of note, by binding the Env C–C chemokine receptor 5 (CCR5) co-receptor binding sites, Tat shields Env from anti-HIV antibodies (Abs), thus inhibiting virus neutralization, which, however, is restored by anti-Tat Abs (12). Notably, anti-Tat Abs are uncommon in natural infection and, when present, correlate with the asymptomatic state, higher CD4^+^ T-cell number, lower viral load, and reduced disease progression (13–17). This suggested that the induction of effective anti-Tat Abs and immunity may represent a pathogenesis-driven intervention to block progression and to intensify cART efficacy. Indeed, in phase 1 (18, 19) and phase 2 clinical trials conducted in Italy (ISS T-001 and ISS T-002, respectively), Tat vaccination induced durable increases in CD4^+^ T-cell counts, reduced immune activation and dysregulation, and led to a significant and progressive decline in HIV proviral DNA that persisted for up to eight years of follow-up (ISS T-002 EFUP) in adults infected with B-clade HIV (8, 9, 20). Of note, these effects occurred in PWH in chronic ART (mean years ± SD, 6 ± 5 at trial start and 10 ± 5 at EFUP start, respectively).

In parallel, Tat immunization was evaluated in 200 ART-treated, virologically suppressed South African adults infected with C-clade HIV, enrolled in a 48-week, randomized, placebo-controlled, double-blind phase 2 trial (ISS T-003) (21). Vaccination elicited high titers of cross-clade neutralizing anti-Tat Abs and increased CD4^+^ T-cell counts, particularly among participants with persistently low baseline levels despite long-term ART (mean years ± SD, 3 ± 2 at trial start). The improvement correlated with the magnitude of anti-Tat Ab responses. Notably, in non-compliant individuals (n=24), Tat vaccination prevented viral rebound and maintained CD4^+^ T-cell counts above baseline compared with placebo (21).

Here, we report findings from a 12-year extended follow-up study (ISS T-003 EFUP) involving 161 participants from the original ISS T-003 trial in South Africa (ART mean years ± SD: 5 ± 3 since trial start). This study evaluated the long-term persistence of ART intensification in the absence of booster vaccination within the C-clade epidemic setting, where a therapeutic vaccine is most urgently needed. We also investigated the influence of sex and ART adherence. Our results demonstrate that Tat vaccination exerts durable immunological and virological effects, sustaining CD4^+^ T-cell increases and continued proviral DNA decline, particularly among men and individuals with poor ART adherence.

## Materials and methods

### Study description

**Figure 1:**
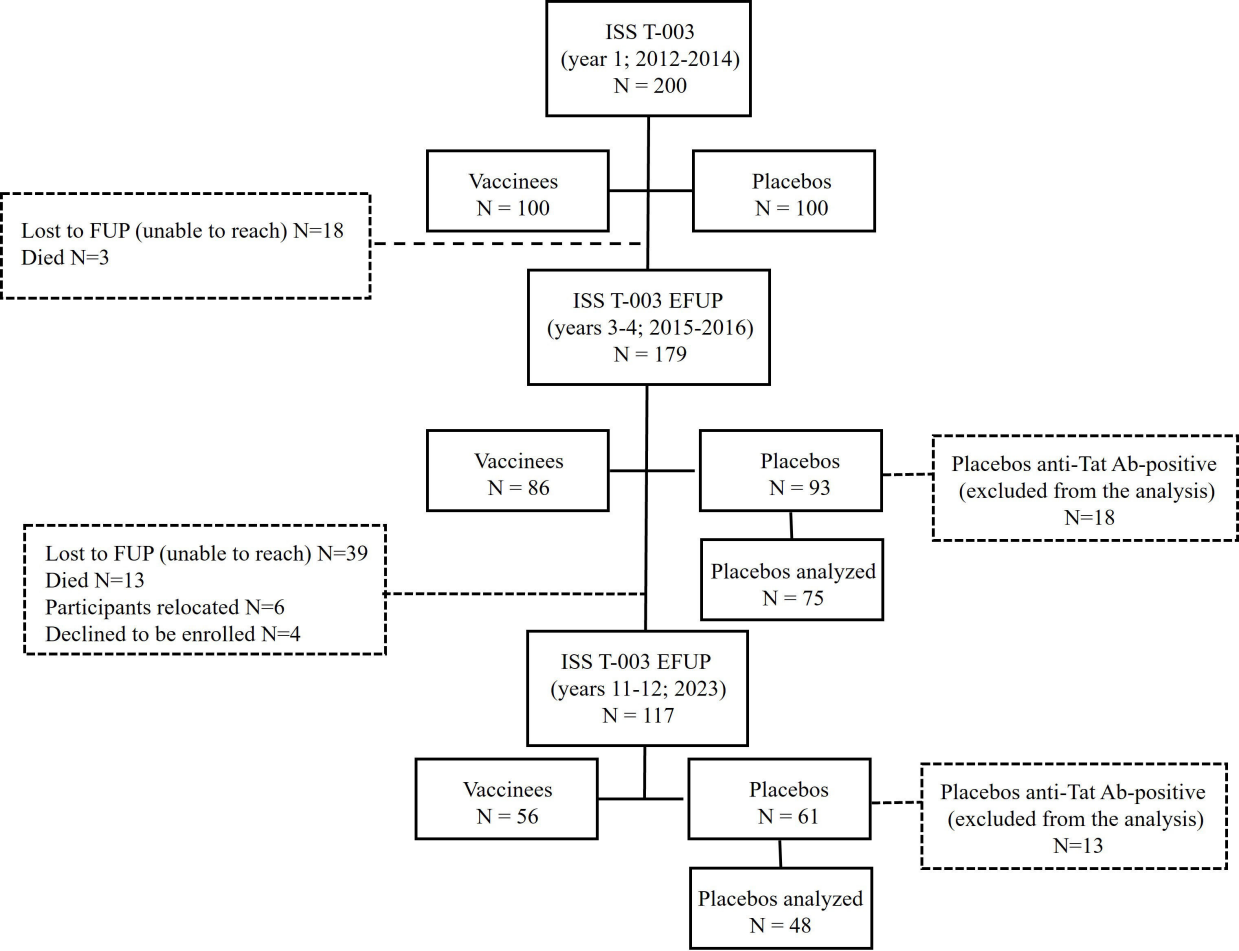
CONSORT flow diagram. The number of participants enrolled, followed-up from year 1 to year 12, and analysed is shown for the vaccine and placebo groups, respectively. Reasons for drop-out during the different phases are also reported. Before year 2015 ART was changed to a fixed dose combination (FDC) (tenofovir, lamivudine, EFV) in compliance with the 2013 South African guidelines. Before year 2023 participants were switched to a dolutegravir-based FDC, in compliance with the 2019 national guidelines.

This manuscript reports data from 2 extended follow-up studies of the 48-week randomized, placebo-controlled, double-blind ISS T-003 therapeutic trial registered prospectively in the South African National Clinical Trials Registry (SANCTR) (https://sanctr.samrc.ac.za/) as SANCTR n. DOH-27-0211-3351, as well as on ClinicalTrials.gov as NCT01513135. The 2 follow-up studies reported here were also registered prospectively as SANCTR n. DOH-27-0615-4948 (ISS T-003 EF-UP, started on January 2015) and SANCTR n. DOH-27-072022-7347 (ISS T-003 EF-UP2020, started on January 2023), as well as in ClinicalTrials.gov as NCT02712489 and NCT05680948, respectively. Of note, the registration in ClinicalTrials.gov for the NCT02712489 study was retrospective, since it was given priority to the South African registry to inform the local stakeholders about the study. The authors acknowledge the importance of prospective registration not only in the country where the study is conducted, but also in a Primary Registry before enrolment begins (**Figure 1**). In the ISS T-003 trial, 200 volunteers were immunized intradermally with the biologically active B-clade Tat at 30 μg doses given 3x or placebo (1:1 randomization) (21). One hundred seventy-nine trial participants agreed to enter this follow-up study after 2 years (range 2 to 3 years) up to 12 years from the first immunization (**Figure 1**). Eighteen placebo recipients that seroconverted for anti-Tat Abs during the 48 weeks following immunization (21) were compared with vaccine recipients only with respect to anti-Tat Ab responses. Anti-Tat Ab seroconvertion can occur also in ART (8, 17, 22) since ART does not block Tat gene expression.

A total of 161 volunteers (86 vaccinees and 75 placebos) were thus evaluated, with a median follow-up of 540 weeks (range 120–612 weeks) (**Figure 1**). The study aim was to evaluate the persistence of ART intensification by the Tat vaccine. The primary endpoint was measurement of anti-Tat Abs, while evaluations of CD4^+^ T-cells, viral load and proviral HIV DNA were the secondary endpoints. Full details about study design can be found in study protocols (Appendix 1) and are described elsewhere (21).

The study was conducted at the Mecru Clinical Research Unit (MeCRU), Sefako Makgatho Health Sciences University (SMU), Gauteng Province, South Africa. Follow-up visits were conducted from January 2015 to April 2016 (3 visits, 6-months apart), and from January to December 2023 (2 visits, 6-months apart). Volunteers received ART according to current National guidelines at the Health Facilities of the MeCRU catchment area (Tshwane District 1) throughout the trial. Specifically, during year 1 almost all volunteers (97%) were on an efavirenz (EFV) or nevirapine (NVP)-based regimen, which was changed to a fixed dose combination (FDC) (tenofovir, lamivudine, EFV) between year 1 and 3 of the follow-up in compliance with the 2013 South African guidelines, in order to replace the multi-pill regimen and improve compliance and retention in care. Further, between year 4 and year 11 of follow-up, participants were switched to a dolutegravir-based FDC, due to its greater efficacy on viral suppression and CD4^+^ T cell increase in compliance with the 2019 national guidelines.

All subjects signed an informed consent prior to enrolment. The study was conducted in accordance with the current Declaration of Helsinki and International Conference on Harmonization Good Clinical Practice guidelines and was approved by the SMU Research and Ethics Committee (SMUREC), the Tshwane Research and Ethics Committee, and the South Africa Medicines Control Council.

CD4^+^ T-cell levels were measured at the National Health Laboratory Service/Dr. George Mukhari (DGM) Laboratory, according to standard national laboratory measurements. HIV plasma viral load was determined at DGM Laboratory by the Abbott Real Time HIV-1 assay (lower limit of detection 40 RNA copies/mL) (21), for the first follow-up visits, and at the Microbiology and Immunology Laboratory, San Gallicano Dermatological Institute, IRCCS, Rome, Italy, by Aptima HIV-1 Quant Dx assay (lower limit of detection 30 RNA copies/mL), for the second follow-up period.

### Measurement of serum anti-Tat antibodies

Serum IgM, IgA and IgG against B-clade Tat were assessed by enzyme-linked immunosorbent assay (ELISA) and titers calculated as previously described (20, 21). Detection limit of Ab reciprocal titers were ≥25 for IgM and IgA; ≥100 for IgG.

### Quantification of HIV-1 DNA by real-time PCR

Total HIV-1 DNA quantification was performed by SYBR green real-time polymerase chain reaction (qPCR), as described (20). Cellular DNA was obtained from 1.6 mL of peripheral blood. After incubation for 45 min at 37 °C in a lysis buffer (sodium dodecyl sulfate 5%, 8M Urea, 0.3M NaCl, 10 mM Tris-HCl pH 7.5, 10 mM EDTA pH 8.0), DNA was purified by phenol extraction followed by ethanol precipitation and RNase treatment or by silica-gel membrane columns (QIAamp DNA blood mini kit, Qiagen), and concentrations determined by a NanoVue Plus Spectrophotometer (GE Healthcare).

Real-time PCR was performed as already described (20, 21) on samples analyzed at least in triplicate. HIV-1 DNA copy number was estimated as described (23). Results were expressed as log_10_ copies/10^6^ CD4^+^ T cells, calculated as the ratio between copies/μg DNA and the CD4^+^ T-cell number present in 1.5× 10^5^ white blood cells (WBC) using the following formula: [(copies/μg DNA)/(CD4^+^ T-cell number/WBC) × 150,000 WBC] × 10^6^ (20).

### Statistical analyses

Descriptive statistics summarizing quantitative variables included mean and standard deviation, or median and minimum and maximum; qualitative variables were presented as number and percentage. Chi-Square test or Fisher Exact Test were used to compare proportion between groups, while Student’s t-test was used to evaluate statistical differences in quantitative variables at baseline, due to their Gaussian distribution.

The Kaplan-Meier method was used to assess the cumulative probability of anti-Tat Ab persistence (at least 1 isotype) in vaccinees and placebos who developed anti-Tat Abs during the 48 weeks after immunization, and compared by the log-rank test. Median peak of reciprocal anti-Tat Ab titers were compared between vaccinees and anti-Tat Ab seroconverter placebos by the Wilcoxon–Mann–Whitney test. Longitudinal analysis for repeated measurements (including multiple measurements per year) was used to evaluate changes from baseline of CD4^+^ T cells or HIV-1 DNA load at each year of follow up, both overall and upon stratification by sex, viremic status, and ART adherence to account for major clinical modifiers. Statistically significant differences between vaccinees and anti-Tat-negative placebo at each time point were assessed using multiple pairwise comparisons of group mean with Tukey’s *post hoc* test, which controls the family-wise type I error rate. In the same analyses, comparisons between the vaccine and placebo groups were also performed to evaluate the overall treatment-by-time effect.

Data distributions of individual changes from baseline of CD4^+^ T cells or HIV-1 DNA load for vaccinees and anti-Tat negative placebos were visualized using violin plots for descriptive purposes, with violin width representing kernel density estimation and overlaid points indicating individual observations.

Missing data were not imputed or otherwise manipulated; analyses were conducted on the available data only.

Statistical analyses were carried out at two-sided with a 0.05 significance level, using SAS^®^ (Version 9.4, SAS Institute Inc., Cary, NC, USA), GraphPad Prism (Version 10.6.1) and STATA™ version 8.2 (Stata Corporation, College Station, Texas, United).

## Results

### Study participants

A total of 200 participants (1:1 vaccinees *vs* placebos) were enrolled in the 48-weeks phase 2 trial in South Africa (21). Of those, 161 participants (86 vaccinees and 75 placebos) were then enrolled in the extended follow-up study [median of 540 weeks (range 120-612)]. All participants were black, except 1 placebo recipient (mixed race). The proportion of men was approximately twice as high in the vaccine group compared with the placebo group (33% *vs* 19%; p=0.0452) (**Table 1a**). No significant differences were observed between groups in age, duration of HIV infection, ART duration or regimen, baseline CD4^+^ T-cell counts, or HIV DNA levels (**Table 1a**). Similarly, no baseline differences were detected between vaccinated and placebo men (**Supplementary Table 1a**) or women (**Supplementary Table 1b**).

**Table 1a:** Characteristics of study participants at the ISS T-003 trial baseline.

Characteristics	n	Tat.vaccine	n	Placebo	P.value
Race					
Black	86	100.00%	74	98.67%	
Mixed	0	0.00%	1	1.33%	
Gender					
Males	28	32.56%	14	18.67%	0.0452^+^
Females	58	67.44%	61	81.33%	
Age					
Mean ± SD	86	36 ± 6	75	35 ± 6	0.6518^++^
Years from HIV diagnosis					
Mean ± SD	86	5 ± 3	75	5 ± 3	0.8981^++^
Years from cART initiation					
Mean ± SD	86	4 ± 2	75	4 ± 2	0.8112^++^
cART regimen					
NNRTI or NRTI-based	84	97.67%	73	97.33%	0.8897^+^
PI-based	2	2.33%	2	2.67%	
CD4^+^(cells/μL)					
Mean± SD	86	522 ± 230	75	560 ± 175	0.2475^++^
HIV RNA (copies/mL)					
<40 (assay cut-off)	81	95.29%	75	100.00%	0.1232^+++^
≥40	4	4.71%	0	0.00%	.
HIV DNA (log_10_ copies/10^6^ CD4^+^)					
Mean ± SD	81	3.20 ± 0.44	74	3.14 ± 0.63	0.4891^++^

^+^ Chi Square Test; ^++^ T Student Test; ^+++^Fisher Exact Test; SD, Standard Deviation.

**Table 1b:** Characteristics of vaccinees by gender at the ISS T-003 trial baseline.

Characteristics	n	Males	n	Females	P value
Gender	28	32.56%	58	67.44%	
Age					
Mean± SD	28	37 ± 6	58	35 ± 6	0.0559^++^
Years from HIV diagnosis					
Mean± SD	28	4 ± 2	58	6 ± 3	0.0796^++^
Years from cART initiation					
Mean± SD	28	4 ± 2	58	4 ± 2	0.9129^++^
cART regimen					
NNRTI or NRTI-based	27	96.43%	57	98.28%	0.5477^+++^
PI-based	1	3.57%	1	1.72%	
CD4+ (cells/μL)					
Mean± SD	27	418 ± 140	58	572 ± 249	0.0038^++^
HIV RNA (copies/mL)					
<40 (assay cut-off)	26	92.86%	55	96.49%	0.5954^+++^
≥40	2	7.14%	2	3.51%	
HIV DNA (log_10_ copies/10^6^ CD4^+^)					
Mean± SD	25	3.40 ± 0.29	56	3.10 ± 0.47	0.0039^++^

^++^ T Student Test; ^+++^ Fisher Exact Test; SD, Standard Deviation.

**Table 1c:** Characteristics of placebos by gender at the ISS T-003 trial baseline.

Characteristics	n	Males	n	Females	P value
Gender	14	18.67%	61	81.33%	
Age					
Mean± SD	14	36 ± 7	61	35 ± 6	0.6860^++^
Years from HIV diagnosis					
Mean± SD	14	4 ± 2	61	5 ± 3	0.3380^++^
Years from cART initiation					
Mean± SD	14	3 ± 2	61	5 ± 3	0.0141^++^
cART regimen					
NNRTI or NRTI-based	14	100.00%	59	96.72%	1.0000^+++^
PI-based	0	0.00%	2	3.28%	
CD4+ (cells/μL)					
Mean± SD	14	485 ± 187	61	577 ± 169	0.1106^++^
HIV RNA (copies/mL)					
<40 (assay cut-off)	14	100.00%	61	100.00%	na
≥40	0	0.00%	0	0.00%	
HIV DNA (log_10_ copies/10^6^ CD4^+^)					
Mean± SD	14	3.21 ± 0.44	60	3.12 ± 0.67	0.5140^++^

^++^ T Student Test; ^+++^ Fisher Exact Test; na= not applicable; SD, Standard Deviation.

Within the vaccine group, men were slightly older than women (37 *vs* 35 years; p=0.0559), had a shorter duration of HIV infection (4 *vs* 6 years; p=0.0796), and exhibited significantly lower baseline CD4^+^ T-cell counts (418 *vs* 572 cells/μL; p=0.0038) and higher HIV DNA levels (3.40 *vs* 3.10 log_10_ copies/10^6^ CD4^+^ T cells; p=0.0039) (**Table 1b**). Among placebo participants, men had been on ART for a shorter duration than women (3 *vs* 5 years; p=0.0141) (**Table 1c**).

Longitudinal analyses of immunological and virological parameters were conducted from baseline throughout 12 years of follow-up. These also included stratification by sex and by the viremic status, and the between-arm difference between vaccinees and anti-Tat negative placebos both by year and overall.

### Long-term persistence of vaccine-induced anti-Tat antibodies as compared to naturally occurring anti-Tat Abs in seroconverted placebos

Anti-Tat Abs were induced in 97% of vaccinees during the phase-2 trial (21). These anti-Tat Abs (at least one isotype) persisted in 70% (58/83) of vaccinees, who resulted still positive at the enrolment in the extended follow-up study (year 3). Among these, 39% maintained Ab responses for their entire observation period (median duration 4 years), and 17 participants (20%) remained anti-Tat Ab seropositive until study end. Of the vaccinees with persistent responses, 12 maintained all three Ig isotypes, 16 retained two (5 IgM+IgG, 4 IgM+IgA, 7 IgG+IgA), and 30 retained one (13 IgM, 13 IgG, 4 IgA).

**Figure 2:**
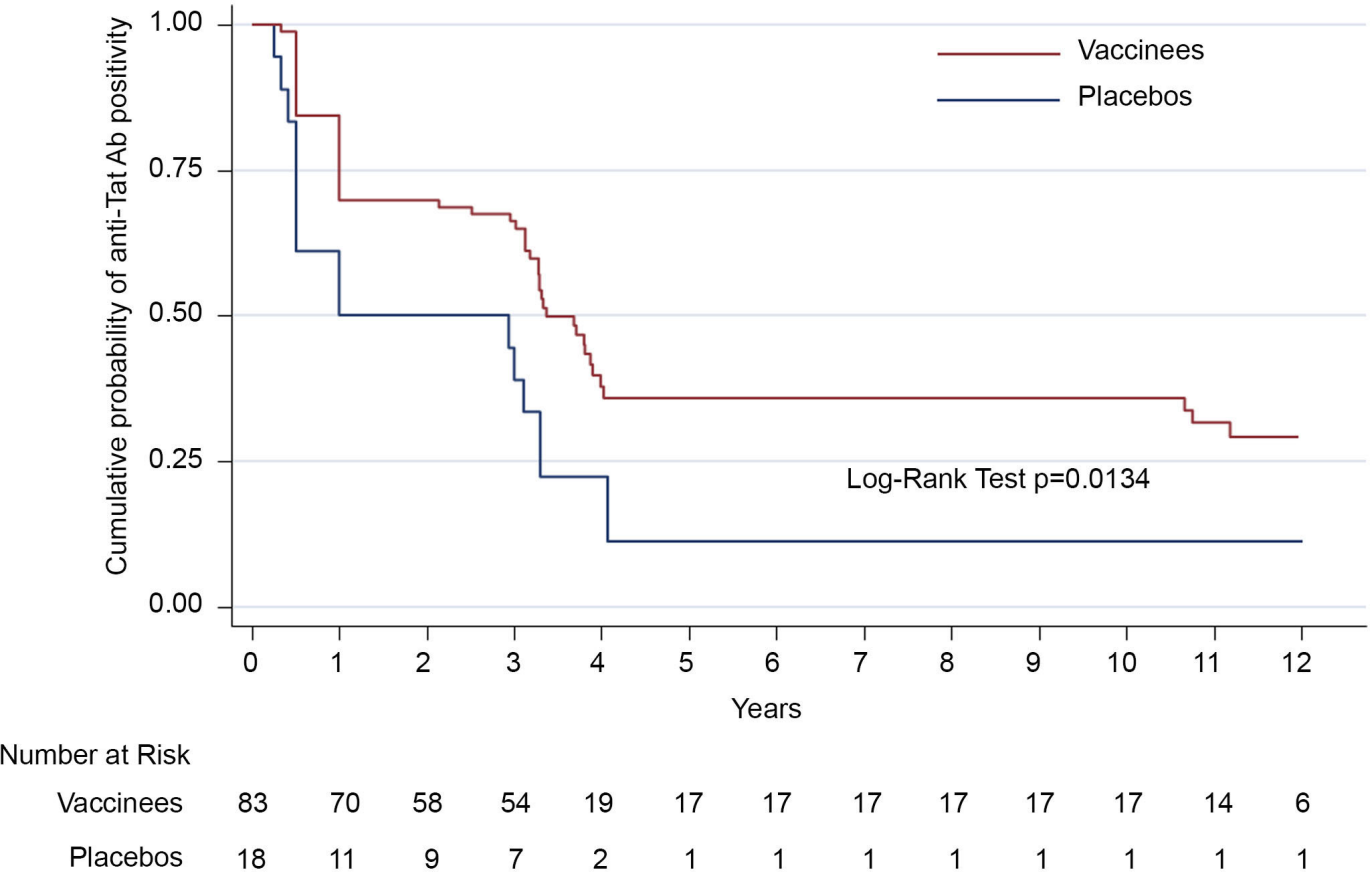
Anti-Tat Ab persistence in vaccinees and in anti-Tat Ab seroconverters placebos up to year 12 of follow up. Kaplan-Meier estimates showing the cumulative probability of anti-Tat Ab durability in vaccinees and anti-Tat Ab positive placebos up to 12 years of follow-up. Anti-Tat Abs persisted significantly longer in vaccinees as compared to the placebo group (p= 0.0134, log-rank test).

Among placebos, 18/100 (18%) seroconverted for anti-Tat Abs during the 48 weeks of the trial, an event already observed that may occur in around 30% in PWH also during ART (8, 17, 22). This gave us the opportunity to compare the vaccine-induced Abs with naturally occurring anti-Tat Abs. As shown in **Figure 2**, anti-Tat Ab responses persisted significantly longer in vaccinees than in the 18 placebos who naturally developed anti-Tat Abs (log-rank test, *p* = 0.0134). Antibody persistence was also greater in vaccinees than in placebos seroconverters for both IgM and IgG isotypes (log-rank test: *p* = 0.0353 for IgM; *p* < 0.0001 for IgG, respectively), whereas a trend was observed for IgA (*p* = 0.0909) (**Supplementary Figure 1**).

Moreover, peak levels of individual Ig isotypes induced by Tat vaccination were higher than those naturally developed in placebos. In particular, a statistically significant difference was observed for IgG peak titers between vaccinees and placebos (median peak reciprocal titers 800 versus 100, respectively; p < 0.0001) (**Supplementary Table 2**). Further, median peak IgM titers were 38 (range, 25–400) in vaccinees and 38 (range, 25–100) in placebos, while median peak IgA titers were 50 (range, 25–1600) and 38 (range, 25–100), respectively in vaccinees and placebos (**Supplementary Table 2**). All further analyses excluded anti-Tat Ab seroconverter placebos.

### CD4+ T-cell dynamics in the total population and by sex

**Figure 3:**
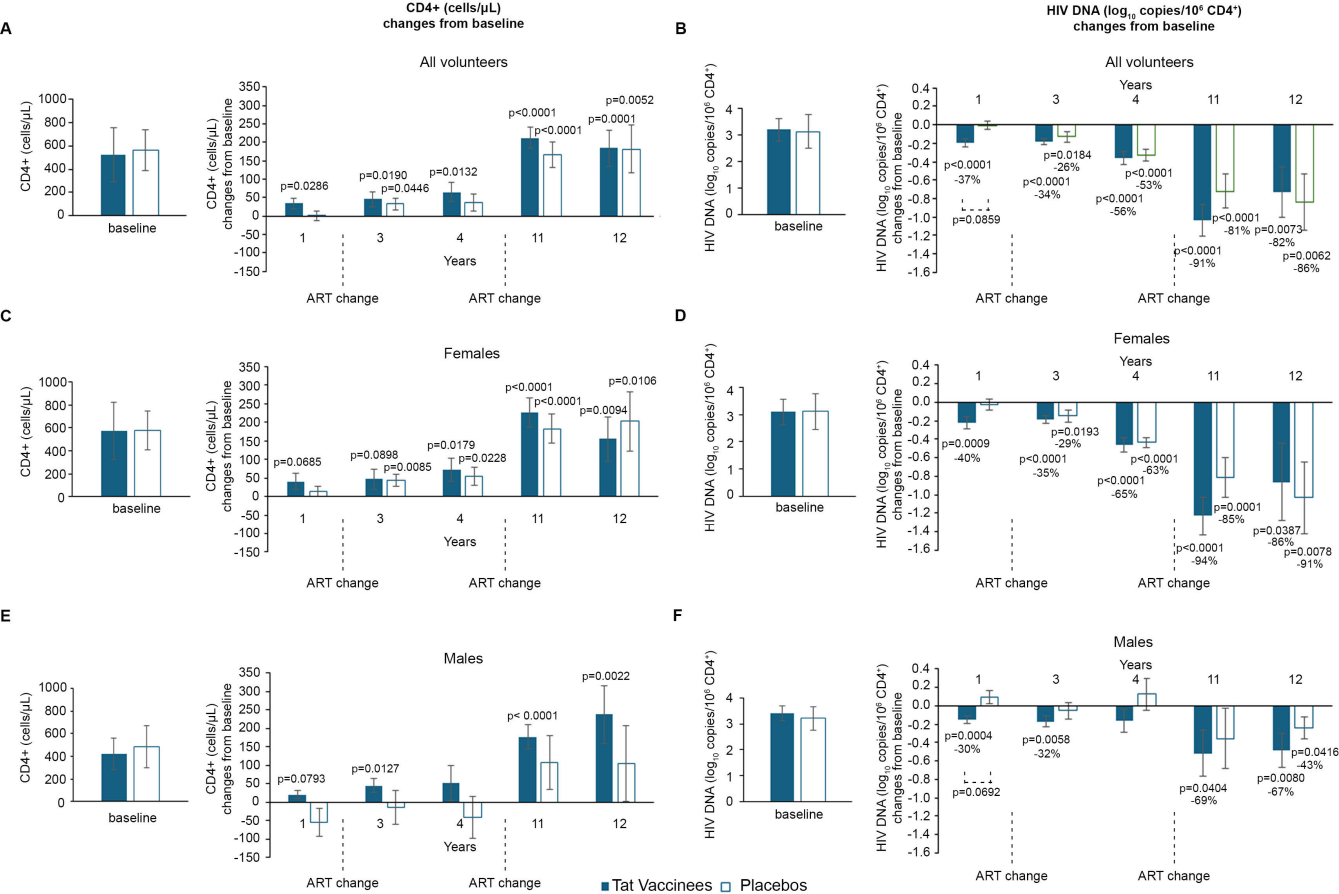
Changes from baseline of CD4^+^ T-cells and blood HIV DNA during 12 years of follow-up by treatment group and by gender. Baseline values (left panels) and annual changes (right panels) from ISS T-003 study entry of CD4+ T-cells in all volunteers (vaccinees and placebos) **(A)**, females **(C)**, and males **(E)**, and of HIV DNA levels (expressed as log10 copies/10^6^ CD4^+^ T-cells) in all vaccinees and placebos **(B)**, females **(D)**, and males **(F)** are shown, respectively. The number of participants is as follows: vaccinees n=86; placebos n=75; females vaccinated n=58; female placebos n=61; males vaccinated n=27; female placebos n=14. Data are presented as mean values with standard error. A longitudinal analysis for repeated measurements was applied and *post-hoc* pairwise comparisons were conducted using the Tukey’s test. P-values assess the values at year 1–12 *vs*. baseline ISS T-003 trial values.

In the overall population, vaccinees showed a progressive and sustained increase in CD4^+^ T-cell counts from baseline, reaching statistical significance from year 1 (+34 cells) and continuing to rise through years 11 (+212 cells) and 12 (+184 cells) (**Figure 3A**). Placebo participants showed CD4^+^ T-cell increases only at year 3 (+33 cells) following the first ART modification, and again at years 11 and 12 (+168 and +182 cells, respectively) after the second ART change. By year 12, CD4^+^ T-cell gains were comparable between groups.

Among women, CD4^+^ T-cell trends mirrored those of the total population. Female vaccinees exhibited increases from year 1 and 3 (+40 and +46 cells, respectively), reaching significance at year 4 and peaking at years 11 and 12 (+227 and +155 cells, respectively). Placebo females showed a delayed response, with significant increases at year 3 (+44 cells) and maximal gains at year 12 (+202 cells) (**Figure 3C**).

Male participants displayed distinct dynamics. Vaccinated men showed a progressive CD4^+^ T-cell increase reaching significance at year 3 (+45 cells) and continuing through year 12 (+237 cells). In contrast, placebo men experienced CD4^+^ T-cell declines up to year 4 (-55, -16, and -52 cells at years 1, 3, and 4, respectively) despite ART modification, with recovery only at years 11 and 12 (+107 and +105 cells) (**Figure 3E**). These results suggest that men, who started with lower CD4^+^ T-cell counts and higher proviral DNA levels than women, appeared to benefit most from Tat vaccination.

Between-arm differences (i.e., vaccinees *vs*. placebos) of CD4^+^ T-cell changes from baseline showed positive differences in vaccinees compared with placebo (i.e., an overall effect on CD4^+^ T cells in vaccinees, females, and males *vs*. placebo of +24.17, +24.17, and +86.07, respectively) (**Supplementary Table 3**). However, these differences were not statistically significant either overall or after stratification by gender, likely due to the reduced sample size and changes of drug regimens, except for viremic vaccinees (**Supplementary Table 3**).

The distribution of individual changes over time in CD4^+^ T-cell counts for individual subjects by violin plots confirmed that changes from baseline in CD4^+^ T-cell counts are more pronounced in vaccinees than placebos (**Supplementary Figure 3**).

### HIV proviral DNA in the total population and by sex

HIV proviral DNA in peripheral CD4^+^ T cells declined more rapidly in vaccine recipients than in placebo participants. Vaccinees exhibited a continuous and significant decline from year 1, reaching a 91% mean reduction (-1.04 log_10_ copies/10^6^ CD4^+^ T cells) by year 11. In contrast, placebo participants achieved a significant reduction only by year 3, reaching an 86% mean decline (-0.84 log_10_) at year 12 (**Figure 3B**).

Sex-stratified analysis revealed that female vaccinees achieved significant reductions from year 1, culminating in a 94% mean decrease (-1.23 log_10_) by year 11, whereas female placebos showed delayed declines beginning at year 3 (-1.03 log_10_, 91% mean reduction by year 12) (**Figure 3D**). In male vaccinees, proviral DNA declined from year 1, reaching a 69% mean reduction (-0.52 log_10_) by year 11, while placebo men showed stable or slightly increased proviral levels up to year 4, followed by modest reductions after the second ART switch (43% reduction; -0.24 log_10_ by year 12) (**Figure 3F**).

These data indicate that vaccinated men, despite being more immunocompromised at baseline, experienced the greatest relative benefit in terms of proviral DNA decline.

The between-arm differences of HIV proviral DNA changes from baseline at each year showed differences between vaccinees and placebos at year 1 both overall (-37% in vaccinees versus -0.7% in placebos) and in males (-30% in vaccinees versus +24% in placebos), although without reaching statistical significance (p=0.0859 and p=0.0692, respectively), while no statistically significant differences were observed between vaccinees and placebos (**Supplementary Table 3**). This also may be influenced by ART changes.

Lastly, violin plots showing the distribution of individual changes from baseline in HIV proviral DNA levels for vaccinees and placebos indicated that nearly all vaccinees had a decline in HIV proviral DNA, whereas some increases were observed in the placebo group (**Supplementary Figure 2**).

### Plasma viremia and ART adherence

Overall, 60-67% of participants maintained undetectable plasma HIV RNA (<40 copies/mL) throughout the follow-up, with no significant difference between vaccine and placebo groups. However, 58 participants (36%) experienced at least one episode of detectable viremia during the study. Within the first 48 weeks, 34 individuals (18 vaccinees, 16 placebos) showed viral load increases; 16 did not experience additional episodes of viral load increase, while 18 (13 vaccinees, 5 placebos) had additional episodes at years 3-4, and 5 (4 vaccinees, 1 placebo) again at years 11-12. After 48 weeks, 19 participants (10 vaccinees, 9 placebos) experienced their first detectable viral load between years 3-4, and 5 (1 vaccinee, 4 placebos) between years 11-12, likely indicating reduced adherence to ART. Notably, no vaccinees experienced a first viral rebound exclusively during the late follow-up period, whereas 5 placebo participants did and were excluded from subsequent analyses. The number of episodes of detectable viremia during the study is detailed in the **Supplementary Table 4** for both vaccinees and placebos, respectively.

**Figure 4:**
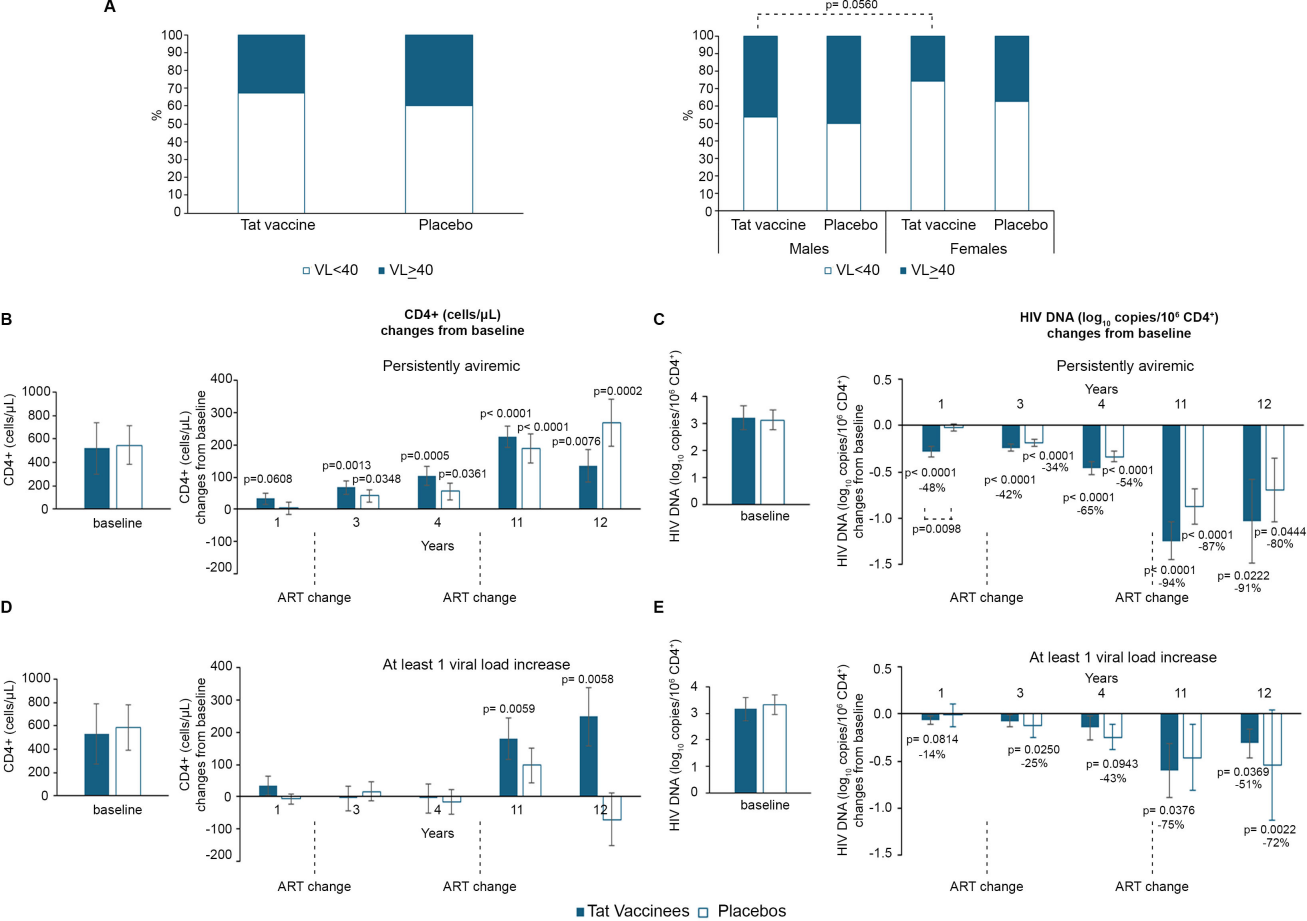
Viremia rebound, CD4^+^ T-cells and blood HIV DNA changes from ISS T-003 trial baseline by treatment group, over 12 years of follow up. Percentages of individuals that remained persistently aviremic (VL = <40 HIV-1 RNA copies/mL) or had at least 1 episode of viral load increase (VL > 40 HIV-1 RNA copies/mL) during the study **(A)** in vaccinees and placebos (left panel), and by gender (right panel). Baseline values (right panels) and annual changes from ISS T-003 study entry of CD4^+^ T-cells **(B)**, and HIV DNA levels (expressed as log_10_ copies/10^6^ CD4^+^ T-cells) **(C)** in vaccinees and placebos that remained persistently aviremic and of CD4^+^ T-cells **(D)**, and HIV DNA levels (expressed as log_10_ copies/10^6^ CD4^+^ T-cells) **(E)** in vaccinees and placebos with at least 1 episode of viral blip up to year 4, are shown. The number of participants tested is: aviremic vaccinees n=58; placebos n=45; viremic vaccinees n=28; placebos n=25. In **(A)** data are presented as percentage and the p-values refers to the Chi-square test. From **(B)** to **(E)** data are presented as mean values with standard error and a longitudinal analysis for repeated measurements was applied and *post-hoc* pairwise comparisons were conducted using the Tukey’s test. P-values assess the values at year 1–12 *vs*. baseline ISS T-003 trial values.

Sex-stratified analysis showed that approximately 50% of men, regardless of treatment group, had at least one viremic episode, compared with 26% of vaccinated and 38% of women placebo (p=0.06), respectively (**Figure 4A**).

### CD4^+^ T-cell and HIV DNA dynamics by viremic status

Among aviremic participants, Tat vaccination was associated with earlier and stronger CD4^+^ T-cell recovery and proviral DNA decline. In aviremic vaccinees (n=58), CD4^+^ T-cell counts increased from year 1 (+33 cells), reaching significance at year 3 (+68 cells) and peaking at years 11 (+226) and 12 (+133). Aviremic placebos (n=45) showed significant increases only at year 3 (+41 cells), with maximal gains at year 12 (+267) (**Figure 4B**).

Similarly, proviral DNA declined continuously in aviremic vaccinees (94% mean reduction; -1.25 log_10_ by year 11), whereas in placebos, reductions were modest at year 1 (5% *vs* 48%; p=0.0098), becoming significant only at year 3 and reaching 87% (-0.87 log_10_) by year 11 (**Figure 4C**).

Among participants with at least one viremic episode, CD4^+^ T-cell counts remained stable in both groups until year 4. Subsequently, viremic vaccinees (n=28) exhibited significant CD4^+^ T-cell increases at year 11 (+179 cells) and 12 (+248 cells), whereas no significant changes occurred in viremic placebos (n=25) (**Figure 4D**). Proviral DNA declined significantly in viremic vaccinees at years 11 (75% mean reduction; -0.60 log_10_) and 12 (51%; -0.31 log_10_), while in placebos, reductions were observed at years 3 (25%; -0.12 log_10_) and 12 (71%; -0.55 log_10_) (**Figure 4E**).

Thus, aviremic vaccinees experienced earlier and persisting immunological and virological improvements, while those vaccinees with intermittent viremia showed stability of both parameters. The overall between-arm comparison showed that viremic vaccinees have increases of CD4^+^ T cells as compared to placebos (86 CD4^+^ T-cells more than the placebo group), although without reaching statistical significance (p = 0.0653), while aviremic vaccinees present a statistically significant decrease of proviral DNA as compared to placebos at year 1 (48% *vs* 5%, respectively, p = 0.0098) and a trend toward faster decline in vaccinees *vs*. placebos in the overall comparison (41% greater reduction compared to placebos, p = 0.0759) (**Supplementary Table 3**). In contrast, no statistically significant differences were observed in the between-arm comparison of changes from baseline in CD4^+^ T-cell counts between aviremic vaccinees and placebos.

### Mitigation of ART non-adherence by Tat vaccination

In the original ISS T-003 trial, non-compliant participants (n=24) who received the Tat vaccine exhibited containment of viral rebound and maintained CD4^+^ T-cell counts compared with placebo [21]. Drug-level monitoring confirmed poor adherence, with missed doses and treatment interruptions averaging 4 months (21).

**Figure 5:**
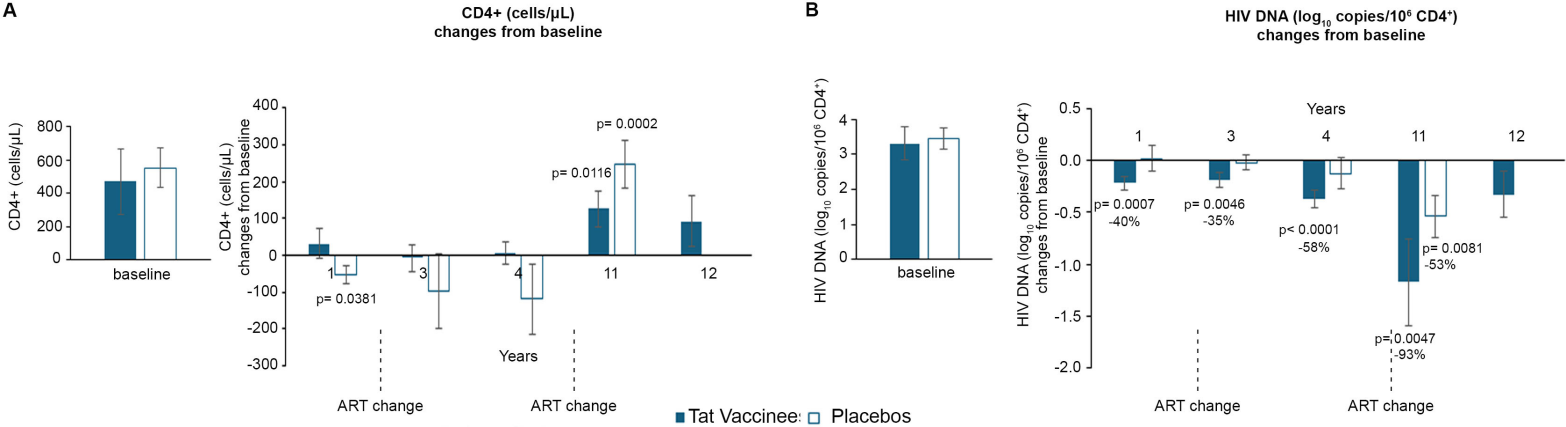
Long term follow-up of volunteers non-compliant to therapy during the first 48 weeks since the first immunization: CD4^+^ T-cells and blood HIV DNA changes from ISS T-003 trial baseline by treatment group. Baseline values (left panels) and annual changes (right panels) from ISS T-003 study entry of CD4^+^ T-cells **(A)**, and of HIV DNA levels (expressed as log_10_ copies/10^6^ CD4^+^ T-cells) in non-compliant vaccinees and placebos **(B)**. The number of participants is as follows: non-compliant vaccinees year 1 n=17, year 3 n=17, year 4 n=10, year 11 n=12, year 12 n=6; non-compliant placebos year 1 n=5, year 3 n=5, year 4 n=4, year 11 n=2, year 12 n=0. Data are presented as mean values with standard error and a longitudinal analysis for repeated measurements was applied and *post-hoc* pairwise comparisons were conducted using the Tukey’s test. P-values assess the values at year 1–12 *vs*. baseline ISS T-003 trial values.

Of these, 22 participants (17 vaccinees and 5 placebos) were enrolled in the extended follow-up. Of note, no drug-level measurements were conducted in this study, and a detectable viral load was considered as the primary parameter of non compliance to ART. Over 12 years, vaccinees maintained stable CD4^+^ T-cell counts for the first 4 years, followed by significant and sustained increases by year 11 (+126 cells). In contrast, placebo participants showed marked declines from year 1 to year 4 (-51, -98, and -118 cells), with recovery only at year 11 (+245 cells) after switching to more effective ART (**Figure 5A**).

Non-compliant vaccinees also showed a continuous decline in HIV proviral DNA from year 1, reaching a 93% mean reduction (-1.17 log_10_) by year 11, whereas placebos exhibited a 11% reduction (-0.54 log_10_) only at year 11 (**Figure 5B**).

These results showed that non-compliant PWH may benefit most from Tat vaccination.

## Discussion

The extended follow-up of ISS T-003 trial participants presented here confirms that Tat vaccination exerts long-lasting ART intensification effects, persisting for more than a decade. Benefits were most pronounced in men, who were more immunocompromised at baseline, and in individuals with poor ART adherence. A summary of key immunological and virological findings from this study is presented in **Table 2**, and discussed below.

**Table 2:** Summary of key immunological and virological findings.

Domain	Finding	Comparison/effect	Notes
Anti-Tat antibodies	Long-term persistence and higher peaks titers in vaccinees as compared to anti-Tat Ab seroconverter placebos	No vaccine boosters	Consistent with previous phase 2 trial results in Italy
CD4^+^ T-cell recovery – overall	Significant increases from Year 1 through Year 12	Earlier and more sustained in vaccinees	Placebos improved mainly after ART regimen changes
CD4^+^ T-cell recovery – sex stratified	Strongest benefit among men	Vaccinated men showed continuous CD4^+^ increases; in placebo men CD4^+^ declined until late ART switch	Men had worse baseline immune status
CD4^+^ T-cell recovery – by viremia	Aviremic vaccinees had earlier and stronger gains	Intermittently viremic vaccinees maintained stability then improved	Placebos with viremia showed no immune recovery
Proviral DNA decay – overall	Significant reduction from Year 1 through Year 12; >90% mean reduction by Year 11 in vaccinees	Significant decay in vaccinees since year 1, while in placebos only after ART switch	Vaccinees showed early and continuous decline prior ART switch
Proviral DNA decay – sex stratified	Largest absolute declines in women; strongest *relative* benefit in men	Significant decay in vaccinees males since year 1, while placebo men showed significant decay but only after the second ART switch	Mirrors sex-related CD4^+^ trends
Proviral DNA decay – by viremia	Strong early decay in aviremic vaccinees	In viremic vaccinees proviral declined later but consistently	Placebos showed reduction only after ART changes
Viremia episodes	Similar proportion with ≥1 detectable episode	36% overall experienced ≥1 episode; 50% of men, regardless of treatment group	First late viremia events occurred only in placebos
Impact on poor adherence	Tat vaccine mitigated CD4^+^ decline and enhanced proviral DNA decay	In vaccinees stable or improving; in placebo worsening despite ART	Confirms initial 48-week findings
Mechanistic plausibility	Anti-Tat antibodies may block extracellular Tat and Tat–mediated HIV entry, reduce reservoir replenishment, and enhance clearance of infected cells, anti-Tat T-cell mediated response	Consistent with prior clinical and functional studies	Provides a coherent model for ART intensification

The immunological improvements in vaccinees coincided with durable anti-Tat Ab responses that persisted for up to 12 years without booster vaccination. These responses persisted significantly longer in vaccinees than in the 18 placebos who naturally developed anti-Tat Abs during the first 48 weeks of the ISS T-003 trial (21). Titers of individual anti-Tat Ig isotypes were also higher in vaccinees than in seroconverted placebos, indicating that vaccination is more effective at inducing a long-lasting and robust humoral response as compared to the immunity developing in some individuals in natural infection, also under ART. Persistence of vaccine-induced anti-Tat Ab is also consistent with results from a parallel phase-2 therapeutic clinical trial conducted in Italy (8, 9), as well as from phase 1 preventative and therapeutic trials, showing anti-Tat Ab persistence up to 5 years post-immunization (date of last determination) (19 and unpublished data). Of note, anti-Tat Abs durability appears to be an intrinsic characteristic of this vaccine and not to be stimulated by the infection, since it was detected after 5 years also in healthy volunteers immunized with Tat in a phase I preventative trial (24). Importantly, anti-Tat Abs, when present, are associated with a more benign disease course characterized by higher CD4^+^ counts, lower viral loads, and slower progression (13–16).

Tat vaccination accelerated and sustained CD4^+^ T-cell recovery compared with placebo. CD4^+^ T-cell increments in placebo participants mirrored those reported in large South African ART cohorts within the same period and under comparable treatment guidelines, which showed modest annual gains (~70 cells/mm³/year) (25–27) and greater increases following transition to dolutegravir-based regimens (+192 cells/mm³ over 3 years) (28). These findings underscore the ART intensification effect of Tat vaccination, which also markedly accelerated HIV proviral DNA decay. This was particularly evident at year 1, both in all volunteers and in males. While proviral DNA was not assessed in the parent ISS T-003 study, the present study shows that vaccinated participants experienced earlier and deeper HIV DNA reductions, while placebos showed significant declines only after ART regimen changes. These data are also consistent with findings of the parallel phase 2 therapeutic trial conducted in Italy with up to 8 years of follow-up, showing both persistent increases of CD4^+^ T cells and reduction of proviral HIV DNA, which were significantly associated with the presence of anti-Tat Abs (20). Of note, reports from rural South African cohorts on fixed-dose combination (FDC) ART show parallel CD4^+^ T-cell gains and reductions in total HIV-1 DNA (29). However, studies in South African women treated with the same FDC regimen found proviral DNA declines only during acute infection but not during chronic infection (30). Importantly, despite receiving less potent multi-pill ART in early follow-up, vaccinated participants exhibited CD4^+^ T-cell increases and proviral reductions earlier than placebo, whose improvements aligned temporally with guideline-mandated ART modifications.

It should be pointed out that no correlation was observed in this extended follow-up between anti-Tat Ab and CD4^+^ T-cell increases or HIV proviral DNA reduction, likely due to the loss of detectable Ab responses over the long-term follow-up and reduction in volunteers number over time. However, during the ISS T-003 trial, a significant inverse relationship between anti-Tat IgM or IgG Ab titers and neutralization of Tat-mediated HIV entry was observed in vaccinees, and CD4^+^ T-cell increases correlated significantly with neutralization of HIV entry in vaccinees as compared to placebos (21). Further, similar dynamics of CD4^+^ T-cell increases and proviral load decline were observed in the phase-2 therapeutic trial conducted in Italy with up to 8 years of follow-up (ISS T-002 EF-UP), which were significantly associated with the presence of anti-Tat Abs (20).

A strength of this study is the gender-stratified analysis, which is critical given the higher proportion of women enrolled and the well-documented influence of gender on ART outcomes. In sub-Saharan Africa, men living with HIV are less likely than women to receive ART (73% *vs* 83%) and achieve viral suppression (69% *vs* 79%) (4). Consistent with these epidemiological trends, male participants in our study had lower baseline CD4^+^ counts, higher proviral DNA levels, delayed HIV diagnosis, and more frequent episodes of detectable viremia. Importantly, male vaccine recipients derived the greatest benefit from Tat immunization, showing sustained CD4^+^ T-cell recovery and continuous proviral DNA decline, particularly after immunization (year 1), whereas male placebos displayed stable or increasing proviral loads with only modest reductions after ART regimen changes. These findings support previous observations indicating that even individuals with poor immune restoration do respond to Tat vaccination and experience the major benefits (8, 9, 20, 21).

Analysis by plasma viremia status, which was used as an indicator of reduced adherence to ART, further demonstrated that in aviremic participants, Tat vaccination promoted earlier and stronger CD4^+^ T-cell recovery and more pronounced proviral decay than placebos. In particular, the proviral DNA reduction was statistically different in aviremic vaccinees as compared to placebos either at year 1 or overall. Among individuals with intermittent viremia, CD4^+^ counts remained stable initially but increased significantly in vaccine recipients at later time points. Overall, viremic vaccinees showed significantly increased CD4^+^ T-cell counts compared with placebo recipients. Although proviral DNA levels did not differ markedly between viremic groups, CD4^+^ T-cell gains following the switch to dolutegravir-based ART were observed only in vaccinated individuals, suggesting that Tat immunization may enhance ART efficacy even in the presence of low-level viraemia.

The multiple pairwise comparisons of CD4^+^ T-cells and HIV proviral DNA changes from baseline by year and overall between vaccine and placebo groups indicate that differences of CD4^+^ T cells or proviral DNA are present in vaccinees versus placebos at different years as well as an overall effect, particularly when the analyses are done by sex or by the viremic status. These data support the notion that the effects of Tat immunization are more pronounced in the most immunocompromised subjects, i.e. male participants (who entered the study with lower CD4^+^ T cells and higher proviral DNA) and in volunteers with viremia, an indicator of reduced adherence to ART. However, the reduction in sample size over time limited the statistical significance of CD4^+^ T cells and proviral DNA differences between vaccinees and placebo recipients, although, the higher number of CD4^+^ T cells observed in vaccinees compared with placebos, even when not reaching statistical significance, remains clinically relevant, as these effects were detected in subjects undergoing long-term ART, when increases in CD4^+^ T-cell levels are typically modest or absent (31–33).

The violin plots confirmed that individual changes from baseline in CD4^+^ T-cell counts are more pronounced in vaccine recipients, and that nearly all vaccine recipients exhibit a decline in HIV proviral DNA, whereas some increases are observed in the placebo group.

Therefore, the sustained improvements in CD4^+^ T-cell counts and proviral DNA observed in vaccinated subjects compared with placebo recipients under comparable treatment conditions suggest a link with Tat vaccination, warranting further investigation. This is also consistent with findings from a parallel trial in Italy using the same vaccine in which the side-by-side evaluation of vaccinees versus the reference group showed durable improvements in immunological and virological parameters (9).

Tat vaccination also mitigated the detrimental effects of ART non-adherence. Consistent with prior 48-week data showing containment of viral rebound and CD4^+^ T-cell maintenance in non-compliant vaccinees (21), extended monitoring revealed sustained CD4^+^ T-cell increases in vaccine recipients, whereas placebo participants experienced marked CD4^+^ T-cell declines despite improved ART regimens, with only partial late recovery. Similarly, vaccinees exhibited continuous proviral DNA reductions, while placebos showed significant decreases only after switching to more potent regimens at year 11. These findings highlight the potential of Tat vaccination to buffer against the immunological and virological consequences of poor ART adherence.

The mechanisms underlying these ART-intensifying effects likely involve both induction of anti-Tat immunity and inhibition of extracellular Tat activity (34). Extracellular Tat, released from infected cells and bound to extracellular matrix components or circulating freely, promotes HIV replication, dissemination, and immune dysregulation, contributing to persistent immune activation and comorbidities in ART-treated individuals (34). Accordingly, the presence of anti-Tat Abs correlates with slower disease progression (14), delayed ART initiation (16), enhanced and sustained virological responses (8, 9, 17, 20, 34), and improved CD4^+^ T-cell recovery, particularly in vaccinees who developed all three classes of anti-Tat Abs (IgM, IgG, and IgA) (20). Tat also forms complexes with Env that mediate viral entry into integrin-expressing cells (12), key reservoirs during primary and chronic infection (9, 12). Anti-Tat Abs may prevent formation of these Tat/Env complexes, limiting infection of new target cells. Moreover, since we previously shown that HIV-1 sera regain neutralizing activity in the presence of anti-Tat Abs (12, 21), it is likely that anti-Tat Abs block the ongoing cell-to-cell virus transmission (35), thus reducing reservoir replenishment, and facilitating proviral decay (36). This then progressively reduces owing to the turnover of infected CD4^+^ T cells (36, 37), as suggested by the late proviral decay and its kinetics. Additionally, since extracellular Tat promotes differentiation of naïve CD4^+^ T cells into effector-memory subsets highly susceptible to HIV latency (37, 38), Tat vaccination may accelerate proviral DNA decay by hindering reservoir establishment and/or maintenance. Of note, in the phase-2 parallel trial conducted in Italy, the rate of HIV DNA decay in Tat vaccinees was markedly faster than that reported in other long-term ART studies, in which the estimated HIV DNA half-life ranged from 7 to 19 years (39, 40), compared with approximately 3 years in Tat vaccinees (20). Notably, the earliest, most pronounced, durable, and statistically significant reductions were observed in vaccinees who developed all three or at least two classes of anti-Tat Abs, starting as early as year 1 or year 3 after vaccination (20). Finally, the restoration of functional T cell subsets and NK cell counts seen early after Tat vaccination may render more effective immune recognition and clearance of infected cells, effectively contributing to proviral DNA decay (8, 9). Thus, the accelerated decay of total HIV DNA observed in vaccinated individuals is consistent with reduced reservoir replenishment combined with physiological turnover of infected CD4^+^ T cells, rather than rapid clearance of latently infected cells. This mechanism aligns with the progressive kinetics of proviral decline and with the observation that benefits were most evident in individuals with advanced immunosuppression or intermittent viremia, settings in which extracellular Tat activity is expected to be highest.

Collectively, these findings support a model in which therapeutic Tat vaccination acts both as an immunological modulator as well as by direct antiviral activities. By neutralizing extracellular Tat, vaccination may alleviate Tat-driven immune dysregulation, reduce aberrant activation of CD4^+^ T cells, and limit ongoing seeding and maintenance of the viral reservoir also during ART. The durable persistence of anti-Tat Abs (20, 24 and unpublished data), in the absence of booster immunizations, suggests the establishment of long-lived immune memory B cells capable of counteracting Tat-mediated pathogenic effects.

Despite substantial efforts, therapeutic vaccines designed to intensify ART have so far achieved limited success (6). Notably, none of these trials have been conducted in populations infected with C-clade HIV-1, the most prevalent strain globally and the dominant subtype in southern Africa. Moreover, gender-specific analyses have been largely absent, despite evidence that HIV outcomes are poorer among African men than women (7).

In this context, our previous work demonstrated that B-clade Tat therapeutic immunization in virologically suppressed, ART-treated South African adults infected with C-clade HIV induces cross-clade anti-Tat Abs, capable of neutralizing Tat-mediated B and C clade HIV (Env) entry in DC, suggesting that the B-clade Tat protein, used in our vaccine programs, can be used for a cross-clade HIV vaccine strategy (21). The Tat vaccine promotes CD4^+^ T-cell increases, particularly among individuals with low baseline CD4^+^ counts or poor ART adherence (21). This is clinically relevant, as suboptimal immune recovery despite viral suppression is common in people initiating ART late (41) or showing poor adherence (42), conditions linked to accelerated disease progression, comorbidities, hospitalization, and mortality (43, 44). These populations thus represent a key target for ART intensification strategies.

These findings hold particular relevance for sub-Saharan Africa, home to nearly two-thirds of the 40.8 million people living with HIV (4). Despite expanded ART access, HIV morbidity and mortality remain disproportionately high in this region, especially in South Africa, where the epidemic is severe and likely to worsen due to funding cuts affecting prevention, treatment, and access to long-acting formulations (4). Therapeutic HIV vaccination thus represents a promising, cost-effective adjunct to ART, capable of enhancing virological and immunological outcomes and mitigating the effects of poor adherence.

Limitations of this phase 2 study include its relatively small sample size and gender imbalance, which may limit the power of analyses, particularly at later time points, where the sample size is reduced. When the ISS T-003 trial was designed, sex-specific and precision medicine considerations were not yet standard. Nevertheless, our findings suggest that, in the context of C-clade infection, B-clade Tat immunization can effectively intensify ART responses and offset non-adherence, particularly in men who are typically more immunocompromised or in therapy non compliant PWH. These results support the need for larger, gender-balanced clinical trials of Tat vaccination in Africa, with stratification by gender and baseline immune status.

Thus, Tat vaccination represents a pathogenesis-driven immunotherapeutic strategy that complements ART by restoring immune homeostasis and progressively limiting reservoir maintenance, particularly in populations underserved by current treatment paradigms.

## Data Availability

The raw data supporting the conclusions of this article will be made available by the authors on reasonable request. However, data obtained from clinical study participants are protected for confidentiality reasons and can be made available only upon approval of a collaborative research proposal by the corresponding author, the ISS Ethical Committee, and the ISS Data Protection Officer. The research proposal must adhere to the Informed Consent signed by the participants.
