# Supplementary material for: Therapeutic HIV-1 Tat vaccination promotes durable immune reconstitution and reservoir reduction in ART-treated adults with clade C infection: a 12-year follow-up study
Source: Front Immunol. 2026 Apr 10;17:1769223. doi: 10.3389/fimmu.2026.1769223 (PMC13106519; doi:10.3389/fimmu.2026.1769223)
Supplement: Supplementary file 1 [file DataSheet1.zip › Data Sheet1/Appendix 1/ISS T-003 EFUP Protocol.pdf]

SPONSOR

AIDS National Center
Istituto Superiore di Sanità (ISS)
Viale Regina Elena, 299
00161 Rome, Italy

CLINICAL SITE

Mecru Clinical Research Unit
Sefako Makhatho Health Sciences University
Gauteng Province
South Africa

PRINCIPAL INVESTIGATOR

Prof. Maphoshane Nchabeleng

"A ROLL-OVER OBSERVATIONAL STUDY FOR THE EXTENDED FOLLOW-UP OF THE VOLUNTEERS OF THE ISS T-003 TRIAL" (ISS T-003 EF-UP)

Protocol Number: ISS T-003 EF-UP

Protocol Version: ISS T003 EF-UP Protocol Version 1.3, 15 January 2016

Confidentiality Statement

The information provided in this document is strictly confidential and is available for review to investigators, potential investigators, appropriate ethics committees and other national authorities. No disclosure should take place without the written authorization from *Istituto Superiore di Sanità*, except to the extent necessary to obtain informed consent from potential participants.

CLINICAL SITE AND PRINCIPAL INVESTIGATOR

Mecru Clinical Research Unit (MeCRU)
Sefako Makhatho Health Sciences University
Gauteng Province
South Africa
Principal Investigator: Prof. Maphoshane Nchabeleng

LIST OF OTHER PARTICIPANTS

Sponsor

National AIDS Center
Istituto Superiore di Sanità (ISS)
Viale Regina Elena, 299
00161 Rome, Italy
Tel. +39 06 49903209
Fax +39 06 49903002
Sponsor Responsible – Dr. Barbara Ensoli

Laboratory of Clinical Pathology and Microbiology

Ospedale S. Gallicano
Istituti Fisioterapici Ospitalieri
Via E. Chianesi, 53
00144 Rome, Italy
Tel. +39 06 5266 5245/6877
Fax +39 06 5266 6812
Laboratory Responsible – Dr. Fabrizio Ensoli

National Health Laboratory Service (NHLS)

Northern Branch
Dr George Mukhari Academic Hospital, Ga-Rankuwa, Pretoria
Tel: +27 (0)12 521 4282
Fax: +27 (0)12 521 4281

Contract Research Organization

Triclinium Clinical Development (TCD) (Pty) Ltd.
135 West Street
Sandown 2196
South Africa
Tel: +27 11 883 0206
Fax: +27 11 784 2818

EMERGENCY CONTACTS

Role in the study	Name	Telephone/Fax/e-mail
Sponsor<br>National AIDS Center<br>Istituto Superiore di Sanità<br>Rome, Italy.<br>(SA Head Office<br>c/o MRC-SAAVI - Cape Town)	Dr. Paolo Monini	Tel. +27 21 938 0205<br>Fax +27 21 938 0302<br>email: paolo.monini@iss.it
Principal Investigator<br>Mecru Clinical Research Unit<br>(MeCRU)	Prof. Maphoshane Nchabeleng	Tel: +27 12 521 5667<br>Fax: +27 12 521 5727<br>email: maphoshane.nchabeleng@smu.ac.za
CRO<br>Triclinium Clinical Development (Pty) Ltd.	Mr. Tebogo Magopane<br>(Project Manager)<br>Ms. Eleen Fourie<br>(Safety Reporting)	Tel: +27 11 883 0206<br>Fax: +27 11 7842 818<br>email: tebogo.magopane@triclinium.net<br>email: eleen.fourie@triclinium.net

TABLE OF CONTENTS

1 PROTOCOL SYNOPSIS 6
2 INTRODUCTION AND RATIONALE 7
2.1 Background and Rationale 7
3 STUDY DESIGN 9
3.1 Study Duration 9
4 METHODS 9
4.1 Inclusion Criteria 9
4.2 Exclusion Criteria 9
4.3 Randomization 9
4.4 Study Assessments 10
4.4.1 Clinical Assessments 10
4.4.2 Laboratory Assessments for Primary Endpoint Evaluations 10
4.4.3 Laboratory Assessments for Secondary Endpoint Evaluations 10
4.4.4 Additional Laboratory Evaluations 10
4.4.5 Sample Collection, Storage and Shipment 11
4.5 Schedule of Study Procedures 12
5 PREVIOUS AND CONCOMITANT MEDICATION 12
5.1 Antiretroviral Therapy 13
6 ENDPOINTS 13
6.1 Primary Endpoint Variable and Measurements 13
6.2 Secondary Endpoint Variables and Measurements 13
7 SAFETY 13
7.1 Safety Surveillance during the Study 13
7.2 Reporting of Serious Adverse Events 14
7.3 Local Medical Monitor (LMM) 14
7.4 Serious Adverse Event Treatment, Follow-up and Outcome 14
7.5 Reporting of Serious Adverse Events/Serious Adverse Drug Reactions 14
8 DATA RECORDING, MONITORING AND DATA MANAGEMENT 15
8.1 Data Recording 15
8.2 Data Monitoring 16
8.3 Data Management 16
9 STATISTICAL ANALYSIS 17
9.1 Statistical Program 17
9.2 Endpoints 17
9.3 Sample Size Determination 17
9.4 Methods 17
9.5 Primary Endpoint Analysis 17
9.6 Secondary Endpoint Analysis 17
9.6.1 Adjustments to Type 1 error 17
10 ETHICAL AND ADMINISTRATIVE PROCEDURES 18
10.1 Informed Consent 18
10.2 Ethical Issues and the Independent Ethics Committee 19
10.3 Participants Data Protection – Direct Access to Source Data 19
10.4 Insurance, Indemnity and Refunds 20
10.5 Audits and Inspections 20
10.6 Training of Study Staff 20
11 CASE REPORT FORMS & RECORD RETENTION 20
12 PUBLICATION OF RESULTS 21
13 STUDY TIMETABLE 21
14 INVESTIGATORS AGREEMENT 22
15 REFERENCES 23
16 APPENDIX I: SCHEDULE OF PROTOCOL VISITS & PROCEDURES 26

17 APPENDIX II: DECLARATION OF HELSINKI 27
Post-Trial Provisions 29
Research Registration and Publication and Dissemination of Results 29
Unproven Interventions in Clinical Practice 29
18 ENDIX IV: GLOSSARY 30

1 PROTOCOL SYNOPSIS

**Title:**<br>A roll-over observational study for the extended follow-up of the volunteers of the ISS T-003 trial
**Sponsor:**<br>Istituto Superiore di Sanità, National AIDS Center
**Study Objectives**:<br>Assessment of the persistence of vaccine immunogenicity as well as of the immunological and virological effects induced by the therapeutic immunization with Tat.
**Study Design**:<br>Open label, non-interventional study
**Planned Study Dates**:<br>Study start is estimated to be September-October 2014
**Study Duration**:<br>Enrolled patients will be followed-up for 52 weeks, with visits at weeks 0, 24 (Day 168) and 54 (Day 378)
**Study Population**:<br>Enrolment will be open to all patients who participated to the therapeutic phase II trial of the Tat vaccine “ISS T-003”.<br>Potentially all 200 patients enrolled in the ISS T-003 clinical trial
**Inclusion Criteria**:<br>All the following criteria have to be met for the patients to be eligible for the study;<br>1. previous participation in the ISS T-003 trial without withdrawal of consent;<br>2. availability to participate in the extended follow-up study;<br>3. signed informed consent.
**Exclusion Criteria**:<br>1. The absence of any of the above criteria will exclude the participants from the study.
**Statistical Analyses**:<br>All statistical analyses will be performed using SAS® version 9.2 or higher.<br>**Primary Immunogenicity Variable**<br>Presence of anti-Tat antibodies (IgM, IgG or IgA) as specific anti-Tat humoral immune response.<br><br>**Secondary Variables**<br>$CD4^+$ T cells and HIV RNA viral load will be evaluated and compared between anti-Tat antibody positive and negative participants.<br><br>Final analysis will be performed after all participants have completed the study and the database has been locked.

2 INTRODUCTION AND RATIONALE

South Africa is severely affected by HIV infection and AIDS. Implementation of National DOH strategic plans for screening the South African population for HIV infection (the HCT screening program) toward provision of combination antiretroviral treatment (cART) to all HIV-infected individuals with CD4 T cell counts <350 is steadily increasing the proportion of HIV-infected patients on therapy. However, this poses an enormous challenge to the public health system, also considering that recent epidemiological studies conducted in sub-Saharan Africa indicate that subtype C HIV infection is associated with a faster progression as compared with other virus subtypes, with a median time to progression and need of therapy between 2 and 3 years [1; *Abdool Karim Q, ICASA; Cape Town, 7-11 Dec 2013*]. Thus, the implementation of an effective care delivery is engulfing the public health system with a vast work overload and an increasing economic burden. In addition, despite the increasing access to cART, the rates of HIV morbidity/mortality are still high, patients' compliance to treatment is unsatisfactory and is associated with a 14% annual increase of HIV drug resistance [2]. A low adherence to cART also hampers an effective suppression of virus replication (i.e. viral load persistently undetectable), which, in turn, is a prerequisite to reduce virus transmission to healthy individuals [2].

In this context, an effective therapeutic HIV vaccine, in conjunction with existing strategies, may represent a relevant, cost-effective, contribution, either to control HIV infection by reducing time to progression and the need of therapy initiation or to increase the effectiveness of antiretroviral drugs by attaining a faster/more effective response to therapy (cART intensification), while reducing the negative impact of a low adherence to cART.

2.1 Background and Rationale

The development of a vaccine against HIV/AIDS has turned out to be extremely challenging, as indicated by almost 30 years of unsuccessful attempts. Recent advances in the understanding of the virus molecular immune pathogenesis have nevertheless lead to the development of novel vaccine concepts such as those based on HIV regulatory proteins. Targeting Tat represents a pathogenetic intervention against a key virulence factor, which plays pivotal roles in virus gene expression, replication, transmission and disease progression [3-10]. Tat is released extracellularly, accumulates in tissues and exerts effects on both the virus and the immune system [5-12] that make it an optimal vaccine candidate for therapeutic immunization [13-16]. Tat vaccination in monkeys can prevent or control infection with pathogenic SHIV [17], as recently confirmed by others, and found to correlate with anti-Tat antibodies [18-19]. Indeed, by binding Env spikes present on virus particles Tat forms a virus entry complex, which favors productive infection of DC and transmission to T cells by redirecting virus entry from the canonical receptors to integrins ($\alpha 5\beta 1$, $\alpha v\beta 3$, $\alpha v\beta 5$) [20]. Further, by binding the Env CCR5 co-receptor binding sites, Tat shields Env from anti-HIV-1 antibodies, thus inhibiting virus neutralization. However, either natural or vaccine-induced anti-Tat antibodies can restore and further increase HIV neutralization [20]. Accordingly, mucosal immunization of monkeys with combined Tat and oligomeric Env protects animals from intrarectal virus challenge, blocking virus spread to local lymph nodes [20]. Thus, Tat-specific antibodies appear to be key to prevent HIV acquisition and spreading.

In fact, anti-Tat antibodies, which are uncommon in natural infection, correlate with the asymptomatic state and lower disease progression [13-16].

In particular, a longitudinal study in Italy in a cohort of 252 HIV-1 seroconverters, with a median follow-up time of 7.2 years (range, 0.2-15.8 years), indicated that the presence of anti-Tat antibodies is predictive of a slower HIV disease progression to AIDS or severe immunodeficiency [13]. Progression was faster in the persistently anti-Tat antibody negative than in the transiently anti-Tat antibody positive subjects, while no progression at all was

observed in the persistently anti-Tat antibody positive individuals [13]. Cross-sectional studies have also shown a higher prevalence of anti-Tat antibodies in asymptomatic HIV-infected individuals than in patients in advanced disease stages [14-15] with an inverse correlation between anti-Tat humoral response and plasma viral load [14]. Further, the results of a prospective observational study (ISS OBS T-003, ClinicalTrials.gov NCT01029548) conducted in Italy on HIV-infected asymptomatic drug-naïve individuals, indicated that anti-Tat antibodies are associated with a containment of $CD4^+$ T cell loss and viral load increase up to three years of follow-up with a significant delay of disease progression [21]. Indeed, none of the patients persistently positive for anti-Tat antibodies started antiretroviral therapy during the three years of follow-up, while individuals with low or no anti-Tat antibodies had a median time to progression of 30 and 17 months, respectively [21]. In addition, a retrospective assessment of anti-Tat antibodies in subject enrolled in a clinical study on HAART efficacy, indicated that anti-Tat antibody positive individuals had statistically significant lower levels of plasma viraemia at enrolment and a faster and more effective kinetic of virus suppression after HAART initiation as compared to anti-Tat antibody negative subjects (*Ensoli F., unpublished observation*). Thus, the induction of anti-Tat immunity may help delay, and possibly block, HIV disease progression and ART implementation. This may be of outmost importance to allow a containment of the human, social and financial burden of health care delivery against HIV/AIDS**,** particularly in countries where new therapeutic interventions aimed at delaying drug usage and/or at improving response to therapy, while reducing virus transmission, are urgently needed. Finally, the presence of anti-Tat antibodies is associated with a more effective recovery of $CD4^+$ and $CD8^+$ T cells in ART-treated HIV^+ patients (ISS OBS T-002, ClinicalTrials.gov NCT01024556), particularly in subjects with high titers and persistent anti-Tab Ab (*manuscript in preparation*). These results provide a strong rationale for targeting Tat for both preventive and therapeutic immunization strategies [22-24].
Based on this notion, and on the results of pre-clinical testing and efficacy studies in monkeys [17-19], both preventative and therapeutic phase I trials [(ISS P-001, ClinicalTrials.gov NCT00529698; ISS T-001 ClinicalTrials.gov NCT00505401, respectively), were successfully conducted in Italy [22-29], allowing to advance the clinical development of the Tat vaccine to phase II studies in Italy and then in South Africa.
The ISS T-002 (ClinicalTrials.gov NCT00751595) was a randomized phase II open label therapeutic vaccine trial, which has been completed (2012) in 168 cART-treated individuals in 11 clinical sites in Italy. Participants were randomized to receive the biologically active HIV-1 Tat protein at 7.5 μg or 30 μg doses, given 3 or 5 times at monthly intervals. The results from this study indicated that Tat vaccination is safe and highly immunogenic. In addition, exploratory analyses suggested that Tat immunization exerts a positive impact on immune activation and T and B cell dysregulation as compared to subjects under effective HAART enrolled in a parallel observational study (ISS OBS T-002) (ClinicalTrials.gov NCT01024556) [29], confirming the role of Tat in the pathogenesis of HIV/AIDS. The longitudinal analysis (up to three years after Tat immunization) of ISS T-002, confirmed that immunization with Tat is safe and highly immunogenic and is capable of inducing a durable restoration of $CD4^+$ and $CD8^+$ T cell number and key functional T cell subsets, of B and NK cell number and a concomitant reduction of immune activation as compared to the reference group of subjects under effective HAART [29, 30]. Immune restoration started early after vaccination, increased upon time and was still effective for up to 3 years (corresponding to the end of the follow up period). Of importance, Tat immunization induced a statistically significant reduction of blood HIV-1 DNA load since week 72, which progressively further reduced up to week 144 (last time point). Effects were greatest with Tat 30 μg, given 3 times at monthly intervals, and under Protease Inhibitors (PI)-based regimens, with a predicted 70% HIV-1 DNA decay after 3 years from vaccination and a half-life of 88 weeks [30]. HIV-1 DNA decay was associated with anti-

Tat antibodies and neutralization of Tat-mediated entry of oligomeric Env in DC, which predicted HIV-1 DNA decay. Further, Tat-specific cellular responses also contributed, although to a lesser extent, to proviral reduction. These results indicate that the induction of anti-Tat immune responses is necessary to intensify HAART efficacy and to attack the ART-resistant virus reservoir. An observational study (ISS T-002 EF-UP, ClinicalTrials.gov NCT02118168) has been recently started to evaluate the persistence, magnitude and quality of the specific anti-Tat immune responses in individuals immunized with Tat. Immunological and virological parameters will be also monitored, including CD4+ T cells, HIV plasma viraemia and HIV proviral DNA.
Based on these results, a randomized, double-blind, placebo-controlled (randomized 1:1), confirmatory phase II therapeutic vaccine trial (ISS T-003, ClinicalTrials.gov NCT01513135) was conducted and recently completed in South Africa in 200 subjects under antiretroviral therapy in the frame of a bilateral cooperation agreement between the governments of South Africa and Italy. The Tat vaccine was administered at the most effective dosage tested in the T-002 phase II study (30 µg given three times intradermally) [29-30]. Trial database locking and unblinding were done end of July 2014, and final analyses and reporting has been completed by June 2015.

3 STUDY DESIGN

A roll-over observational study (ISS T-003 EF-UP) will be conducted to extend the follow-up of the volunteers of the ISS T-003 trial in order to evaluate the persistence of vaccine immunogenicity as well as of the immunological and virological effects induced by the therapeutic immunization with Tat.

3.1 Study Duration

Enrolled patients exiting study ISS T-003 will be enrolled to be followed-up for an additional 54 weeks, with visits at weeks 0, 24 and 54.

4 METHODS

4.1 Inclusion Criteria

All the following criteria have to be met for the patients to be eligible for the study;

1. previous participation in the ISS T-003 trial without withdrawal of consent;
2. availability to participate in the extended follow-up study;
3. signed informed consent.

4.2 Exclusion Criteria

The absence of any of the above criteria will exclude the participants from the study.

4.3 Randomization

At the time of enrolment, participants will keep the randomization code assigned at the inclusion in the ISS T-003 trial.

The original randomization list will be kept by Triclinium.

4.4 Study Assessments

The following section provides a detailed listing of the clinical, immunological and virological assessments to be performed during this study.

4.4.1 Clinical Assessments

Physical examination will be performed to monitor the clinical status of the volunteers and to record the appearance of clinical signs and symptoms of disease progression as well as AIDS-defining events, data or records related to ARV treatment compliance. Pregnancy onset for female participants will also be recorded.
All clinical assessments will be performed at the clinical site.

4.4.2 Laboratory Assessments for Primary Endpoint Evaluations

The assessments of anti-Tat humoral immune response will be performed at all visits by:

- Determination of IgM, IgG and IgA anti-Tat antibodies in serum
- Titration of IgM, IgG and IgA anti-Tat antibodies

4.4.3 Laboratory Assessments for Secondary Endpoint Evaluations

The following laboratory assessments will be performed at all visits:

- $CD4^+$ T cell counts
- HIV-1 plasma viraemia (viral RNA copies)

4.4.4 Additional Laboratory Evaluations

In addition, depending on the availability of residual specimens (PBMC, serum and plasma), the following tests will also be performed:

- HIV-1 proviral DNA
- Epitope mapping of serum IgM and IgG anti-Tat antibodies
- Functional serum anti-Tat antibodies capable of neutralizing Tat-mediated virus entry in DC, measured as Tat-mediated entry of the trimeric Env protein in DC
- Serum IgM, IgG and IgA anti-Tat cross-clade antibodies (C, D, A, AG)
- Characterization of lymphocyte subsets (CD3, CD4, CD8, CD16, CD56, CD19)
- Anti-HIV regulatory and structural proteins antibodies
- Antibody-mediated cellular cytotoxicity (ADCC)
- Neutralization of primary isolates (all clades)
- Anti-CCR5 antibodies
- Anti-CD4 antibodies
- Lymphoproliferative response to mitogens and recall antigens
- Lymphoproliferative response to HIV-1 Env (CFSE staining)
- *In vitro* γIFN, IL-4 and IL-2 production in response to Env (ICS/Elispot)
- B cells phenotype (naïve and memory) and antigen specific antibodies
- Phenotype and functional characterization of regulatory T cells
- Intracellular PBMC staining for granzyme, perforin, cytokines and chemokines
- Analysis of Th1 and Th2 cytokines in sera and in PBMC supernatants
- Analysis of chemokines in sera and in PBMC supernatants

- Lymphocytes spontaneous cell death and PBMC cell viability
- B cell cloning
- Functional and molecular characterization of clono-specific antibodies
- Serum/plasma determination of soluble CD4
- Serum/plasma determination of phenotypic and biochemical markers of immune activation (CD38 expression, neopterin, β2-microglobulin, C reactive protein, total immunoglobulin, sCD27, sCD14, LPS and IgM against endotoxin core antigen by ELISA)
- HIV-1 sequencing and phylogenetic analysis
- HIV-1 genotypic resistance
- HIV viral tropism
- Assessment of co-infections (e.g. Syphilis, HBV, HPV, HHV8 and others)

4.4.5 Sample Collection, Storage and Shipment

Samples will be collected at 3 different time points (weeks 0, 24 and 54), resulting in the collection of approximately 148.5 ml blood over a period of at least 54 weeks. All blood samples will be collected by venipuncture.

The $CD4^+$ T cell counts and HIV-1 plasma viraemia will be performed at the NHLS Laboratory of the DGM Hospital. Approximate volumes for these assessments are 9 ml at each study visit. Blood samples for these tests will be collected at the Clinical Research Unit and transferred to the laboratory so that the testing procedures begin within three hours of sample collection. Results will be forwarded to the investigator as soon as available, in accordance with test-specific turn-around-times.

Specialised immunological and virological evaluations, including anti-Tat humoral immune response, HIV-1 proviral DNA and the laboratory evaluations detailed in sections 4.4.3 will be performed at the *Laboratory of Clinical Pathology and Microbiology* (*Ospedale S. Gallicano IFO, Rome)*. Approximate volumes for the assessments are 49.5 ml at each study visit.

Blood samples to be transferred to the NHLS and/or to the *Laboratory of Clinical Pathology and Microbiology* for immunologic and virologic investigations will be collected and processed according to the procedures described by the protocol-specific Specimen Management Manual which will be provided to the investigational site prior to study initiation. Thereafter, cryopreserved cellular specimens and sera will be stored in liquid nitrogen or at -80 °C prior to be shipped to the *Laboratory of Clinical Pathology and Microbiology* (IFO San Gallicano Hospital, Rome). Shipment to the Laboratory will be done on dry ice, through a specialized courier, within 15 days of the date of sample collection.
Each sample will be identified with the following information: protocol number, centre number, participant number (i.e. participant identifier), visit number, sample type (plasma, serum or whole blood) and the sample collection date.

The residual specimens (PBMC, serum or plasma) available after the conduct of the first line tests will either be used immediately, or kept frozen for later use for the determination of additional laboratory tests (refer to section 4.4.3).

4.5 Schedule of Study Procedures

The schedule of study visits and assessments is as follows:

DAY 0 (Baseline)

- Review of medical history since the last visit for the ISS T-003 trial
- Physical examination including weight and vital signs (seated blood pressure, heart rate, respiratory rate and axillary body temperature)
- Concomitant medications, including ARV changes and compliance
- Anti-Tat antibodies (IgM, IgG, IgA)
- CD4+ T cell counts
- HIV-1 plasma viraemia
- Immunological & virological sample collection and storage.

DAY 168: Week 24 (Day 168 ± 28 days)

- Physical examination including weight and vital signs (seated blood pressure, heart rate, respiratory rate and axillary body temperature)
- Concomitant medications, including ARV compliance
- Anti-Tat antibodies (IgM, IgG, IgA)
- CD4+ T cell counts
- HIV-1 plasma viraemia
- Immunological & Virological sample collection and storage

DAY 378: Study termination Week 54 (Day 378 ± 28 days)

- Physical examination including weight and vital signs (seated blood pressure, heart rate, respiratory rate and axillary body temperature)
- Concomitant medications, including ARV compliance
- Anti-Tat antibodies (IgM, IgG, IgA)
- CD4+ T cell counts
- HIV-1 plasma viraemia
- Immunological & Virological sample collection and storage

If a participant misses a scheduled study visit, the study staff will try to establish communication with the participant through all possible means (e.g., writing and telephoning the participants and his/her contacts). The need to attend study visits will be emphasized.
If a participant misses a scheduled visit the study staff will try to reschedule the visit within 4 weeks.

5 PREVIOUS AND CONCOMITANT MEDICATION

All previous and concomitant treatments (from last T-003 visit attended to Day 0) will be recorded in the CRF, including the name of the treatment (if pharmacological, only generic names are required unless it is a combination product where the trade name should be documented), indication, route, total daily dose and start/stop dates.

5.1 Antiretroviral Therapy

Participant should receive standard antiretroviral therapy in accordance with their clinical condition and local, current guidelines.
As far as possible, a complete antiretroviral treatment history, starting from the last visit performed for the ISS T-003 trial, must be recorded in the CRF.

6 ENDPOINTS

6.1 Primary Endpoint Variable and Measurements

In order to evaluate the persistency of Tat vaccine immunogenicity, the following primary endpoint will be considered:

- Presence of specific anti-Tat humoral immune response in terms of IgM, IgG or IgA anti-Tat antibodies.

The persistence of the humoral response will be compared between the active and placebo groups.

The anti-Tat specific **humoral immune response** will be evaluated in the active as compared to the placebo group as follows:

1. Percentage of "responders", defined as those participants with an immune response greater than the following levels:
    - *Anti-Tat IgM titers ≥ 25*
    - *Anti-Tat IgG titers ≥ 100*
    - *Anti-Tat IgA titers ≥ 25*
2. Geometric mean antibody titers (GMT) induced by vaccination.

6.2 Secondary Endpoint Variables and Measurements

The following secondary endpoints will be considered and compared between active and placebo groups:

- $CD4^+$ T cell counts and changes from the study entry
- Levels and changes of HIV-1 RNA plasma viraemia

7 SAFETY

7.1 Safety Surveillance during the Study

Only serious adverse events (SAE) will be collected during the study and evaluated by the study local medical monitor.

Seriousness refers to the outcome of an adverse event. Seriousness is determined by the principal investigator (or qualified designee). If any of the following outcomes are present then the adverse event is serious:

- Results in death

- Is life-threatening (defined as an event in which the participant or patient was at risk of death at the time of the event; it does not refer to an event which hypothetically might have caused death if it were more severe)
- Requires inpatient hospitalization or prolongation of existing hospitalization
- Results in persistent or significant disability/incapacity
- Is a congenital anomaly/birth defect
- Is an important medical event [defined as a medical event that may not be immediately life-threatening or result in death or hospitalization but, based upon appropriate medical and scientific judgment, may jeopardize the participant or may require intervention (e.g. medical, surgical) to prevent one of the other serious outcomes listed in the definition above]. Examples of such events include, but are not limited to, intensive treatment in an emergency room or at home for allergic bronchospasm, blood dyscrasias or convulsions that do not result in hospitalization.

7.2 Reporting of Serious Adverse Events

The identification and reporting of SAE is the Investigator's responsibility.
The SAE may be either spontaneously reported or elicited during questioning and examination of a participant. All identified SAEs related to the study drug must be recorded and described on the appropriate page of the CRF in occasion of the scheduled visit. If known, the diagnosis of the underlying illness or disorder should be recorded, rather than its individual symptoms. Any clinically significant changes noted during interim or final physical examinations and any other potential safety assessments, whether or not these procedures are required by the protocol, should also be recorded on the appropriate page of the CRF.

Pre-existing conditions (absent at the time of the last visit of the ISS T-003 study) should be recorded in the CRF as baseline medical history.

7.3 Local Medical Monitor (LMM)

The LMM is the sponsor's representative and is a registered medical practitioner in his/her country of residence with experience in HIV/AIDS related research and HAART treatment. The LMM may assist the Principal Investigator in the assessment of serious adverse events.

7.4 Serious Adverse Event Treatment, Follow-up and Outcome

Treatment of collected SAEs will be determined by the investigator using his/her best medical judgment and according to current clinical practice guidelines. All applied measures as well as follow-up will be recorded in the appropriate CRF.

Follow-up for collected SAEs must continue until resolution and the outcome reported to ISS, even if this extends beyond the serious adverse event reporting period (i.e., after the final study visit).

7.5 Reporting of Serious Adverse Events/Serious Adverse Drug Reactions

Collected SAEs, which include Suspected Unexpected Serious Adverse Reaction (SUSAR), are to be reported to the sponsor for the entire study period. SUSARs are reported even after the trial is over, if the sponsor, local medical monitor or principal investigator becomes aware of them. The site will be provided with specific reporting procedures including the SAE CRF and any supplemental reporting forms to be used. Serious

adverse events will be reported on the SAE CRF using a recognized medical term or diagnosis that accurately reflects the event.

SAEs will be assessed by the investigator for severity, causal relationship to the study vaccine, and expectedness. The onset and resolution dates of the event and the action taken in response to the event will be documented. If the event has not resolved by the final study visit, it will be documented as "ongoing" on the CRF, however, follow-up of the SAE will continue for as long as practically feasible until resolved. Information recorded on the CRF must be substantiated in the source documents.

The Report of SAE related to the study drug completed for that event must be **faxed** by the principal investigator or his/her designee **within 24 hours** (one calendar day) of the investigative site becoming aware of the event to Triclinium.

Contact information for all safety personnel are contained in the Team Contact List which will be stored on site in the investigator Site File and maintained by Triclinium.

Investigators **must not wait** to collect additional information to fully document the event before notifying the local medical monitor and Triclinium of a related SAE. The initial notification will include the following (at minimum):

- Protocol number and name and contact number of the investigator
- Participant study number (and initials and date of birth, if available)
- Date/s participant received study vaccine
- Related Serious adverse event(s) and date of event onset
- Current status of participant

ISS has authorized Triclinium to execute its responsibilities for safety report submission to the appropriate regulatory authorities within specific time periods of being notified of the event; the investigative site is responsible for notification of all SAE reports to the relevant Ethics Committee within the appropriate reporting time periods. Triclinium will notify the DSMB of all SUSAR within three working days of becoming aware of an event and will provide all follow-up information in a timely manner.

8 DATA RECORDING, MONITORING AND DATA MANAGEMENT

8.1 Data Recording

Case Report Forms

All data generated by the study will be recorded on the CRFs provided. All original entries will be initialled and dated by the individual conducting the assessment. The forms will be completed in black ink (hard point pen).

Any correction or deletion should be made by drawing a single line through the entry so that the original entry is still legible. This change must be initialled and dated by the site study coordinator or Investigator. Copies of the forms should be retained by the Principal Investigator, and must be made available for review by the Sponsor, Institutional Review Board (IRB), or appropriate regulatory agencies.

Any changes made to data after collection of the CRF pages by Data Management will be made through the use of Data Clarification Forms (DCF). Data reported on the CRFs which are derived from source documents should be consistent with the source documents or the discrepancies should be explained. Case Report Forms will be considered complete when all missing and/or incorrect data have been resolved.

Source Documentation
Source documents are considered to be all information in original records and certified copies of original records of clinical findings, observations, data or other activities in a clinical study necessary for the reconstruction and evaluation of the study.
Source documents will be maintained for all data collected in the CRFs.
Investigators are required to prepare and maintain adequate and accurate case histories designed to record all observations and other data pertinent to the investigation on each individual entered into the study. Data reported on the Case Report Form (CRF) must be consistent with the source documents.

8.2 Data Monitoring

This study will be conducted in accordance with the principles of Good Clinical Practice. Before roll-over study initiation, Triclinium personnel will visit the clinical site, discuss with the investigator(s) (and other personnel involved in the study) their responsibilities with regard to protocol adherence, and the responsibilities of Sponsor or its representatives.

During the study, a Clinical Trial Monitor will have regular contacts with clinical site, including visits to:

- provide information and support to the investigator(s)
- confirm that facilities remain acceptable
- confirm that the investigational team is adhering to the protocol, that data are being accurately recorded in the case report forms
- perform source data verification (a cross-check between data recorded in CRF and participants’ medical records and/or other records relevant to the study). This will require direct access to all original data for each participant (e.g. clinical records).

8.3 Data Management

Independent, double-data entry will be performed into an electronic database by two different data capturers. Verification of the data will be performed after a comparison of the two entries. Data validation will include checks of limits, codes, missing data and logical inter- and intra-table consistency. Irresolvable inconsistencies will be referred to the investigational team for clarification. Upon completion of all validation processes, and after a blind review of the data, the database will be “locked”. Data will be extracted from the database directly into data files for statistical analyses.

Modifications and updates to the database will be documented in an audit trail. All data management activities will be performed in a clinical data management system that is fully compliant with international and local guidelines and regulations pertaining to data management.

Medical and surgical history, concomitant illnesses and adverse events will be coded using the Medical Dictionary for Regulatory Activities (MedDRA) Version 12 or higher. Previous and concomitant medication taken at any stage during the study will be coded using the MIMS classification.

9 STATISTICAL ANALYSIS

9.1 Statistical Program

Statistical analyses will be performed using SAS® (Version 9.2 or higher, SAS Institute, Cary, NC, USA).
Final analysis will be performed after all participants have completed the study and the database has been locked.

9.2 Endpoints

Primary Endpoint Variable

- Presence of anti-Tat antibodies (IgM, IgG or IgA)

Secondary Endpoint Variables

- $CD4^+$ T cell counts
- Levels of HIV-1 RNA plasma viraemia
- HIV-1 proviral DNA copies

9.3 Sample Size Determination

For this observational study no sample size will be predefined due to the fact that the study represents an extended follow-up of the therapeutic ISS T-003 phase II clinical trial, mainly aimed at evaluating the persistence of the immune responses in immunized participants versus placebo.
Potentially a total of 200 participants will be enrolled into the study.

9.4 Methods

Statistical analysis and data processing will be performed using SAS® software for Windows.
All descriptive statistics used to summarize numeric data will include mean values, standard deviation, standard error, median, minimum and maximum. Frequency distributions will be presented for categorical variables. 95% confidence intervals will be determined for all data. All statistical tests will be performed at two-sided with a 5% significance level.

9.5 Primary Endpoint Analysis

Anti-Tat humoral immune response will be evaluated by the percentage of Ab positive participants with the relative 95% confidence interval; geometric mean antibody titers (GMT) will be also performed.
The duration of the anti-Tat antibody response will be determined and compared among treatment groups by the Log-Rank test.

9.6 Secondary Endpoint Analysis

$CD4^+$ T cells and HIV RNA viral load will be evaluated and compared between the participants that received the Tat vaccine or placebo in the ISS T-003 study using an Analysis of variance model (ANOVA).
A Multivariate analysis will be applied to the immunological and virological parameters in order to carry out potential prognostic factors for the clinical outcome.

9.6.1 Adjustments to Type 1 error

Since no formal treatment group comparison will be performed on the humoral immune response data, it will not be necessary to adjust for any inflation of the overall Type 1 error rate.

10 ETHICAL AND ADMINISTRATIVE PROCEDURES

10.1 Informed Consent

Preparation of the consent form is the responsibility of the Sponsor with the support of the CRO. The principles of informed consent in the current edition of the Declaration of Helsinki will be implemented. Informed consent will be obtained from each participant in accordance with the Declaration of Helsinki and ICH-GCP prior to performing any protocol-specified procedures.

Informed consent will be documented in writing. The written Participant Information and Informed Consent form will be approved by the IEC. The written consent document will embody the elements of informed consent as described in the Declaration of Helsinki and will also comply with local regulations.

The investigator or the investigator's qualified designee will provide all relevant information in both oral and written form in a way that is understandable to the participant. Ample time and opportunity must be given for the participant to inquire about details of the study. The participant must be informed about the study's purpose including why the participant was selected to participate, study goals, expected benefits and risks, potential risks, and that some potential risks are unforeseeable. The individual must be provided with a description of the procedures and the estimated duration of time required for participation in the study, as well as alternative interventions or courses of treatment, if applicable.

The participant must receive an explanation as to whether any compensation and any medical treatments are available if injury occurs and, if so, what they are; where further information may be obtained, and who to contact in the event of a study-related injury. Individuals must be told who to contact for answers to any questions related to the study.

The participant must be informed that their participation is voluntary and that they are free to withdraw from the study for any reason at any time without penalty or loss of benefits to which they are otherwise entitled. The extent of the confidentiality of participant records must be defined and the participant must be informed that applicable data protection legislation will applies.

The participant must be informed that the study monitor(s), auditor(s), and the applicable regulatory authorities and Ethics Committees will be granted direct access to the participant's original study medical records for verification of protocol-specified procedures and/or data, without violating the confidentiality of the participant to the extent permitted by the applicable laws and regulations.

The participant must be informed that his/her signature on the informed consent form indicates that he/she has decided to participate in the study, having read and discussed the information presented.

The informed consent and any other information provided to the participants, should be revised whenever important new information becomes available that is relevant to the participant's consent, and should receive IEC approval/ prior to use. The Investigator or the investigator's qualified designee will fully inform the participant of all pertinent aspects of the study and of

any new information relevant to the participant's willingness to continue participation in the study. This communication will be documented.

The original, signed informed consent form for each participant will be maintained by the investigator as part of the participant's study records. A copy of the signed informed consent form will be provided to each participant.

10.2 Ethical Issues and the Independent Ethics Committee

This study will be conducted in accordance with the protocol and any applicable amendments, the Declaration of Helsinki, Seoul 2008, ICH GCP, July 2002 and the Guidelines for Good Practice in the Conduct of Clinical Trials in Human Participants in South Africa, 2006.

The protocol and informed consent form will be reviewed and approved by the IEC prior to any protocol-specified procedures being conducted.
The investigator will inform the IEC as to the progress of the study on a regular basis, or at minimum, once a year.

All the documents the IEC may need to fulfil its responsibilities, such as the protocol, protocol amendments, information concerning participant recruitment, payment or compensation procedures, etc., will be submitted to the IEC by the investigator. Written, unconditional approval of the study protocol and the informed consent form by the IEC will be in the possession of the Investigator /clinical site staff prior to the conduct of any protocol-specified procedures.

Amendments to the protocol may not be implemented without prior written IEC approval except when necessary to eliminate immediate hazards to a participant or when the amendment involves only logistical or administrative aspects of the study. Such logistical or administrative amendments will be submitted to the IEC in writing by the investigator, and a copy of the correspondence to verify the submission will be maintained.

The investigator must inform the IEC of amendments to the informed consent or any other documents previously submitted for review/approval, of any new information that may adversely or otherwise affect the safety of the participants or the conduct of the study, provide an annual update and/or request for re-approval, and advise the IEC when the study has been completed.

Any documents to be provided to the participant (e.g., information cards, letters from the investigator), and all forms of study advertising (flyers, brochures, print advertisements, radio or television scripts, etc.) must be approved by ISS or its designee prior to the clinical site submitting them to the IEC. Approval from the IEC must be obtained prior to providing the documents to the participant, or utilization of the study advertisement.

10.3 Participants Data Protection – Direct Access to Source Data

The rights, safety and well-being of the individual study participants are the most important considerations and should prevail over interests of science and society.
Study personnel involved in conducting this trial will be qualified by education, training, and experience to perform their respective tasks.
This study will not use the services of study personnel where sanctions have been invoked or where there has been scientific misconduct or fraud (e.g., loss of medical licensure, debarment).

10.4 Insurance, Indemnity and Refunds

With respect to any liability directly or indirectly caused by participation in this clinical trial, the Sponsor assumes liability on behalf of the investigators for possible injury to the participant, provided the investigator has followed the instructions of the Sponsor in accordance with this protocol and any amendments thereto, and that the investigator has performed the observational study in accordance with scientific practice and currently acceptable techniques and knowledge. The Sponsor's liability is covered by liability insurance.

10.5 Audits and Inspections

Authorized representatives of ISS, Regulatory authorities and Independent Ethics Committees may visit the clinical trial site to perform audits or inspections, including source data verification. The purpose of any audit or inspection is to systematically and independently examine all study related activities and documents in order to determine whether these activities were properly conducted, and data were recorded, analyzed, and accurately reported according to the protocol, Good Clinical Practice (GCP), the guidelines of the International Conference on Harmonisation (ICH), and applicable regulatory requirements. The investigator should notify ISS immediately if contacted by a regulatory agency about an inspection at his/her trial site.

10.6 Training of Study Staff

The Principal Investigator will maintain training records for all individuals involved in the study (medical, nursing and other staff). He/she will ensure that appropriate training is given to all staff, and that any new information of relevance to the conduct of this study is provided to the staff involved.

11 CASE REPORT FORMS & RECORD RETENTION

Data recorded on source documents will be transcribed onto ISS approved case report forms (CRFs) provided by Triclinium. Completed, original CRFs will be retrieved by Triclinium and a copy of each completed CRF will be retained at the clinical site as part of the study records. The study will be monitored regularly by Triclinium throughout the study period.

All study records (source documents, signed informed consent forms, copies of CRFs, Regulatory Authority and IEC correspondence and approval letters, study vaccine management records) will be kept secured for a minimum period 2 years after the last approval of a marketing application in an ICH region and until there are no pending or contemplated marketing applications in an ICH region or after the formal discontinuation of the clinical development of the investigational product.

The investigator will ensure that study records are not disposed of or removed from the clinical site without prior notification and approval from ISS. ISS will notify the Investigator when retention of the trial records is no longer required.

If an Investigator withdraws from the study prematurely (e.g., relocation, retirement) the study records will be transferred to a mutually agreed designee (e.g., another Investigator, Ethics Committee). Notice of such transfer will be given in writing to ISS.

12 PUBLICATION OF RESULTS

All information regarding this study or obtained as a result of this study is regarded as confidential; by signature of this protocol, the Investigators agree that the scientific results of this study are the property of ISS.

Unpublished information contained herein, as well as any information received from the Sponsor for the purposes of this study, may not be disclosed to any third party without the prior written approval of the Sponsor. No data may be used for presentation at scientific meetings and/or publication in scientific journals without the prior written authorization from the Sponsor. The investigators and study site staff must send all manuscripts, abstracts, and presentations using data from this study to ISS for review prior to their submission. ISS reserves the right to delete any part or parts of such materials deemed to be confidential or proprietary.

ISS reserves the right to use the results of this trial for scientific applications and/or submissions to Regulatory Authorities. ISS is committed to publication of the results of this study after study conclusion.

13 STUDY TIMETABLE

Volunteers who participated to the ISS T-003 trial will be invited to adhere to the present study. The onset of enrolment is planned in September-October 2014. The investigators are expected to make every reasonable effort to recruit suitable participants into the study. If the enrolment rate is low, or if a significant number of protocol violators are recruited or if no participants are recruited within a reasonable period, ISS reserves the right to prematurely close enrolment or pause or terminate the study.

14 INVESTIGATORS AGREEMENT

A ROLL-OVER OBSERVATIONAL STUDY FOR THE EXTENDED FOLLOW-UP OF THE VOLUNTEERS OF THE ISS T-003 TRIAL

Protocol Number: ISS T-003 EF-UP

Protocol Version: 15 January 2016

Sponsor Representative

Barbara Ensoli, MD, PhD	Signature	Date

AIDS National Center
Istituto Superiore di Sanità
Viale Regina Elena, 299
00161 - Rome, Italy

Clinical Site - Principal Investigator

I have carefully read this protocol and agree to conduct the study in accordance with GCP, Declaration of Helsinki, local laws and regulations relevant to the use of new and approved therapeutic agents in human participants.
I agree that ISS, its delegates and Regulatory Authorities have direct access to all study documentation.
I agree to obtain Written Informed Consent from all participating volunteers or their legal representative.
I agree to maintain the confidentiality of all information received or developed in connection with this protocol.

Prof. Maphoshane Nchabeleng	Signature	Date

Mecru Clinical Research Unit (MeCRU)
Sefako Makhatho Health Sciences University,
Gauteng Province
South Africa

15 REFERENCES

1. Amornkul PN, Karita E, Kamali A, Rida WN, Sanders EJ, Lakhi S et al. Disease progression by infecting HIV-1 subtype in a seroconverter cohort in sub-Saharan Africa. AIDS 2013; 27:2775-86.
2. Gupta RK, Jordan MR, Sultan BJ, Hill A, Davis DH, Gregson J, et al. Global trends in antiretroviral resistance in treatment-naive individuals with HIV after rollout of antiretroviral treatment in resource-limited settings: a global collaborative study and meta-regression analysis. Lancet 2012; 380:1250-8.
3. Ensoli B, Barillari G, Salahuddin SZ, Gallo RC, Wong-Staal F. Tat protein of HIV-1 stimulates growth of cells derived from Kaposi's sarcoma lesions of AIDS patients. Nature 1990; 345:84-86.
4. Ensoli B, Markham P, Fiorelli V, Colombini S, Raffeld M, et al. Synergy between basic fibroblast growth factor and HIV-1 Tat protein in induction of Kaposi's sarcoma. Nature 1994; 371:674-680.
5. Ott M, Emiliani S, Van Lint C Herbein G, Lovett J, Chirmule N, et al. Immune hyperactivation of HIV-1-infected T cells mediated by Tat and the CD28 pathway. Science 1997; 275:1481-1485.
6. Sigal A, Kim JT, Balazs AB, Dekel E, Mayo A, Milo R et al. Cell-to-cell spread of HIV permits ongoing replication despite antiretroviral therapy. Nature 2011; 477: 95-98.
7. Chang HC, Samaniego F, Nair BC, Buonaguro L, Ensoli B. HIV-1 Tat protein exits from cells via a leaderless secretory pathway and binds to extracellular matrix-associated heparan sulfate proteoglycans through its basic region. AIDS 1997; 11:1421-1431.
8. Rayne F, Debaisieux S, Yezid H, Lin YL, Mettling C, Konate K, et al. Phosphatidylinositol-(4,5)-bisphosphate enables efficient secretion of HIV-1 Tat by infected T-cells. EMBO J 2010; 29:1348-1362.
9. Johnson TP, Patel K, Johnson KR, Maric D, Calabresi PA, Hasbun R et al. Induction of IL-17 and nonclassical T-cell activation by HIV-Tat protein. Proceedings of the National Academy of Sciences 2013; 110: 13588–13593.
10. Fanales-Belasio E, Moretti S, Nappi F, Barillari G, Micheletti F, Cafaro A, et al. Native HIV-1 Tat protein targets monocyte-derived dendritic cells and enhances their maturation, function, and antigen-specific T cell responses. J Immunol 2002; 168(1):197–206.
11. Huang L, Bosch I, Hofmann W, Sodroski J, Pardee AB. Tat protein induces human immunodeficiency virus type 1 (HIV-1) coreceptors and promotes infection with both macrophage-tropic and T-lymphotropic HIV-1 strains. J Virol 1998; 72:8952–8960.
12. Mediouni S, Darque A, Baillat G, Ravaux I, Dhiver C, Tissot-Dupont H, et al. Antiretroviral therapy does not block the secretion of the human immunodeficiency virus Tat protein. Infect Disord Drug Targets 2012; 12:81-86.
13. Rezza G, Fiorelli V, Dorrucci M, Ciccozzi M, Tripiciano A, Scoglio A, et al. The presence of anti-Tat antibodies is predictive of long-term nonprogression to AIDS or severe immunodeficiency: findings in a cohort of HIV-1 seroconverters. J Infect Dis 2005; 191:1321-1324.
14. Re MC, Vignoli M, Furlini G, Ghibellini D, Colangeli V, Vitone F, et al. Antibodies against full-length Tatprotein and some low-molecular-weight Tat-peptides correlate with low or undetectable viral load in HIV-1 seropositive patients. J Clin Virol 2001; 21:81–9.

15. Reiss P, Lange JM, de Ronde A, de Wolf F, Dekker J, Debouck C, et al. Speed of progression to AIDS and degree of antibody response to accessory gene products of HIV-1. J Med Virol 1990; 30:163–8.
16. Richardson MW, Mirchandani J, Duong J, Grimaldo S, Kocieda V, Hendel H, et al. Antibodies to Tat and Vpr in the GRIV cohort: differential association with maintenance of long-term non-progression status in HIV-1 infection. Biomed Pharmacother 2003; 57:4–14.
17. Cafaro A, Caputo A, Fracasso C, Maggiorella MT, Goletti D, Baroncelli S et al. Control of SHIV-89.6P-infection of cynomolgus monkeys by HIV-1 Tat protein vaccine. Nat Med 1999; 5: 643-650.
18. Cafaro A, Bellino S, Titti F, Maggiorella MT, Sernicola L, Borsetti A, et al. Impact of viral dose and major histocompatibility complex class IB haplotype on viral outcome in Tat-vaccinated mauritian cynomolgus monkeys upon challenge with SHIV89.6P. J. Virol 2010; 84(17): 8953-8958.
19. Bachler BC, Humbert M, Palikuqi B, Siddappa NB, Lakhashe SK, Rasmussen RA, et al. Novel Biopanning Strategy To Identify Protection Epitopes Associated with Vaccine. J Virol 2013; 87(8):4403.
20. Monini P, Cafaro A, Srivastava IK, Moretti S, Sharma VA, Andreini C et al. HIV-1 Tat Promotes Integrin-Mediated HIV Transmission to Dendritic Cells by Binding Env Spikes and Competes Neutralization by Anti-HIV Antibodies. PLoS One 2012; 7:e48781.
21. Bellino S, Tripiciano A, Picconi O, Francavilla V, Longo O, Sgadari C, Paniccia G, Arancio A, Angarano G, Ladisa N, Lazzarin A, Tambussi G, Nozza S, Torti C, Focà E, Palamara G, Latini A, Sighinolfi L, Mazzotta F, Di Pietro M, Di Perri G, Bonora S, Mercurio VS, Mussini C, Gori A, Galli M, Monini P, Cafaro A, Ensoli F, Ensoli B. The presence of anti-Tat antibodies in HIV-infected individuals is associated with containment of CD4+ T-cell decay and viral load, and with delay of disease progression: results of a 3-year cohort study. Retrovirology. 11: 49. 2014.
22. Ensoli B, Fiorelli V, Ensoli F, Cafaro A, Titti F, Buttò S, et al. Candidate HIV-1 Tat vaccine development: from basic science to clinical trials. AIDS 2006; 20:2245–2261.
23. Ensoli B, Cafaro A, Monini P, Marcotullio S, Ensoli F. Challenges in HIV Vaccine Research for Treatment and Prevention. Front Immunol 2014; 5:417.
24. Cafaro A, Tripiciano A, Sgadari C, Bellino S, Picconi O, Longo O, et al. Development of a novel AIDS vaccine: the HIV-1 transactivator of transcription protein vaccine. Expert Opin Biol Ther 2015;15 Suppl 1:S13-29.
25. Bellino S, Francavilla V, Longo O, Tripiciano A, Paniccia G, Arancio A, et al. Parallel conduction of the phase I preventive and therapeutic trials based on the Tat vaccine candidate. Reviews on Recent Clinical Trials 2009; 4:195-204.
26. Ensoli B, Fiorelli V, Ensoli F, Lazzarin A, Visintini R, Narciso P, et al. The therapeutic phase I trial of the recombinant native HIV-1 Tat protein. AIDS 2008; 22: 2207-2209.
27. Ensoli B, Fiorelli V, Ensoli F, Lazzarin A, Visintini R, Narciso P, et al. The preventive phase I trial with the HIV-1 Tat based vaccine. Vaccine 2009; 28: 371-378.
28. Longo O, Tripiciano A, Fiorelli V, Bellino S, Scoglio A, Collacchi B, et al. Phase I therapeutic trial of the HIV-1 Tat protein and long term follow-up. Vaccine 2009; 27: 3306-3312.
29. Ensoli B, Bellino S, Tripiciano A, Longo O, Francavilla V, Marcotullio S, et al. Therapeutic immunization with HIV-1 Tat reduces immune activation and loss of regulatory T-cells and improves immune function in subjects on HAART. PLoS ONE 2010; 5: e13540

30. Ensoli F, Cafaro A, Casabianca A, Tripiciano A, Bellino S, Longo O, et al. HIV-1 Tat immunization restores immune homeostasis and attacks the HAART-resistant blood HIV DNA: results of a randomized phase II exploratory clinical trial. Retrovirology 2015;12:33.

16 APPENDIX I: SCHEDULE OF PROTOCOL VISITS & PROCEDURES

Week	0	24	54
Day	0	168	378
Study Visit	1	2	3
Informed consent	x		
Informed consent Addendum II			x
Medical history [1]	x[1]		
Eligibility verification	x		
Physical examination	x	x	x
Vital signs	x	x	x
Anti-tat antibodies (IgG, IgM, IgA)	x	x	x
CD4	x	x	x
HIV-1 plasma viraemia	x	x	x
Blood sample collection & storage for immunological & virological analyses	x	x	x
Counselling[2]	x	x	x
Serious adverse events	x	x	x
Concomitant medication	x	x	x
Telephonic contact with subject[3]			

1. Since the last visit of the ISS T-003 study.
2. Counselling: ARV adherence, risk reduction
3. Site to contact subject telephonically to give results of the following tests done during Visit 2/3: CD4+ T cell counts, HIV-1 plasma viraemia. Participant will be requested to come to the site if there is a need for a referral to a health facility.

17 APPENDIX II: DECLARATION OF HELSINKI

WORLD MEDICAL ASSOCIATION DECLARATION OF HELSINKI
Ethical Principles for Medical Research Involving Human Subjects
Adopted by the 18th WMA General Assembly, Helsinki, Finland, June 1964, and amended by the:
29th WMA General Assembly, Tokyo, Japan, October 1975
35th WMA General Assembly, Venice, Italy, October 1983
41st WMA General Assembly, Hong Kong, September 1989
48th WMA General Assembly, Somerset West, Republic of South Africa, October 1996
52nd WMA General Assembly, Edinburgh, Scotland, October 2000
53th WMA General Assembly, Washington 2002 (Note of Clarification on paragraph 29 added)
55th WMA General Assembly, Tokyo 2004 (Note of Clarification on Paragraph 30 added)
59th WMA General Assembly, Seoul, October 2008
64th WMA General Assembly, Fortaleza, Brazil, October 2013

A. INTRODUCTION
1. The World Medical Association (WMA) has developed the Declaration of Helsinki as a statement of ethical principles for medical research involving human subjects, including research on identifiable human material and data. The Declaration is intended to be read as a whole and each of its constituent paragraphs should not be applied without consideration of all other relevant paragraphs.
2. Although the Declaration is addressed primarily to physicians, the WMA encourages other participants in medical research involving human subjects to adopt these principles.
3. It is the duty of the physician to promote and safeguard the health of patients, including those who are involved in medical research. The physician's knowledge and conscience are dedicated to the fulfilment of this duty.
4. The Declaration of Geneva of the WMA binds the physician with the words, “The health of my patient will be my first consideration,” and the International Code of Medical Ethics declares that, “A physician shall act in the patient's best interest when providing medical care.”
5. Medical progress is based on research that ultimately must include studies involving human subjects. Populations that are underrepresented in medical research should be provided appropriate access to participation in research.
6. In medical research involving human subjects, the well-being of the individual research subject must take precedence over all other interests.
7. The primary purpose of medical research involving human subjects is to understand the causes, development and effects of diseases and improve preventive, diagnostic and therapeutic interventions (methods, procedures and treatments). Even the best current interventions must be evaluated continually through research for their safety, effectiveness, efficiency, accessibility and quality.
8. In medical practice and in medical research, most interventions involve risks and burdens.
9. Medical research is subject to ethical standards that promote respect for all human subjects and protect their health and rights. Some research populations are particularly vulnerable and need special protection. These include those who cannot give or refuse consent for themselves and those who may be vulnerable to coercion or undue influence.
10. Physicians should consider the ethical, legal and regulatory norms and standards for research involving human subjects in their own countries as well as applicable international norms and standards. No national or international ethical, legal or regulatory requirement should reduce or eliminate any of the protections for research subjects set forth in this Declaration.

B. PRINCIPLES FOR ALL MEDICAL RESEARCH
11. It is the duty of physicians who participate in medical research to protect the life, health, dignity, integrity, right to self-determination, privacy, and confidentiality of personal information of research subjects.
12. Medical research involving human subjects must conform to generally accepted scientific principles, be based on a thorough knowledge of the scientific literature, other relevant sources of information, and adequate laboratory and, as appropriate, animal experimentation. The welfare of animals used for research must be respected.
13. Appropriate caution must be exercised in the conduct of medical research that may harm the environment.
14. The design and performance of each research study involving human subjects must be clearly described in a research protocol. The protocol should contain a statement of the ethical considerations involved and should indicate how the principles in this Declaration have been addressed. The protocol should include information regarding funding, sponsors, institutional affiliations, other potential conflicts of interest, incentives for subjects

and provisions for treating and/or compensating subjects who are harmed as a consequence of participation in the research study. The protocol should describe arrangements for post-study access by study subjects to interventions identified as beneficial in the study or access to other appropriate care or benefits.
15. The research protocol must be submitted for consideration, comment, guidance and approval to a research ethics committee before the study begins. This committee must be independent of the researcher, the sponsor and any other undue influence. It must take into consideration the laws and regulations of the country or countries in which the research is to be performed as well as applicable international norms and standards but these must not be allowed to reduce or eliminate any of the protections for research subjects set forth in this Declaration. The committee must have the right to monitor ongoing studies. The researcher must provide monitoring information to the committee, especially information about any serious adverse events. No change to the protocol may be made without consideration and approval by the committee.
16. Medical research involving human subjects must be conducted only by individuals with the appropriate scientific training and qualifications. Research on patients or healthy volunteers requires the supervision of a competent and appropriately qualified physician or other health care professional. The responsibility for the protection of research subjects must always rest with the physician or other health care professional and never the research subjects, even though they have given consent.
17. Medical research involving a disadvantaged or vulnerable population or community is only justified if the research is responsive to the health needs and priorities of this population or community and if there is a reasonable likelihood that this population or community stands to benefit from the results of the research.
18. Every medical research study involving human subjects must be preceded by careful assessment of predictable risks and burdens to the individuals and communities involved in the research in comparison with foreseeable benefits to them and to other individuals or communities affected by the condition under investigation.
19. Every clinical trial must be registered in a publicly accessible database before recruitment of the first subject.
20. Physicians may not participate in a research study involving human subjects unless they are confident that the risks involved have been adequately assessed and can be satisfactorily managed. Physicians must immediately stop a study when the risks are found to outweigh the potential benefits or when there is conclusive proof of positive and beneficial results.
21. Medical research involving human subjects may only be conducted if the importance of the objective outweighs the inherent risks and burdens to the research subjects.
22. Participation by competent individuals as subjects in medical research must be voluntary. Although it may be appropriate to consult family members or community leaders, no competent individual may be enrolled in a research study unless he or she freely agrees.
23. Every precaution must be taken to protect the privacy of research subjects and the confidentiality of their personal information and to minimize the impact of the study on their physical, mental and social integrity.
24. In medical research involving competent human subjects, each potential subject must be adequately informed of the aims, methods, sources of funding, any possible conflicts of interest, institutional affiliations of the researcher, the anticipated benefits and potential risks of the study and the discomfort it may entail, and any other relevant aspects of the study. The potential subject must be informed of the right to refuse to participate in the study or to withdraw consent to participate at any time without reprisal. Special attention should be given to the specific information needs of individual potential subjects as well as to the methods used to deliver the information. After ensuring that the potential subject has understood the information, the physician or another appropriately qualified individual must then seek the potential subject's freely-given informed consent, preferably in writing. If the consent cannot be expressed in writing, the non-written consent must be formally documented and witnessed.
25. For medical research using identifiable human material or data, physicians must normally seek consent for the collection, analysis, storage and/or reuse. There may be situations where consent would be impossible or impractical to obtain for such research or would pose a threat to the validity of the research. In such situations the research may be done only after consideration and approval of a research ethics committee.
26. When seeking informed consent for participation in a research study the physician should be particularly cautious if the potential subject is in a dependent relationship with the physician or may consent under duress. In such situations the informed consent should be sought by an appropriately qualified individual who is completely independent of this relationship.
27. For a potential research subject who is incompetent, the physician must seek informed consent from the legally authorized representative. These individuals must not be included in a research study that has no likelihood of benefit for them unless it is intended to promote the health of the population represented by the potential subject, the research cannot instead be performed with competent persons, and the research entails only minimal risk and minimal burden.

28. When a potential research subject who is deemed incompetent is able to give assent to decisions about participation in research, the physician must seek that assent in addition to the consent of the legally authorized representative. The potential subject's dissent should be respected.
29. Research involving subjects who are physically or mentally incapable of giving consent, for example, unconscious patients, may be done only if the physical or mental condition that prevents giving informed consent is a necessary characteristic of the research population. In such circumstances the physician should seek informed consent from the legally authorized representative. If no such representative is available and if the research cannot be delayed, the study may proceed without informed consent provided that the specific reasons for involving subjects with a condition that renders them unable to give informed consent have been stated in the research protocol and the study has been approved by a research ethics committee. Consent to remain in the research should be obtained as soon as possible from the subject or a legally authorized representative.
30. Authors, editors and publishers all have ethical obligations with regard to the publication of the results of research. Authors have a duty to make publicly available the results of their research on human subjects and are accountable for the completeness and accuracy of their reports. They should adhere to accepted guidelines for ethical reporting. Negative and inconclusive as well as positive results should be published or otherwise made publicly available. Sources of funding, institutional affiliations and conflicts of interest should be declared in the publication. Reports of research not in accordance with the principles of this Declaration should not be accepted for publication.

C. ADDITIONAL PRINCIPLES FOR MEDICAL RESEARCH COMBINED WITH MEDICAL CARE

31. The physician may combine medical research with medical care only to the extent that the research is justified by its potential preventive, diagnostic or therapeutic value and if the physician has good reason to believe that participation in the research study will not adversely affect the health of the patients who serve as research subjects.
32. For medical research using identifiable human material or data, such as research on material or data contained in biobanks or similar repositories, physicians must seek informed consent for its collection, storage and/or reuse. There may be exceptional situations where consent would be impossible or impracticable to obtain for such research. In such situations the research may be done only after consideration and approval of a research ethics committee.
33. The benefits, risks, burdens and effectiveness of a new intervention must be tested against those of the best current proven intervention, except in the following circumstances:
• The use of placebo, or no treatment, is acceptable in studies where no current proven intervention exists; or
• Where for compelling and scientifically sound methodological reasons the use of placebo is necessary to determine the efficacy or safety of an intervention and the patients who receive placebo or no treatment will not be subject to any risk of serious or irreversible harm. Extreme care must be taken to avoid abuse of this option.

Post-Trial Provisions

34. In advance of a clinical trial, sponsors, researchers and host country governments should make provisions for post-trial access for all participants who still need an intervention identified as beneficial in the trial. This information must also be disclosed to participants during the informed consent process.

Research Registration and Publication and Dissemination of Results

35. Every research study involving human subjects must be registered in a publicly accessible database before recruitment of the first subject.
36. Researchers, authors, sponsors, editors and publishers all have ethical obligations with regard to the publication and dissemination of the results of research. Researchers have a duty to make publicly available the results of their research on human subjects and are accountable for the completeness and accuracy of their reports. All parties should adhere to accepted guidelines for ethical reporting. Negative and inconclusive as well as positive results must be published or otherwise made publicly available. Sources of funding, institutional affiliations and conflicts of interest must be declared in the publication. Reports of research not in accordance with the principles of this Declaration should not be accepted for publication.

Unproven Interventions in Clinical Practice

37. In the treatment of an individual patient, where proven interventions do not exist or other known interventions have been ineffective, the physician, after seeking expert advice, with informed consent from the patient or a legally authorised representative, may use an unproven intervention if in the physician's judgement it offers hope of saving life, re-establishing health or alleviating suffering. This intervention should subsequently be made the object of research, designed to evaluate its safety and efficacy. In all cases, new information must be recorded and, where appropriate, made publicly available

18 APPENDIX IV: GLOSSARY

ADCC	Antibody-mediated Cellular Cytotoxicity
AIDS	Acquired Immune Deficiency Syndrome
ANOVA	Analysis of Variance
CCR-5	Chemokine Receptor 5
CD	Cluster of Differentiation
CFSE	CarboxyFluoroscein Succinimidyl Ester
CRF	Case Report Form
CRO	Contract Research Organization
CTLs	Cytotoxic T Lymphocytes
dl	Deciliter
DNA	Deoxyribonucleic Acid
ELISA	Enzyme-Linked Immunosorbent Assay
EudraCT	European Clinical Trials DatabaseEuropea
g	Gram
GCP	Good Clinical Practice
GMT	Geometric Mean Titers
HAART	Highly Active Antiretroviral Therapy
HCT	Hematocrit
HCV	Hepatitis C Virus
HIV-1	Human Immunodeficiency Virus type 1
HIV-2	Human Immunodeficiency Virus type 2
HLA	Human Leukocyte Antigen
ICH	International Conference on Harmonization
ICS	Intracellular Cytokine Staining
ID	Identification number
id	Intradermally
IFO	Istituti Fisioterapici Ospitalieri
IgA	Immunoglobulin A
IgG	Immunoglobulin G
IgM	Immunoglobulin M
IL-2	Interleukin-2
IL-4	Interleukin-4
ISS	Istituto Superiore di Sanità
Kg	Kilogram
MDDCs	Monocyte-derived dendritic cells
MedDRA	Medical Dictionary for Regulatory Activities
mg	Milligram
min	Minute
ml	Millilitre
NIH	National Institutes of Health
NK	Natural Killer
PBMC	Peripheral Blood Mononuclear Cell
RBC	Red blood cell
RNA	Ribonucleic Acid
SAE	Serious Adverse Event
SAS	Statistical and data Analysis Software
SHIV	Simian/Human Immunodeficiency Virus
SIV	Simian Immunodeficiency Virus

SUSAR	Suspected Unexpected Serious Adverse Reaction
Th1	Type 1 helper T Cells
Th2	Type 2 helper T Cells
TM	Trade Mark
TNF-α	Tumor Necrosis Factor-α
WBC	White Blood Cell
WHO	World Health Organization
WMA	World Medical Association
γIFN	γInterferon
μg	Microgram
μl	Microliter
°C	Temperature in degrees Celsius
