# Supplementary material for: Therapeutic HIV-1 Tat vaccination promotes durable immune reconstitution and reservoir reduction in ART-treated adults with clade C infection: a 12-year follow-up study
Source: Front Immunol. 2026 Apr 10;17:1769223. doi: 10.3389/fimmu.2026.1769223 (PMC13106519; doi:10.3389/fimmu.2026.1769223)
Supplement: Supplementary file 1 [file DataSheet1.zip › Data Sheet1/Appendix 1/ISS T-003 Protocol.pdf]

SPONSOR
AIDS National Center
Istituto Superiore di Sanità (ISS)
Viale Regina Elena, 299
00161 Rome, Italy

CLINICAL SITE
Medunsa Clinical Research Unit
Medunsa Campus
University of Limpopo
Gauteng Province
South Africa

PRINCIPAL INVESTIGATOR
Prof. Maphoshane Nchabeleng

A Phase II, Randomized, Double-Blind, Placebo-Controlled Trial to evaluate the Immunogenicity and Safety of a Therapeutic, Recombinant, Biologically Active HIV-1 Tat Protein Vaccine in HIV-Infected, Anti-Tat Negative, ARV-Treated Adult Volunteers

Protocol Number: ISS T-003

Protocol Version: ISS T003 Protocol Version 4.0 20-Feb-2013

Confidentiality Statement

The information provided in this document is strictly confidential and is available for review to investigators, potential investigators, appropriate ethics committees and other national authorities. No disclosure should take place without the written authorization from *Istituto Superiore di Sanità*, except to the extent necessary to obtain informed consent from potential subjects.

CLINICAL SITE AND PRINCIPAL INVESTIGATOR

Medunsa Clinical Research Unit (MeCRU)
Medunsa Campus
University of Limpopo
Gauteng Province
South Africa
Principal Investigator: Prof. Maphoshane Nchabeleng

LIST OF OTHER PARTICIPANTS

Sponsor

AIDS National Center
Istituto Superiore di Sanità (ISS)
Viale Regina Elena, 299
00161 Rome, Italy
Tel. +39 06 49903209
Fax +39 06 49903002
Sponsor Responsible – Dr. Barbara Ensoli

Core Laboratory of Immunology and Virology

Ospedale S. Gallicano
Istituti Fisioterapici Ospitalieri
Via E. Chianesi, 53
00144 Rome, Italy
Tel. +39 06 52662780/2782
Fax +39 06 52662781
Laboratory Responsible – Dr. Fabrizio Ensoli

National Health Laboratory Service (NHLS)

Northern Branch
Dr George Mukhari Hospital, Ga-Rankuwa, Pretoria
Tel: +27 (0)12 521 4282
Fax: +27 (0)12 521 4281

Contract Research Organization

Triclinium Clinical Trial Project Management (Pty) Ltd.
135 West Street
Sandown 2196
South Africa
Tel: +27 11 883 0206
Fax: +27 11 784 2818

EMERGENCY CONTACTS

Role in the study	Name	Telephone/Fax/e-mail
Sponsor<br>AIDS National Center<br>Istituto Superiore di Sanità<br>Rome, Italy.<br>(SA Head Office<br>c/o MRC-SAAVI - Cape Town)	Dr. Paolo Monini	Tel. +27 21 938 0205<br>Fax +27 21 938 0302<br>email: paolo.monini@iss.it
Principal Investigator<br>Medunsa Clinical Research Unit	Dr. Maphoshane Nchabeleng	Tel: +27 12 521 5667<br>Fax: +27 12 521 5727<br>email: nchabe@ul.ac.za
CRO<br>Triclinium Clinical Trial Project Management (Pty) Ltd.	Dr Havana Chikoto<br>(Project Manager)<br>Ms. Mary-Anne Bopape<br>(Safety Reporting)	Tel: +27 11 883 0206<br>Fax: +27 11 7842 818<br>email: havana.chikoto@triclinium.net<br>email: mary-anne.bopape@triclinium.net

TABLE OF CONTENTS

1 PROTOCOL SYNOPSIS 7
2 INTRODUCTION AND RATIONALE 9

2.1 Background 9

2.2 Rationale for the Selection of Vaccine Dosage and Schedule of Vaccination 12
3 STUDY OBJECTIVES 13
4 STUDY DESIGN 13
5 INVESTIGATIONAL PRODUCT AND DOSAGE REGIMENS 13

5.1 Investigational product: Background on Investigational product preparation 13
5.1.1 Investigational Product Preparation 14
5.1.2 Vaccination schedule 14

5.2 Study Duration 14

5.3 Investigational Product Import and Delivery 15

5.4 Packaging and Labelling of Investigational product 15

5.5 Investigational Product Storage and Accountability 16

5.6 Randomization Codes 16
6 METHODS 17

6.1 Inclusion Criteria 17

6.2 Exclusion Criteria 17

6.3 Screening Failures 18

6.4 Randomization 18

6.5 Blinding/Un-blinding 19

6.6 Study Assessments 19
6.6.1 Clinical Assessments 19
6.6.2 Standard Laboratory Assessments 19
6.6.3 Immunological Evaluations 20
6.6.4 Virology Evaluations 21
6.6.5 Sample Collection, Storage and Shipment 22

6.7 Schedule of Study Procedures 23

6.8 Compliance 28
7 PREVIOUS AND CONCOMITANT MEDICATION 28

7.1 Antiretroviral Therapy 28

7.2 Prohibited Medication/Therapy 28

8 ENDPOINTS 29

8.1 Primary Endpoint Variable and Measurements 29

8.2 Secondary Endpoint Variables and Measurements 29
9 SAFETY 30

9.1 Responsibility to Ensure the Safety of Trial Subjects 30
9.1.1 Principal Investigator 30
9.1.2 Study Sponsor 30

9.2 Safety Surveillance during the Study 31
9.2.1 Adverse Event (AE) 31
9.2.2 Serious Adverse Event (SAE) 32
9.2.3 Adverse Drug Reaction (ADR) 32
9.2.4 Serious Adverse Drug Reaction (SADR) 33

9.3 Reporting of Adverse Events/Adverse Drug Reactions 33
9.3.1 Assessing Severity 33
9.3.2 Assessing a Causal Relationship (Relatedness) 34

9.4 Adverse Event Treatment, Follow-up and Outcome 35

9.5 Reporting of Serious Adverse Events/Serious Adverse Drug Reactions 35

9.6 Other Events Requiring Immediate Reporting 36

9.7 Follow-up of Subjects Who Become Pregnant 37

9.8 Subject Diary and Temperature Monitoring 38
10 STUDY PAUSE/STOPPING RULES 38

10.1 General rules for a study pause 38

10.2 Premature Discontinuation of the Study 38
11 DATA RECORDING, MONITORING AND DATA MANAGEMENT 39

11.1 Data Recording 39

11.2 Data Monitoring 39

11.3 Data Management 40
12 STATISTICAL ANALYSIS 40

12.1 Statistical Program 40

12.2 Endpoints 40

12.3 Sample Size Determination 41

12.4 Population for Statistical Evaluation 41

12.5 Methods **41**

12.6 Primary Endpoint Analysis **41**

12.7 Secondary Endpoint Analysis **41**

12.8 Interim analysis **42**

12.8.1 Rationale/reason for interim analysis 42
12.8.2 Statistical analysis and population 42
12.8.3 Preserving of blindness 42
12.8.4 Timing of interim analysis 42
12.8.5 Adjustments to Type 1 error 43

13 ETHICAL AND ADMINISTRATIVE PROCEDURES **43**

13.1 Informed Consent **43**

13.2 Ethical Issues and the Independent Ethics Committee **44**

13.3 Subjects Data Protection – Direct Access to Source Data **44**

13.4 Insurance, Indemnity and Refunds **45**

13.5 Audits and Inspections **45**

13.6 Training of Study Staff **45**

14 CASE REPORT FORMS & RECORD RETENTION **45**

15 PUBLICATION OF RESULTS **46**

16 STUDY TIMETABLE **46**

17 AMENDMENTS TO THE PROTOCOL **47**

17.1 Summary of changes from Protocol Version 1.0 to Version 2.0 .... **47**

17.2 Summary of changes from Protocol Version 2.0 to Version 3.0 .... **61**

18 INVESTIGATORS AGREEMENT **85**

19 REFERENCES **86**

20 APPENDIX I: TOXICITY TABLE **91**

21 APPENDIX II: SCHEDULE OF PROTOCOL VISITS & PROCEDURES **99**

22 APPENDIX III: DECLARATION OF HELSINKI **100**

23 APPENDIX IV: GLOSSARY **103**

1 PROTOCOL SYNOPSIS

Title:
A Phase II randomized, double-blind, placebo-controlled trial to evaluate the immunogenicity and safety of a therapeutic, recombinant, biologically active HIV-1 Tat protein vaccine in HIV-infected, anti-Tat negative, ARV-treated adult volunteers.

Sponsor:
Istituto Superiore di Sanita, AIDS National Center

Study Objectives:
Primary: To demonstrate that three 30μg doses of the Tat vaccine administered intradermally at 4 week intervals is immunogenic as compared to placebo in HIV-1 infected, anti-Tat antibody negative individuals on antiretroviral therapy.
Secondary: To monitor the safety of the Tat vaccine versus placebo in vaccinated volunteers.

Study Design:
A Phase II, randomized, double-blind, placebo-controlled clinical trial.

Planned Study Dates:
24 months. Study start is estimated to be October 2011.

Study Duration:
To evaluate eligibility, subjects will be screened for up to 35 days prior to treatment initiation. They will then be followed up for a 48 week period from the time of the first vaccination (i.e. for a total of 40 weeks from the last vaccination)

Study Population:
A total of 200 subjects will be randomized into 2 treatment arms in a 1:1 ratio:
Group A: 3 intradermal administrations of Tat (30 μg) vaccine
Group B: 3 intradermal administrations of placebo

Inclusion Criteria:
1. Male and female volunteers aged 18-45 years (inclusive)
2. Anti-Tat antibody negative
3. HIV-1 infected individuals currently receiving treatment with ARVs
4. Chronically suppressed HIV-1 infection as indicated by a HIV-1 plasma viraemia < 400 copies/ml and a CD4+ T cell count ≥ 200 cells/μl at screening, and documented at least once during the 12 month period prior to screening, irrespective of the pre-ARV CD4 nadir.
5. Negative pregnancy test for females of childbearing potential (not sterilized and still menstruating or within 1 year of the last menses) to be performed during the screening phase and immediately before each vaccination, and use of an acceptable method of contraception (double barrier methods, combined oral contraceptives, injectable contraceptives or intra-uterine devices) for at least 3 weeks prior to the first vaccination and for the duration of the study.
6. Has provided written informed consent.
7. Agrees to stay in contact with the research site for the duration of the study

Exclusion Criteria:
1. Acute illness on Study Day 0
2. Body temperature ≥37.5°C on Study Day 0
3. Any current AIDS-related opportunistic disease
4. Any current neoplastic disease *[NOTE: Criterion is meant to exclude volunteers with active invasive cancer. Volunteers who present with intraepithelial neoplasia during the course of the study are to continue with all study procedures.]*
5. Known history of malignant neoplastic diseases [*NOTE: Volunteers with known history of non-malignant neoplastic diseases that are completely resolved according to the fulfilment of all the specific recovery criteria, in agreement with the current guidelines in medical oncology, are eligible*]
6. Known history of encephalopathy, neuropathy or unstable CNS pathology,

immunodeficiency, autoimmune disease, angina or cardiac arrhythmias, or any other clinically significant medical problems in the opinion of the investigator

7. Any evidence, as judged by the investigator, of unstable cardio-vascular disease (e.g. unstable hypertensive disease needing modification or introduction of an anti-hypertensive treatment)
8. Chest radiography showing evidence of active or acute cardiac or pulmonary disease within 6 months prior to the study screening visit [Note if no previous chest X-ray available, this will be performed at screening];
9. Known history of anaphylaxis or serious adverse reactions to vaccines
10. Known history of serious allergic reaction to any substance, requiring hospitalization or emergent medical care (e.g. Steven-Johnson syndrome, bronchospasm, or hypotension)
11. Active pulmonary tuberculosis within 12 months of screening as evidenced by chest radiography and/or medical history.
12. Any known medical or psychiatric condition which precludes subject compliance with the protocol, specifically, persons with psychotic disorders, major affective disorders and/or suicidal ideation are to be excluded
13. Current use of psychotropic drugs prescribed for major psychotic disorders
14. Concomitant participation in any study with an investigational product or device
15. Current or prior therapy with immunomodulator, immunosuppressive and/or anticoagulant drugs within 30 days prior to administration of the investigational product
16. Live attenuated vaccines within 60 days of the first administration of the investigational product [*NOTE: Medically indicated sub-unit or killed vaccines (e.g., influenza, pneumococcal, hepatitis A and B) are permitted, but must have been administered at least 4 weeks prior to the first administration of the investigational product*]
17. Known receipt of blood products or immunoglobulins during the year prior to screening
18. Previous participation in an HIV-1 vaccine trial (volunteers who are known to have previously participated in the placebo arm of an HIV-1 vaccine trial, and so have never received an investigational HIV-1 vaccine, are eligible for inclusion)
19. Known drug and/or alcohol abuse in the year prior to screening
20. Use in the last 6 months or concomitant use of anti-CCR5 inhibitors and/or integrase inhibitors and/or fusion inhibitors
21. Pregnant or lactating females

Investigational Product, Administration And Dose Regime:
Biologically active, recombinant HIV-1 Tat protein vaccine (30 µg) administered intradermally at weeks 0, 4 and 8.

Interim Analyses
At least 50% of the original planned population (at least 100 subjects) will be included in an interim analysis. Data derived from the interim analysis will be used to re-calculate the sample size.

Statistical Analyses:
All statistical analyses will be performed using SAS® version 9.2 or higher. Two subject populations will be considered for statistical analysis:

1. The immunogenicity population, representing all randomized subjects who received at least 2 vaccinations.
2. The safety population, representing all randomized subjects who received at least one administration of Tat.

Primary Immunogenicity Variable
Induction of anti-Tat antibodies (IgM, IgG or IgA) as specific anti-Tat humoral immune response.

Secondary Variables
Adverse events, including any significant changes in hematological/biochemical/coagulation and urinalysis laboratory parameters.
Final analysis will be performed after all subjects have completed the study, the database has been locked, and the study has been unblinded.

2 INTRODUCTION AND RATIONALE

This Phase II study is directed at evaluating the immunogenicity (as a primary end-point) and the safety (as a secondary end-point), of a therapeutic, recombinant, biologically active HIV-1 Tat vaccine in HIV-1 infected, anti-Tat antibody negative, ARV-treated adult volunteers with chronically suppressed HIV-1 infection as indicated by a HIV-1 plasma viraemia < 400 copies/ml and a CD4+ T cell count ≥ 200 cells/μl, at screening and documented at least once during the 12 month period prior to screening, irrespective of the pre-ARV CD4 nadir.
This vaccine strategy has been evaluated pre-clinically in cynomolgus monkeys, in Phase I preventive and therapeutic clinical trials as described below and is currently being tested in a Phase II trial in ARV-treated patients in Italy.

2.1 Background

Over the last 20 years most of the efforts in HIV vaccine development have been focused on achieving sterilizing immunity by targeting the Envelope protein (Env) of HIV, that is responsible for the binding and entry of the virus, with the rationale of generating neutralizing antibodies (NA) capable of protecting from infection (Wahren, 2002). Alternatively, more recent approaches have been attempted, by combining multiple HIV antigens with the rationale of generating strong antiviral cellular immune responses capable of preventing infection and/or reducing virus replication and progression to disease. However, results from clinical trials, including the first phase III trial (AIDSVAX by VaxGen) and the first phase II “Proof-of-Concept” trial (HVTN 502/Merck 023) have been largely disappointing since no protection from primary infection and no effect on viral load have been observed. The inability of such vaccines to elicit protective immune responses can be, at least in part, explained by the high Env variability that hampers recognition of relevant epitopes by NA, and by the heavy glycosylation of gp120 that contributes in hiding critical (neutralizing) Env-epitopes (reviewed in Burton, 1997). On the other hand, the induction of strong cellular responses against a combination of different HIV antigens (Gag, Pol and Nef) failed both in preventing and in controlling infection and virus replication (news release Merck/HVTN, 2007) suggesting that a more balanced induction of humoral and cellular immune responses might be necessary to increase the chance of success of the candidate vaccines. New and alternative vaccine strategies have been therefore developed aimed at blocking disease onset by targeting viral regulatory genes, which are essential for virus replication and infectivity. Control of infection, which is achieved in the absence of sterilizing immunity, should provide protection from disease progression and reduce virus transmission to healthy individuals. Thus, this approach may be effective for both preventive and therapeutic vaccine strategies.

Being a very early regulatory protein and playing a major role in HIV-1 replication and pathogenesis, Tat represents an optimal candidate for such vaccine strategies (Ensoli, 1990, 1993 and 1994; Chang, 1997; Ensoli, 2006). Tat is a key viral regulatory protein produced very early after infection, even prior to HIV integration, and necessary for viral gene expression (Arya, 1985; Fisher, 1986; Ensoli, 1993; Wu, 2001), cell-to-cell virus transmission and disease progression. In fact, in the absence of Tat no or negligible amounts of structural proteins are expressed and, therefore, no infectious virus is made. Further, Tat is released by the infected T lymphocytes in the extracellular milieu (Ensoli, 1990 and 1993; Chang, 1997) and enters both infected cells, in which promotes HIV-1 replication, while exerting multiple effects on uninfected cells, which facilitate, directly or indirectly, cell recruitment and activation, thus providing new cell targets for HIV tissue propagation and

systemic spreading of the infection (Ensoli, 1990 and 1993; Chang, 1997; Shutt and Soll, 1999; Koedel, 1999; Arora, 2002; Caputo, 2004; James, 2004; Ferrantelli, 2004).
Several studies suggest that an immune response to Tat has a protective role and may control the progression of the disease *in vivo* (Reiss, 1990; Rodman, 1993; Re, 1995; Zagury, 1998; Re, 2001). In particular, a higher prevalence of anti-Tat antibodies has been shown in asymptomatic HIV-infected individuals as compared to patients in advanced stages of the disease (Krone, 1988; Demirhan, 2000; Re, 2001) and in non-progressors as compared to fast progressors (Zagury, 1998).

A cross-sectional and longitudinal study has been performed in a cohort of 252 individuals with known dates of seroconversion and a medium follow-up of 7.2 years (Rezza, 2005). The risk of developing AIDS or severe immunodeficiency was 60% lower for anti-Tat positive individuals as compared to anti-Tat negative individuals. A longitudinal analysis performed on 139 individuals (with at least two serum samples) indicated no progression to disease in persistently anti-Tat positive individuals (n = 10). Even individuals with a transient anti-Tat positive determination had a slower progression, as compared to persistently anti-Tat negative individuals. In particular, none of the persistently anti-Tat positive individuals developed AIDS, whereas AIDS or severe immunodeficiency occurred in 53 individuals among those who were anti-Tat negative. These results indicate that presence of anti-Tat antibodies is predictive of a slower progression to AIDS and/or severe immunodeficiency (Rezza, 2005).

Moreover Tat is conserved in its immunogenic regions (both B and T cell) among all subtypes. Recent data, in fact, indicate an effective cross-clade recognition of clade B strain-derived (BH-10) Tat protein from the HTLV-IIIB lab-adapted virus strain (Buttò, 2003), which was isolated about 20 years ago (Ratner, 1985), by sera from individuals infected with viruses circulating at the present in Italy and in Africa, thus reflecting the high degree of conservation of the corresponding Tat regions. Specifically, sera from Italian, Ugandan and South African patients who are mainly infected with A, B, C and D and to a lesser extent, with F and G HIV-1 subtypes, recognize the BH-10 Tat protein at similar levels (e.g. prevalence and titers of anti-Tat antibodies) (Buttò, 2003). This observation is reinforced by the results of sequence conservation analysis, demonstrating that the predicted amino acidic sequence of Tat is well conserved among the different circulating viruses belonging to distinct HIV-1 clades and presents a relatively high degree of homology with the BH-10 Tat sequence (Buttò, 2003). These findings indicate that the overall identity of Tat is preserved and provide strong formal evidence that a Tat-based vaccine may indeed be used in the different geographic areas of the world, since it is capable of inducing a broad immune response against different virus clades.

The Tat vaccine has also the advantage of maintaining the vaccinees HIV-negative according to the current serological tests for diagnosis of HIV infection, since it does not contain structural HIV proteins on which these tests are based. This greatly facilitates recruitment and trial participation as well as vaccinees monitoring by avoiding vaccination-induced seroconversion.

Preclinical studies performed in different animal models, including mice and cynomolgus monkeys, demonstrated that vaccination with a biologically active Tat protein or tat DNA is safe, elicits a broad and specific immune response and, most importantly, induces a long-term protection against infection with a highly pathogenic virus (SHIV 89.6P), which

rapidly causes AIDS and death in these monkeys (Cafaro, 1999, 2000, 2001 and Cafaro J. Virol, 2010).

Based on these results Phase I preventive and therapeutic clinical trials (Clinicaltrials.gov identifier NCT00529698 and NCT00505401 respectively) have been sponsored by ISS and conducted in 4 clinical centres in Italy. The results of the phase I clinical trials indicate that the Tat vaccine is safe and immunogenic in both uninfected and infected individuals (Ensoli et al AIDS 2008, Vaccine 2009; Longo et al Vaccine 2009; Bellino et al Rev Rec Clin Trials 2009). Safety was evaluated by monitoring the subjects for local and systemic adverse reactions during the course of the trials. Clinical evaluation of safety also included monitoring of haematological (including coagulation assessment), biochemical (including liver and kidney functional parameters) and immunological (including CD4, CD8, CD3 T cells, NK, B cells and monocytes) parameters. In the therapeutic setting, the safety profile assessment also included the evaluation of plasma viraemia levels.

Assessment of safety was performed at baseline and at several time points during the study. Overall, no clinically significant alterations in laboratory values have been identified in any treatment group. AEs were coded according to the MedDRA dictionary and grouped in accordance to the MedDRA System Organ Classes (SOC). An independent, “ad hoc” safety monitoring board periodically evaluated all safety documentation including the frequency and characteristics of AEs. Safety monitoring confirmed that the vaccine based on the recombinant Tat protein is safe and well tolerated. The most frequent AEs were mild and did not appear to be clearly dose related, including injection site reactions, asthenia, fever, headache and transient blood disorders (mainly leukocytosis and neutrophilia).

Vaccination efficiently induced both humoral and cellular immune responses against the Tat protein (Ensoli et al AIDS 2008 and Vaccine 2009, Longo et al Vaccine 2009, Bellino et al Rev Rec Clin Trials 2009). In particular, in HIV-infected asymptomatic individuals, intradermal vaccination with Tat (given 5 times on a monthly schedule), in the absence of adjuvant, not only induced functional antibodies, but also partially reverted the marked Th1 polarization of anti-Tat immunity seen in natural infection eliciting a more balanced Th1/Th2 immune response (Ensoli et al AIDS 2008, Longo et al Vaccine 2009). Remarkably, the number of CD4+ T cells had a significant positive correlation with anti-Tat antibody titers, which persisted up to 144 weeks after the first vaccination (Ensoli et al Vaccine 2009, Longo et al Vaccine 2009, Bellino et al Rev Rec Clin Trials 2009).

Based on these data, a phase II therapeutic, open label, clinical study with Tat protein (ISS T-002, ClinicalTrials.gov NCT00751595) was sponsored by ISS and activated in 10 clinical sites in Italy in HIV-infected HAART-treated subjects negative for anti-Tat antibodies at baseline, with undetectable viral load and no evidence of virological rebounds (viral <50 copies/ml in the last 6 months before enrolment), with CD4+ T cell counts >400 cells/µl and a CD4 nadir >250 cells/µl. In this study, subjects are randomized into two arms to receive 3 or 5 vaccinations monthly; each arm is composed of two treatment groups, receiving 7, 5 or 30 µg of Tat, respectively.
Preliminary results obtained from 87 subjects enrolled in the phase II trial ISS T-002 ongoing in Italy, indicate that Tat vaccination is safe (as evaluated by the DSMB), immunogenic and capable of reducing the immune dysregulation which persists despite HAART in treated individuals, opening new avenues for a most effective treatment of HIV/AIDS (Ensoli et al, PLoS ONE 2010). In fact, in spite of effective viral suppression, HAART does not lead to a complete normalization of immune activation and immune

dysfunction parameters (Battegay et al, 2006; Byrnes et al, 2008; Glencross DK et al, 2008; Hazenberg MD et al, 2003; Hunt PW et al, 2003; Hunt PW, 2007; Kelley CF et al, 2009; Smith DE et al, 2004; Valdez H et al, 2002; Chehimi J et al, 2007). By contrast, Tat vaccination effectively reduced immune activation, as indicated by the significant decrease of CD25+/CD4+ and CD38+/CD8+ T cells and the corresponding reduction of the serum levels of total immunoglobulins, neopterin and β2-microglobulin, which was correlated with the anti-Tat antibody titres and were observed together to a significant increase of regulatory T-cells (CD4+/CD25+/FOXP3+ T cells). Furthermore, a significant increase of CD4+ T cell, B lymphocytes, and of the peripheral blood cell viability as well as the frequency and intensity of CD4+ and CD8+ T cell responses against Env and recall antigens were also observed in vaccinated individuals as compared to a reference group of 32 subjects, with the same inclusion criteria as the T-002 study, which were followed in an observational study (ISS OBS T-002) conducted in parallel at the same clinical centres. Of note, the maximal therapeutic effects were observed in subjects vaccinated with 30 µg of Tat, particularly in the most immune-compromised individuals. Furthermore these results have been confirmed up to 2 years after the first immunization.
Based on these results, and in view of the urgency to improve HIV treatment particularly in individuals with a more advanced immune deterioration, the Investigators and the Data Safety Monitoring Board (DSMB) endorsed an amendment that has been approved by the Ethical Committees. This amendment extended the enrolment criteria to include more immune compromised individuals (CD4+ T cell counts ≥ 200 cells/µl, with any CD4 nadir) and expanded the total sample size from 128 to 160 volunteers. The enrolment was recently completed and follow-up of the subjects is continuing.

2.2 Rationale for the Selection of Vaccine Dosage and Schedule of Vaccination

Although the Tat vaccine was shown to be safe and highly immunogenic at all doses, more intense humoral immune responses were observed with 7.5 and 30 µg doses of Tat as compared to the 15 µg dose and more persistent responses were induced by the 30 µg dose. Further, Tat-specific cellular responses, particularly IL-4 and lymphoproliferation, were more frequent in subjects randomized to intradermal administration.

With regard to the vaccination schedule, in the Phase I trials the anti-Tat antibody titres peaked after three vaccinations. In the ongoing phase II clinical trial, the administration of three intradermal vaccinations with 30 µg of Tat were associated with a significant increase of CD4+ T cell and B cell numbers, as well as with the recovery of regulatory T cells while a concomitant decrease of CD25+/ CD4+ T cells and CD38+/CD8+ T cells was observed (Ensoli et al, PLoS ONE 2010). Based on this data and considering that all doses were equally well tolerated and safe, three intradermal vaccinations with 30 µg of Tat was chosen as the optimal dosage and regime for the present clinical trial. This choice also offers additional benefits since it is less expensive, is administered without adjuvant and may favour better compliance to the treatment.

3 STUDY OBJECTIVES

Primary Objective
To investigate the immunogenicity of three 30 µg doses of a therapeutic recombinant, biologically active, HIV-1 Tat protein vaccine administered intradermally at 4 week intervals, as compared to placebo.

Secondary Objective
To investigate the safety of three 30 µg doses of a therapeutic recombinant, biologically active, HIV-1 Tat protein vaccine administered intradermally at 4 week intervals, as compared to placebo.

4 STUDY DESIGN

The study is a phase II, randomized, double-blinded, placebo controlled, clinical trial to evaluate the immunogenicity and safety of a therapeutic, biologically active HIV-1 Tat protein vaccine. HIV-1 positive volunteers will be recruited and screened. Volunteers will be eligible to participate based on the inclusion and exclusion criteria described in Section 6.1 and 6.2.
After a screening period of up to 35 days, the study duration will be 48 weeks, including an 8 week treatment phase (during which 3 vaccinations will be administered at 4-week intervals) and a 40 week follow-up phase.
This study will be conducted at 1 clinical site in South Africa. 200 Subjects will be randomized in a 1:1 ratio to one of the two treatment groups in accordance with the randomisation schedule detailed in section 5.6

5 INVESTIGATIONAL PRODUCT AND DOSAGE REGIMENS

5.1 Investigational product: Background on Investigational product preparation

The investigational product is a recombinant, biologically active Tat protein vaccine which will be provided by ISS. The Tat protein vaccine is produced by Diatheva-Avitech Srl and bottled and labelled by Injectalia Srl in accordance with GMP regulations. ISS will also provide the sterile water for the dilution of the Tat vaccine and placebo.

Three doses of either Tat vaccine 30 µg or placebo will be administered intradermally. The product constituents are as follows:

HIV-1 Tat vaccine 30 µg
Active substance: Biologically active Tat protein.
Buffer composition: Phosphate saline buffer, pH 7.4, 1% sucrose, 1% Human Serum Albumin.

Placebo:
Phosphate saline buffer, pH 7.4, 1% sucrose, 1% Human Serum Albumin.

Numbered kits of the investigational product (active or placebo) will be provided. ISS will retain a reference sample of each of the two products (Tat Vaccine 30 µg and Placebo) in their sample archive, in accordance with ICH-GCP guidelines.

5.1.1 Investigational Product Preparation

Each treatment kit contains 3 vials representing the complete vaccination schedule for one subject. Treatment kits must be stored at -80 °C and protected from light until the time of each administration.
The manufacturer has certified the extractable volume of each vial as 0.5 ml.

Immediately prior to the administration of the vaccine (≤ 40 minutes before administration), the vial contents should be allowed to thaw and then diluted with sterile water according to the following process:

1. Place the vial containing the vaccine (0.5 ml) on ice and protected from light.
2. Add 1.5 ml of sterile water for injection to the vial containing the vaccine. Keep the vial protected from light.
3. Mix by gently agitating the vial to obtain a homogeneous solution (2.0 ml total volume).
4. Prepare the syringe for the first injection: draw up 1.0 ml of the vaccine preparation.
5. Prepare the syringe for the second injection: draw up 1.0 ml of the vaccine preparation.
6. Administer the vaccine by four separate intradermal injections into the deltoids of the right and left upper arms (2 x 0.5 ml into two separate sites on the right arm and 2 x 0.5 ml into two separate sites on the left arm).

At the time of administration, the investigator (or designee) will complete the vial's label with the relevant information, and then attach the tear-off portion of the label to the appropriate investigational product administration form.

5.1.2Vaccination schedule

Tat 30 µg vaccine or placebo will be administered intradermally according to the following schedule:

- Group A: 3 intradermal administrations of Tat 30 µg at weeks 0, 4 & 8
- Group B: 3 intradermal administrations of placebo at weeks 0, 4 & 8

			Vaccination schedule		
**Group**	**Dose Level**	**No. of Participants**	**Week 0**	**Week 4**	**Week 8**
**A**	Tat 30 µg (id)	100	X	X	X
**B**	Placebo (id)	100	X	X	X
**Total**		200			

The administered dose of the investigational product will remain constant throughout the vaccination schedule.

5.2 Study Duration

Subjects will be screened for up to 35 days prior to treatment initiation, and will be followed for 48 weeks post first vaccination. Study start is estimated to be October 2011.

5.3 Investigational Product Import and Delivery

Investigational product will be supplied by ISS. Shipment of the IP from Italy to South Africa will be carried out by a specialized courier service, maintaining and monitoring the manufacturer specified storage requirements. IP will be transferred to a depot located in Johannesburg from where it will be dispatched to the trial site. On receipt of the investigational product at the site, it will be responsibility of the clinical site's pharmacist or designee to immediately verify that the shipment is intact, that the temperature conditions have been maintained during the shipment and to ensure that the study drug is immediately transferred to a -80 °C freezer with minimum exposure to light.

Numbered kits, one per subject, will be provided to the site in shipment blocks composed of 4 treatment kits each. Each kit will contain a set of 3 vials (containing either active or placebo product) required for the completion of one subject's vaccination schedule.

Re-supply of kits will follow the rate of recruitment so that the clinical site will be re-supplied when the set of 4 kits has been completely allocated to eligible subjects.

In the event of any vials becoming damaged or unusable during the preparation process, the investigator should immediately inform the Triclinium monitor to arrange for the replacement of the vial(s).

5.4 Packaging and Labelling of Investigational product

Each investigational product (active/placebo) vial will be packaged in a single box; three boxes, each containing one vial, will then be packaged in one kit-box. Each kit-box will contain investigational product for a complete vaccination schedule for one randomized subject.
Blocks of 4 kit-boxes will be packaged together and will correspond to the minimum quantity of investigational product shipped from the Johannesburg depot to the clinical site.

The investigational product will be packaged and labelled in accordance with the guideline, Version 4.01 - Guide to Good Manufacturing Practice for Medicines in South Africa, March 2009. The following information will be provided on the product labels in English:

Primary labels (Vials)

- name, address and telephone number of the Sponsor or Investigator
- pharmaceutical dosage form, route of administration, quantity of dosage units, name/identifier and strength/potency
- batch number
- trial protocol number
- trial subject identification number/treatment number and the visit number

Secondary labels (Vial boxes & Kit-boxes)

- name, address and telephone number of the Sponsor
- pharmaceutical dosage form, route of administration, quantity of dosage units, name/identifier and strength/potency
- batch number
- trial protocol number
- trial subject identification number/treatment number and where relevant the visit

number
- name of the investigator
- directions for use
- ‘For Clinical Trial Use Only’
- Storage conditions
- Period of use (use-by date, expiry date or re-test date as applicable) in month/year format

5.5 Investigational Product Storage and Accountability

The HIV-1 Tat vaccine and placebo must be stored at -80 °C and protected from light in a secure facility to which access is restricted to authorized individuals only.

It is the CROs responsibility to confirm that:
- *The IP is correctly received in South Africa, and that all specified storage conditions were adhered to during transport of the product from ISS to South Africa.*
- *The investigational product kits are distributed to the clinical site as and when required, and that all specified storage conditions were adhered to during transport.*

It is the investigator/clinical institution’s responsibility to make all reasonable efforts to ensure that:
- IP delivered is correctly received (the received product should be checked against the shipment record, and the acknowledgement of receipt form for the IP should be completed appropriately and faxed back to the shipper immediately upon receipt).
- The investigational product is stored under the required conditions in a secure facility to which access is restricted to authorized individuals only.
- The investigational product is only administered to eligible subjects in accordance with the protocol requirements
- All unused investigational product are returned to the Sponsor at the end of the study conduct.
- All used or partially used vials are destroyed according to the site’s operating procedures.

Temperature logs of the storage facility will be maintained by the clinical site, and will be checked by the CRA at each monitoring visit.

The investigational product will be administered to subjects by authorized personnel only (Principal Investigator or designee). A drug inventory will be maintained and will include details of all received, returned and destroyed investigational product. Drug accountability forms will also be maintained to detail all dispensed and administered vaccines and the subjects to whom they were administered.

At the conclusion of the clinical conduct of the study, all unused investigational product/placebo supplies will be returned to the Sponsor.

5.6 Randomization Codes

This study will be double-blinded. Subjects will be randomized upon confirmation of eligibility to receive either active or placebo investigational product in a 1:1 ratio. A randomization schedule will be generated using the SAS® procedure PROC PLAN. This will be generated and filed in such a manner so as to ensure that all study personnel remain

blinded until such time as the database is locked and the study unblinded. Randomisation will be performed in block sizes of 4.

Subjects will be allocated a sequential randomization number. The number will consist of a 3 digit-sequential number pre-fixed by a 1-digit unique site identifier.

6 METHODS

6.1 Inclusion Criteria

1. Male and female volunteers aged 18-45 years (inclusive)
2. Anti-Tat antibody negative
3. HIV-1 infected individuals currently receiving treatment with ARVs
4. Chronically suppressed HIV-1 infection as indicated by a HIV-1 plasma viraemia < 400 copies/ml and a CD4+ T cell count ≥ 200 cells/µl at screening, and documented at least once during the 12 month period prior to screening, irrespective of the pre-ARV CD4 nadir.
5. Negative pregnancy test for females of childbearing potential (not sterilized and still menstruating or within 1 year of the last menses) to be performed during the screening phase and immediately before each vaccination, and use of an acceptable method of contraception (double barrier methods, combined oral contraceptives, injectable contraceptives or intra-uterine devices) for at least 3 weeks prior to the first vaccination and for the duration of the study
6. Has provided written informed consent.
7. Agrees to stay in contact with the research site for the duration of the study

6.2 Exclusion Criteria

1. Acute illness on Study Day 0
2. Body temperature ≥37.5 °C on Study Day 0
3. Any current AIDS-related opportunistic disease

22. Any current neoplastic disease *[NOTE: Criterion is meant to exclude volunteers with active invasive cancer. Volunteers who present with intraepithelial neoplasia during the course of the study are to continue with all study procedures.]*
4. Known history of malignant neoplastic diseases [*NOTE: Subjects with known history of non-malignant neoplastic diseases that are completely resolved according to the fulfilment of all the specific recovery criteria, in agreement with the current guidelines in medical oncology, are eligible*]
5. Known history of encephalopathy, neuropathy or unstable CNS pathology, immunodeficiency, autoimmune disease, angina or cardiac arrhythmias, or any other clinically significant medical problems in the opinion of the investigator
6. Any evidence, as judged by the investigator, of unstable cardio-vascular disease (e.g. unstable hypertensive disease needing modification or introduction of an anti-hypertensive treatment)

7. Chest radiography showing evidence of active or acute cardiac or pulmonary disease within 6 months prior to the study screening visit [Note if no previous chest X-ray available, this will be performed at screening];
8. Known history of anaphylaxis or serious adverse reactions to vaccines
9. Known history of serious allergic reaction to any substance, requiring hospitalization or emergent medical care (e.g. Steven-Johnson syndrome, bronchospasm, or hypotension)
10. Active pulmonary tuberculosis within 12 months of screening as evidenced by chest radiography and/or medical history.
11. Any known medical or psychiatric condition which precludes subject compliance with the protocol, specifically, persons with psychotic disorders, major affective disorders and/or suicidal ideation are to be excluded
12. Current use of psychotropic drugs prescribed for major psychotic disorders
13. Concomitant participation in any study with an investigational product or device
14. Current or prior therapy with immunomodulator, immunosuppressive and/or anticoagulant drugs within 30 days prior to administration of the investigational product
15. Live attenuated vaccines within 60 days of the first administration of the investigational product [*NOTE: Medically indicated sub-unit or killed vaccines (e.g., influenza, pneumococcal, hepatitis A and B) are permitted, but must have been administered at least 4 weeks prior to the first administration of the investigational product*]
16. Known receipt of blood products or immunoglobulins during the year prior to screening
17. Previous participation in an HIV-1 vaccine trial (subjects who are known to have previously participated in the placebo arm of an HIV-1 vaccine trial, and so have never received an investigational HIV-1 vaccine, are eligible for inclusion)
18. Known drug and/or alcohol abuse in the year prior to screening
19. Use in the last 6 months or concomitant use of anti-CCR5 inhibitors and/or integrase inhibitors and/or fusion inhibitors
20. Pregnant or lactating females

6.3 Screening Failures

The investigator will maintain a log of all screening failures. A screening failure is defined as any subject who signs the informed consent form but who does not meet the inclusion/exclusion criteria and is thus not randomized to receive the investigational product.

6.4 Randomization

Volunteers will be allocated a screening number at the time of screening. This number will comprise the clinical trial code (T3) + a 2-digit site identifier (e.g. ‘01’) + a 3-digit sequential recruitment number. For example, the first subject screened at clinical site number 01 would be assigned the screening code T301001, the second subject screened would be T301002 and so on. All screened volunteers will be assigned a screening number, irrespective of whether or not they will be included in the study. Once allocated, screening

numbers will not be re-used. The screening number will be used as the main subject identifier throughout the whole study.

At the time of enrolment, subjects will be randomly assigned to a treatment regime in accordance with the randomization schedule. The site will allocate the next available sequential treatment number to each subject as they are randomized. Treatments will be allocated in balanced block-sizes of four. Allocation of the treatment number to a particular subject will only occur after confirmation of eligibility immediately prior to the first vaccination.

The original randomization list will be kept by Triclinium. A copy will be supplied to the GMP manufacturer in charge of the investigational product packaging and release.

6.5 Blinding/Un-blinding

Investigational product kits will be provided to the Investigators and their staff in a blinded fashion by the Sponsor. All clinical and laboratory staff and the subject will be blinded to treatment assignment (active treatment or placebo). Double-blinding will reduce the potential for observer bias and subject reporting bias.

Un-blinding will only occur at the end of the study after database lock or in the event of an emergency where knowledge of the treatment allocation is required for proper clinical management of the subject. If possible, sponsor approval should be sought prior to any emergency un-blinding procedure. In the interests of time, however, this may not be possible. The investigational site will be provided with randomization code envelopes together with the treatment kits. In the event of an emergency un-blinding, the investigator will record the reason for, and the date and time of the un-blinding in the source documents, the envelope and in the CRF.

The sponsor and CRO will be informed immediately of any emergency un-blinding that occurs at the site. This information will be communicated to the DSMB and will be detailed in the Clinical Study Report.

6.6 Study Assessments

The following section provides a detailed listing of the clinical, immunological and virological assessments to be performed during this study.

6.6.1 Clinical Assessments

All clinical assessments will be performed at the clinical site; special investigations e.g. chest X-ray will be performed at the nearest referral centre.

6.6.2Standard Laboratory Assessments

The following laboratory assessments will be performed at the time points described beneath in section 6.7

Haematology:
Red cell count, haemoglobin, haematocrit, MCV, platelets, white cell count and differential counts (neutrophils, lymphocytes, monocytes, eosinophils, basophils)

Clinical chemistry:
Sodium, Potassium, Calcium, Total Protein, Albumin, Total Bilirubin, Alkaline Phosphatase, AST, ALT, GGT, Urea, Creatinine, LDH, CPK, Thyroid function tests (T4 and TSH)

Coagulation:
Thrombin Time, Prothrombin Time, APTT

General Immunological parameters:
CD4+ T cell counts, HIV-1/2 ELISA determinations

Urinalysis:
Dipstick determination of Protein, Glucose, Nitrites, Albumin, Leukocytes and Red blood cells

Pregnancy test for females:
Serum βHCG will be conducted at screening for all females; urine pregnancy test to be performed on all females of child-bearing potential immediately prior to each vaccination and at designated follow-up visits.

The total quantity of blood to be collected for the purposes of this study will be kept to the minimum volume (about 460 ml) that will facilitate the assessment of immunology and virology parameters as well as all the primary safety determinations necessary to fulfil the requirement for first line immunology and virology testing. Second line immunology and virology testing will be performed depending on the availability of residual specimens (PBMC, serum, plasma).
First line testing will include the minimal panel of assays required to identify the presence of an immune response to Tat, which represents the primary endpoint of the study. Second line testing is aimed at allowing a more detailed assessment of the immune response to Tat and for an in depth exploration of immunologic and virologic biomarkers of ARV efficacy and/or disease progression.

6.6.3 Immunological Evaluations

Immunological evaluations will be performed according to "first line" and a "second line" laboratory testing by the designated Core Laboratory.

First line immunology testing
Assessment of anti-Tat humoral immune response:
- Determination of IgM, IgG and IgA anti-Tat antibodies in sera
- Titration of IgM, IgG and IgA anti-Tat antibodies

Second line immunology testing
The second line immunology testing will be performed retrospectively after the un-blinding, at the end of the study, on subgroups of participants defined on the basis of immunogenicity results.
In particular, lymphoproliferative responses (CFSE staining) or *in vitro* γIFN, IL-4 and IL-2 production in response to Tat (ELIspot) will be performed depending on the sample cell viability and recovery.

In addition, depending on the availability of residual specimens (PBMC, serum and plasma), the following tests will also be performed:

- Characterization of lymphocyte subsets (CD3, CD4, CD8, CD16, CD56, CD19)
- Anti-Tat IgG (IgG1, IgG2, IgG3, IgG4) subclasses
- Epitope mapping of IgM and IgG anti-Tat antibodies
- Neutralization of Tat activity by in vitro assays (rescue inhibition assay)
- Anti-HIV regulatory and structural proteins antibodies
- Antibody-mediated cellular cytotoxicity (ADCC)
- Inhibition of Tat uptake by MDDCs
- Neutralization of primary isolates (all clades)
- Anti-CCR5 antibodies
- Anti-CD4 antibodies
- Lymphoproliferative response to mitogens and recall antigens
- Lymphoproliferative response to HIV-1 Env (CFSE staining)
- *In vitro* γIFN, IL-4 and IL-2 production in response to Env (ICS/Elispot)
- B cells phenotype (naïve and memory) and antigen specific antibodies
- Phenotype and functional characterization of regulatory T cells
- Intracellular PBMC staining for granzyme, perforin, cytokines and chemokines
- Analysis of Th1 and Th2 cytokines in sera and in PBMC supernatants
- Analysis of chemokines in sera and in PBMC supernatants
- Lymphocytes spontaneous cell death and PBMC cell viability
- B cell cloning
- Functional and molecular characterization of clono-specific antibodies
- Serum/plasma determination of soluble CD4
- Serum/plasma determination of phenotypic and biochemical markers of immune activation (CD38 expression, neopterin, β2-microglobulin, C reactive protein, total immunoglobulin, sCD27, sCD14, LPS and IgM against endotoxin core antigen by ELISA)
- HLA typing.

6.6.4 Virology Evaluations

Virology evaluations will be performed according to “first line” and a “second line” laboratory testing by the designated Laboratory to determine:

First line virology testing - HIV-1 plasma viraemia (viral RNA copies)

Second line virology testing - The following parameters will be determined, depending on the availability of residual specimens (PBMC, serum, plasma):

- HIV-1 sequencing and phylogenetic analysis
- HIV-1 Genotypic resistance
- HIV Viral tropism
- HIV-1 proviral DNA copies
- Assessment of co-infections (e.g. Syphilis, HBV, HCV, HHV8 and others)

Female participants only: Cervical swabs and Pap smears will be taken on 3 occasions during the course of the study in order to test for Human Papilloma Virus (HPV) infection.

Should any of the above co-infections be detected during the course of the study, participants will be referred to their healthcare provider for clinical management.

In addition, the assessment of antiretroviral drug concentration may be performed.

6.6.5 Sample Collection, Storage and Shipment

The volume of blood to be collected at each study visit will vary depending on the specific analyses to be performed at that visit. Approximate volumes for the assessments are 31 ml for immunology and virology evaluations and 19 ml for assessing safety parameters. The total volume that can be collected at any one visit is ± 50 ml as per the following table:

<table>
<tr><th colspan="2">Assessment</th><th>Blood sample volume (ml)</th></tr>
<tr><td colspan="2">Immunology/Virology</td><td>31</td></tr>
<tr><td rowspan="2">Safety</td><td>Clinical chemistry</td><td>5</td></tr>
<tr><td>Haematology</td><td>14</td></tr>
<tr><td colspan="2">Total</td><td>50</td></tr>
</table>

Inclusive of screening evaluations, samples will be collected at 12 different time points throughout the study, resulting in the collection of approximately 460 ml blood over a period of at least 48 weeks. All blood samples will be collected by venipuncture.

General laboratory tests as detailed in section 6.6.2 will be performed at the NHLS Laboratory of the DGM Hospital. Blood samples for these tests will be collected at the Clinical Research Unit and transferred to the laboratory so that the testing procedures begin within three hours of sample collection. Results will be forwarded to the investigator as soon as available, in accordance with test-specific turn-around-times.

Specialised immunological and virological evaluations (as detailed in sections 6.6.3 and 6.6.4) will be performed in part at the NHLS Laboratory and at the designated Core Lab. A Core Laboratory of Immunology and Virology (*Core Laboratory of Immunology and Virology Ospedale S. Gallicano IFO, Rome)* will perform all the immunologic testing to assess the anti-Tat immune response and the virological assessments that go beyond the routine clinical monitoring of the patients.

Blood samples to be transferred to the NHLS and/or to the Core Laboratory for immunologic and virologic investigations will be collected and processed according to the procedures described by the protocol-specific Specimen Management Manual which will be provided to the investigational site prior to study initiation. Thereafter, cryopreserved cellular specimens and sera will be stored in liquid nitrogen or at -80 °C prior to be shipped to the Core Lab of immunology and virology (IFO San Gallicano Hospital, Rome). Shipment to the Core Lab will be done on dry ice, through a specialized courier, within 15 days of the date of sample collection.
Each sample will be identified with the following information: protocol number, centre number, subject screening number (i.e. subject identifier), visit number, sample type (plasma, serum or whole blood) and the sample collection date.

The residual specimens (PBMC, serum or plasma) available after the conduct of the first line tests, will either be used immediately, or kept frozen for later use, for the determination of additional second line tests (refer to sections 6.6.3 and 6.6.4).

As an exploratory test to detect possible HPV infection, cervical swabs and Pap smears will be collected from female participants at 3 time points throughout the study. Samples will be analysed at the S. Orsola Malpighi Hospital (Bologna, Italy).

6.7 Schedule of Study Procedures

The schedule of study visits and assessments is as follows:

Day -35 to -1: Screening Period

Informed consent for the study will be obtained prior to any study specific procedures.
At screening, the following procedures will be performed:

- Signed informed consent
- Medical History (including HIV-specific and TB history);
- Previous and concomitant medication history (including a complete history of all anti-retroviral treatments taken by the subject as far as possible)
- Complete physical examination including height and weight;
- Vital signs after 5 minutes in a resting position (seated blood pressure, heart rate, respiratory rate and axillary body temperature);
- Counselling: Pre- & post-HIV test counselling, ARV adherence, risk reduction and the prevention of pregnancy
- Serum βHCG pregnancy test (for all females)
- Standard haematology, clinical chemistry, coagulation and urinalysis assessments (as described in section 6.6.2)
- Chest x-ray if an x-ray examination performed within 6 months prior to screening is not available to exclude active or acute cardiac or pulmonary disease, including Pulmonary Tuberculosis.
- Thyroid function tests (T4 & TSH)
- $CD4^+$ T cell
- Virological evaluation: HIV-1/HIV-2 ELISA
- HIV-1 plasma viraemia
- Anti-Tat antibodies (IgM, IgG, IgA)
- Assessment of eligibility as per inclusion and exclusion criteria, and results of all assessments performed.

DAY 0 (Baseline)

Prior to the vaccine administration:

- Physical examination including weight;
- Vital signs after 5 minutes in a resting position (seated blood pressure, heart rate, respiratory rate and axillary body temperature)
- Concomitant medication
- Standard haematology, clinical chemistry, coagulation and urinalysis assessments (as described in section 6.6.2)
- Urine dipstick pregnancy test (for females of child-bearing potential only)
- CD4+ T cell counts
- HIV-1 plasma viraemia
- Anti-Tat antibodies (IgM, IgG,IgA)
- Cervical swab and Pap smear for detection of HPV infection (Female participants only)
- Review of medical history and eligibility criteria (before the first vaccination)
- Immunological & Virological sample collection and storage.

Note: If the subject has a body temperature ≥ 37.5 °C or, in the investigator’s opinion, an acute illness, **DO NOT VACCINATE**; re-schedule the Day 0 visit.

Vaccine administration:
The next available treatment kit (sequential number) at the site will be allocated to the subject as described in section 6.4. The vaccine will be diluted and administered as described in section 5.1.

Post-vaccination procedures:
After administration of the investigational product, subjects will remain at the study centre for 2 hours for the following assessments, at 0.5 hours, 1 hour and 2 hours after vaccination respectively:

- Symptom-directed physical examination
- Vital signs after 5 minutes in a resting position (seated blood pressure, heart rate, respiratory rate and axillary body temperature)
- Assessment of adverse events.
- Distribute and review diary card and diary card instructions.

During this period the subject will not consume any food or beverages other than water.
Prior to discharge from the clinical facility, subjects will be counselled on ARV adherence, risk reduction and the prevention of pregnancy.

After Day 0 (the first vaccination) the next 2 vaccination visits will be scheduled at 28-day (4 week) intervals. The next visit will be scheduled for 7 days (± 1 day) later.

DAY 7: 1st Vaccination Safety Follow-up (D7 ± 1 day)

- Collect diary card and review entries for completeness and accuracy
- Concomitant medication and adverse event review
- Standard haematology, clinical chemistry, coagulation and urinalysis assessments (as described in section 6.6.2)
- Vital signs after 5 minutes in a resting position (seated blood pressure, heart rate, respiratory rate and axillary body temperature).

DAY 28: 2nd Vaccination (D28 + 7 days)
Prior to the vaccine administration:

- Physical examination including weight;
- Vital signs after 5 minutes in a resting position (seated blood pressure, heart rate, respiratory rate and axillary body temperature)
- Concomitant medication and adverse event review
- Urine dipstick pregnancy test (for females of child-bearing potential only)
- $CD4^+$ T cell counts
- HIV-1 plasma viraemia
- Anti-Tat antibodies (IgM, IgG,IgA)
- Immunological & Virological sample collection and storage.

Note: If the subject has a body temperature ≥ 37.5 °C or in the investigator's opinion, an acute illness, DO NOT VACCINATE; re-schedule the vaccination visit within the protocol specified window period i.e. Day 28 + 7 days.

Vaccine administration:
The next unused vial will be removed from the treatment kit previously allocated to the subject. The vaccine will be diluted and administered as described in section 5.1.

Post-vaccination procedures:
After administration of the investigational product, subjects will remain at the study centre for 2 hours for the following assessments, provided at 0.5 hours, 1 hour and 2 hours after vaccination, respectively:

- Symptom-directed physical examination.
- Vital signs after 5 minutes in a resting position (seated blood pressure, heart rate, respiratory rate and axillary body temperature).
- Assessment of adverse events.
- Distribute and review diary card and diary card instructions.

During this period the subject will not consume any food or beverages other than water.

Prior to discharge from the clinical facility, subjects will be counselled on ARV adherence, risk reduction and the prevention of pregnancy. The next visit will be scheduled for 7 days (Day 35 ± 1 day) later.

DAY 35: 2nd Vaccination Safety Follow-up (D35 ± 1 day)

- Collect diary card and review entries for completeness and accuracy
- Concomitant medication and adverse event review.
- Standard haematology, clinical chemistry, coagulation and urinalysis assessments (as described in section 6.6.2).
- Vital signs after 5 minutes in a resting position (seated blood pressure, heart rate, respiratory rate and axillary body temperature).

DAY 56: 3rd Vaccination (Day 56 +7 days)
Prior to the vaccine administration:

- Physical examination including weight
- Vital signs after 5 minutes in a resting position (seated blood pressure, heart rate, respiratory rate and axillary body temperature)
- Concomitant medication and adverse event review
- Urine dipstick pregnancy test (for females of child-bearing potential only)
- $CD4^+$ T cell counts
- HIV-1 plasma viraemia
- Anti-Tat antibodies (IgM, IgG,IgA)
- Immunological & Virological sample collection and storage.

Note: If the subject has a body temperature ≥ 37.5 °C or in the investigator’s opinion, an acute illness, DO NOT VACCINATE; re-schedule the vaccination visit within the specified window period i.e. Day 56 + 7 days.

Vaccine administration:
The next unused vial will be removed from the treatment kit previously allocated to the subject. The vaccine will be diluted and administered as described in section 5.1.

Post-vaccination procedures:
After administration of the investigational product, subjects will remain at the study centre for 2 hours for the following assessments provided at 0.5 hours, 1 hour and 2 hours after vaccination respectively:

- Symptom-directed physical examination.

- Vital signs after 5 minutes in a resting position (seated blood pressure, heart rate, respiratory rate and axillary body temperature).
- Assessment of adverse events.
- Distribute and review diary card and diary card instructions.

During this period the subject will not consume any food or beverages other than water.

Prior to discharge from the clinical facility, subjects will be counselled on ARV adherence, risk reduction and the prevention of pregnancy. The next visit will be scheduled for 7 days (Day 63 ± 1 day) later.

DAY 63: 3rd Vaccination Safety Follow-up (D63 ± 1 day)

- Collect diary card and review entries for completeness and accuracy
- Concomitant medication and adverse event review
- Standard haematology, clinical chemistry, coagulation and urinalysis assessments (as described in section 6.6.2)
- Vital signs after 5 minutes in a resting position (seated blood pressure, heart rate, respiratory rate and axillary body temperature).

DAY 84: First follow-up visit (Day 84 ± 7 days)

The following procedures and assessments will be performed on all subjects:

- Physical examination including weight
- Vital signs after 5 minutes in a resting position (seated blood pressure, heart rate, respiratory rate and axillary body temperature)
- Concomitant medication and adverse event review
- Standard haematology, clinical chemistry, coagulation and urinalysis assessments (as described in section 6.6.2)
- $CD4^+$ T cell counts
- HIV-1 plasma viraemia
- Anti-Tat antibodies (IgM, IgG, IgA)
- Immunological & Virological sample collection and storage
- Counselling on ARV adherence, risk reduction and the prevention of pregnancy.

DAY 112: Second follow-up visit (Day 112 ± 7 days)

The following procedures and assessments will be performed on all subjects:

- Physical examination including weight
- Vital signs after 5 minutes in a resting position (seated blood pressure, heart rate, respiratory rate and axillary body temperature)
- Concomitant medication and adverse event review
- Standard haematology, clinical chemistry, coagulation and urinalysis assessments (as described in section 6.6.2)
- $CD4^+$ T cell counts
- HIV-1 plasma viraemia
- Anti-Tat antibodies (IgM, IgG, IgA)
- Immunological & Virological sample collection and storage
- Counselling on ARV adherence, risk reduction and the prevention of pregnancy.

DAY 140: Third follow-up visit (Day 140 ± 7 days)

The following procedures and assessments will be performed on all subjects:

- Physical examination including weight

- Vital signs after 5 minutes in a resting position (seated blood pressure, heart rate, respiratory rate and axillary body temperature)
- Concomitant medication and adverse event review
- Standard haematology, clinical chemistry, coagulation and urinalysis assessments (as described in section 6.6.2)
- $CD4^+$ T cell counts
- HIV-1 plasma viraemia
- Anti-Tat antibodies (IgM, IgG,IgA)
- Cervical swab and Pap smear for detection of HPV infection (Female participants only)
- Immunological & Virological sample collection and storage
- Counselling on ARV adherence, risk reduction and the prevention of pregnancy.

DAY 168: Fourth follow-up visit (Day 168 ± 7 days)

The following procedures and assessments will be performed on all subjects:

- Physical examination including weight
- Vital signs after 5 minutes in a resting position (seated blood pressure, heart rate, respiratory rate and axillary body temperature)
- Concomitant medication and adverse event review
- Standard haematology, clinical chemistry, coagulation and urinalysis assessments (as described in section 6.6.2)
- $CD4^+$ T cell counts
- HIV-1 plasma viraemia
- Anti-Tat antibodies (IgM, IgG,IgA)
- Immunological & Virological sample collection and storage
- Counselling on ARV adherence, risk reduction and the prevention of pregnancy.

DAY 336: Study termination (Day 336 ± 7 days)

The following procedures and assessments will be performed on all subjects:

- Physical examination including weight
- Vital signs after 5 minutes in a resting position (seated blood pressure, heart rate, respiratory rate and axillary body temperature)
- Concomitant medication and adverse event review
- Standard haematology, clinical chemistry, coagulation and urinalysis assessments (as described in section 6.6.2)
- Urine dipstick pregnancy test (for females of child-bearing potential only)
- $CD4^+$ T cell counts
- HIV-1 plasma viraemia
- Anti-Tat antibodies (IgM, IgG, IgA)
- Cervical swab and Pap smear for detection of HPV infection (Female participants only)
- Immunological & Virological sample collection and storage
- Counselling on ARV adherence, risk reduction and the prevention of pregnancy.

DAY 343: Dissemination of results (Day 343 ± 7 days)

Within 7 days of the study termination visit, a site staff member will telephonically contact the subject to inform them of the results of the following tests done at the study termination visit:

- Standard haematology, clinical chemistry, coagulation and urinalysis assessments
- Urine dipstick pregnancy test (for females of child-bearing potential only)

- $CD4^{+}$ T cell counts
- HIV-1 plasma viraemia

Results of the Pap smear will be telephonically communicated to the subject as soon as they become available.

In both cases, if results are abnormal and require that the subject be referred to a health facility, the subject will be requested to visit the site for the collection of a referral letter.

6.8 Compliance

IP will be administered by designated clinical study personnel at the clinical site only.
Treatment compliance will be verified by the CRA during monitoring visits. Drug accountability records will be completed according to the SOPs of the CRO and will be crossed-checked with the investigational product at the site during monitoring visits.

The subject will be reminded of the importance of attending the visits according to the planned timelines as strictly as possible.

7 PREVIOUS AND CONCOMITANT MEDICATION

A reasonable effort will be made to determine all relevant pharmacological and non-pharmacological treatments received by the subject during the 4 month period prior to the screening visit.

Subjects are permitted to receive standard therapy for any medical condition, including treatment for any viral or bacterial or other infections that develop during the study.

Chronic stable therapy (e.g., antihypertensive therapy) will be permitted throughout the study provided that subjects remain on a constant dose from 30 days prior to the first vaccination administration until the end of the study duration.

All previous and concomitant treatments will be recorded in the CRF, including the name of the treatment (if pharmacological, only generic names are required unless it is a combination product where the trade name should be documented), indication, route, total daily dose and start/stop dates.

7.1 Antiretroviral Therapy

Subject should receive standard antiretroviral therapy in accordance with their clinical condition and local, current guidelines.
As far as possible, a complete antiretroviral treatment history, starting from the first antiretroviral therapy taken by the subject, must be recorded in the CRF.

7.2 Prohibited Medication/Therapy

The following medications will not be allowed for the duration of the study period:

- *antipsychotic medications (throughout the study period);*
- *immunomodulators (for 30 days prior to screening and throughout the study period);*
- *immunosuppressants (for 30 days prior to screening and throughout the study period);*
- *anticoagulants (for 30 days prior to screening and throughout the study period);*

- *blood products or immunoglobulins (for 1 year prior to screening and throughout the study period);*
- *live attenuated vaccines (for 60 days prior to the first administration of the investigational product and throughout the study period); and/or*
- *CCR5 inhibitors and/or integrase inhibitors and/or fusion inhibitors (for 6 months prior to screening and throughout the study period)*

A subject who takes any of the above-mentioned treatments during the 8 week active treatment period will be withdrawn from further vaccination, but will continue to be followed for safety for the entire study period. Their data will however not be used in the immunogenicity analysis.

8 ENDPOINTS

8.1 Primary Endpoint Variable and Measurements

In order to evaluate the immunogenicity of Tat protein in the study population, the following primary endpoint will be considered:

- Induction of specific anti-Tat humoral immune response in terms of IgM, IgG or IgA anti-Tat antibodies.

The induction, magnitude and persistence of the humoral response to the administered vaccination schedule will be compared between the active and placebo groups.

The induction of the anti-Tat specific **humoral immune response** will be evaluated in the active as compared to the placebo group as follows:

1. Percentage of "responders", defined as those subjects that develop an immune response greater than the following levels:
   - *Anti-Tat IgM titers ≥ 25*
   - *Anti-Tat IgG titers ≥ 100*
   - *Anti-Tat IgA titers ≥ 25*
2. Geometric mean antibody titers (GMT) induced by vaccination.

8.2 Secondary Endpoint Variables and Measurements

In order to evaluate the safety of the Tat protein vaccination schedule in the study population, the following secondary endpoints will be considered and compared between active and placebo groups:

- Changes in physical examination findings from baseline;
- Changes in vital signs from baseline;
- Adverse events (including local and systemic reactions to the vaccination schedule occurring during the course of the study);
- Changes in standard laboratory safety parameters from baseline

9 SAFETY

9.1 Responsibility to Ensure the Safety of Trial Subjects

The national regulatory authority, the sponsor (ISS), the institutions through which the research is performed and all members of the principal investigator's clinical team share responsibility for insuring that subjects in this trial are exposed to the least possible risk of adverse events that may result from participation in this protocol.

9.1.1 Principal Investigator

The Principal Investigator (PI) has a personal responsibility to closely monitor trial subjects and to ensure their safety. The principal investigator has the authority to request that the Sponsor convene the DSMB in the event of any safety concerns, and may delay an individual's study vaccine administration if the investigator has some suspicion that the study vaccine might place a subject at significant risk. The principal investigator or qualified designee determines severity and causality with respect to the investigational vaccine for each adverse event. As the study will be conducted on patients taking antiretroviral therapy, the management of subject safety should take into consideration the known adverse reactions caused by the drugs used in antiretroviral combination therapy, and should also explore the possibility of any "antiretroviral drug exchange" practiced by the subject. The principal investigator will be blinded for this study. In the event of an emergency where knowledge of the treatment allocation is required for proper clinical management of the subject, the principal investigator may require the un-blinding of the treatment assignment (active treatment or placebo). If possible, sponsor approval should be sought prior to any emergency un-blinding procedure (See Section 6.5).

9.1.1.1 Study Vaccine Pausing Rules for the Principal Investigator

In the event of a safety concern, the principal investigator has the authority to request that the Sponsor convene an ad hoc review of the safety data by the DSMB. Based on this review, the DSMB may choose to pause further vaccine administration in the study. (See Section 10.)

9.1.2 Study Sponsor

The Sponsor has an institutional responsibility to ensure subject safety. This responsibility is vested in a Data Safety Monitoring Board.

9.1.2.1 Local Medical Monitor (LMM)

The LMM is the sponsor's representative and is a registered medical practitioner in his/her country of residence with experience in HIV/AIDS related research and HAART treatment. The LMM will regularly review all safety data while being cognizant of the known adverse reactions caused by drugs used in antiretroviral combination therapy. The LMM may assist the Principal Investigator in the assessment of severity and causality for adverse events, and help the PI in exploring the possibility of any "antiretroviral drug exchange" that may have been practiced by the subject. In addition to the ongoing review of adverse events, the LMM, by virtue of his/her location will be immediately available to discuss the significance and possible impact of a SUSAR/SAE with the principal investigator. The LMM, like the principal investigator, will be blinded.

9.1.2.1.1 Study Vaccine Pausing Rules for the Local Medical Monitor

In the event of a safety concern the Local Medical Monitor in collaboration with the Principal Investigator will request that the Sponsor convene an ad hoc review of the relevant safety data

by the DSMB. Based on this review, the DSMB may choose to pause further vaccine administration in the study (See Section 10.)

9.1.3 Data Safety Monitoring Board (DSMB)

The Sponsor will provide for the constitution of the Data Safety Monitoring Board for the monitoring of subjects' safety. The DSMB is composed of medical specialists with experience in HIV/AIDS-related clinical research and evaluation of investigational product safety issues. The primary responsibility of the DSMB is to protect the safety of subjects enrolled in the trial, by regular review of all safety data to verify no adverse changes to the pre-study risk-benefit ratio. The members cannot be directly involved with the conduct of the study. Additional subject area experts may be present to provide expertise if requested by the DSMB. According to their requirements the DSMB may review an individual SAE, or it may choose to review adverse events, serious adverse events, solicited adverse events, and laboratory and vital signs data. The DSMB may un-blind any amount of safety information needed to conduct their assessment. All procedures associated with this review, including objectives, data handling, and elements to be included for review will be documented in the DSMB minutes.

Based on its review of the safety data, the DSMB will make recommendations in the DSMB minutes to ISS regarding further conduct of the study and further administration of study treatment. The conclusions of the DSMB will be communicated to the Sponsor, Principal Investigator, Local Medical Monitor, Ethics Committees and the national regulatory authority for their concurrence. The Sponsor agrees to abide by the decision of its DSMB with concurrence of the national regulatory authority and the Ethics Committee.

9.2 Safety Surveillance during the Study

Subjects will be monitored and safety data collected by way of clinical interviews, examinations, and evaluations of daily diaries conducted by study team members and through reports of laboratory evaluations.

9.2.1 Adverse Event (AE)

An adverse event (AE) is defined as any unanticipated problem involving risks to study participants or others. An adverse event, therefore, can be any unfavorable or unintended sign, symptom, disease, syndrome, abnormal laboratory finding, or concurrent illness that emerges or worsens relative to the subject's pre-treatment baseline, whether or not it is considered to be related to the medicinal product.

All conditions that exist prior to administration of the study vaccine (pre-existing conditions) will be recorded in the subject's medical history to establish baseline. Day-to-day fluctuations in pre-existing conditions that do not represent a clinically significant change in the subject's status will not necessarily be reported as adverse events.

Any adverse change from the subject's baseline condition (determined from screening evaluations conducted to confirm study eligibility) that occurs following the administration of the study vaccine will be considered an adverse event. This includes the occurrence of a new adverse event or the worsening of a baseline condition, whether or not considered related to the study vaccine. Intermittent conditions such as headaches may be present on Study Day 0 but may represent an adverse event if the intensity or duration of the event is worse than usual following receipt of study vaccine. Adverse events include but are not limited to:

adverse changes from baseline that represent increases in toxicity grade according to the Toxicity Table, adverse changes in the general condition of the subject, signs and symptoms noted by the subject, concomitant disease with onset or increased severity after study vaccine administration, and clinically significant changes in laboratory safety parameters occurring after study vaccine administration.

The reporting period for all adverse events is the entire study period of 48 weeks. Adverse events will be reported on the Adverse Event CRF using a recognized medical term or diagnosis that accurately reflects the event. Adverse event evaluations will be reviewed by the principal investigator or by a designated medically qualified practitioner. Adverse event CRF pages are to be completed by members of the study team designated in writing by the principal investigator. The onset and resolution dates of the event and action taken in response to the event will be documented.
All adverse events must be followed until resolved or stabilized. The resolution date will be recorded on the CRF as the last date on which the subject experienced the adverse event or, if unresolved by the last day of the reporting period, the resolution date will be recorded on the CRF as "ongoing." Information recorded on the CRF must be substantiated in the source documents. If an adverse event evolves into a condition that becomes "serious," it will be designated as serious on the Adverse Event CRF and a SAE Report (SAER) form will be completed.

9.2.2 Serious Adverse Event (SAE)

Seriousness refers to the outcome of an adverse event. Seriousness is determined by the principal investigator (or qualified designee). If any of the following outcomes are present then the adverse event is serious:

- Results in death
- Is life-threatening (defined as an event in which the subject or patient was at risk of death at the time of the event; it does not refer to an event which hypothetically might have caused death if it were more severe)
- Requires inpatient hospitalization or prolongation of existing hospitalization
- Results in persistent or significant disability/incapacity
- Is a congenital anomaly/birth defect
- Is an important medical event [defined as a medical event that may not be immediately life-threatening or result in death or hospitalization but, based upon appropriate medical and scientific judgment, may jeopardize the patient/subject or may require intervention (e.g. medical, surgical) to prevent one of the other serious outcomes listed in the definition above]. Examples of such events include, but are not limited to, intensive treatment in an emergency room or at home for allergic bronchospasm, blood dyscrasias or convulsions that do not result in hospitalization.

A **serious adverse event** is an adverse event meeting the outcome criteria for seriousness regardless of relationship to an administered medicinal product.

9.2.3 Adverse Drug Reaction (ADR)

An ADR is any untoward and unintended response to any dose of an investigational medicinal product administered in a clinical trial when the Principal Investigator (or qualified

designee) has judged that there is at least a reasonable possibility that the event was related to the product.

9.2.4 Serious Adverse Drug Reaction (SADR)

A SADR is a SAE suspected to be causally related to the investigational product; a SUSAR is a Serious Unexpected Suspected Adverse Drug Reaction or unexpected SADR.

9.3 Reporting of Adverse Events/Adverse Drug Reactions

The identification and reporting of AEs/ADRs is the Investigator's responsibility. The collection of AE/ADR information will begin as soon as the study starts (when the first subject is enrolled).
AEs/ADRs may be either spontaneously reported or elicited during questioning and examination of a subject. All identified AEs/ADRs must be recorded and described on the appropriate page of the CRF in occasion of each visit scheduled within the clinical trial. If known, the diagnosis of the underlying illness or disorder should be recorded, rather than its individual symptoms.
Any clinically significant changes noted during interim or final physical examinations, electrocardiograms, haematological-biochemical-coagulation analysis, urinalysis and any other potential safety assessments, whether or not these procedures are required by the protocol, should also be recorded on the appropriate AE page of the CRF.

9.3.1 Assessing Severity

The safety concepts of "severity" and "seriousness" are distinct concepts. Severity refers to the degree of clinical manifestation. "Seriousness" refers to pre-defined outcomes from an adverse event. A severe adverse event is not always serious and a serious adverse event is not always severe.

For all adverse events the investigator (or qualified designee) is responsible for assessing the severity of the event and the causal relationship of the event to the study vaccine.

The severity of all adverse events, including clinical findings and abnormal laboratory values, will be classified as one of the following grades:

1. Mild
2. Moderate
3. Severe

A Toxicity Table (adapted for this protocol from the Division of Aids table for grading the severity of Adult and Pediatric Adverse Events Version 1.0, December, 2004; clarification August 2009) is provided as an appendix to the protocol for the assessment of severity of specified adverse events. The Toxicity Table Adverse Event Grades do not correlate directly with the classical severity grades of mild, moderate and severe. **For the purposes of recording events in the CRF** Toxicity Table Grade 1 events will be considered mild in severity, Toxicity Table Grade 2 & 3 events will be considered moderate in severity, and Toxicity Table Grade 4 events will be considered as severe.

For adverse events not listed in the Toxicity Table determination of severity requires some level of interpretation as outlined below. The degree of incapacity caused by the adverse event and the level of medical intervention required for treatment may be helpful in assessing the overall severity of the adverse event.

For example:

- Mild events are generally regarded as noticeable but have no impact on normal activities; they may or may not require over-the-counter treatment managed by the subject.
- Moderate events generally have some impact on an individual's normal activities and may require general symptomatic medical intervention by a healthcare professional or by the subject.
- Severe adverse events may be incapacitating, leading to suspension of normal daily activities, and would generally require more immediate medical evaluation and intervention by a healthcare professional.

A change in severity of an adverse event will not be recorded as a new adverse event. Only the highest severity level that occurs during the entire period of the adverse event will be recorded on the CRF with the onset and resolution dates encompassing the entire duration of the event.

9.3.2 Assessing a Causal Relationship (Relatedness)

For all adverse events, the investigator determines a **causal relationship** to the study vaccine without the knowledge of whether the Tat protein vaccine or placebo was administered. A number of factors will be considered in making this assessment, including:

1. the temporal relationship of the event to the administration of the study vaccine
2. whether an alternative etiology has been identified and
3. clinical plausibility.

The investigator will be requested to assess the causal relationship of an adverse event to study vaccine using the following criteria:

Descriptor	Definition
Certain	The study vaccine is known to be the cause of the adverse event. The evidence establishes a causal relationship; an association exists between the event and receipt of the study vaccine and there is a plausible mechanism for the event to be related to the study vaccine, and causes other than the study vaccine have been ruled out.
Probable	It is likely that the adverse event was caused by administration of the study vaccine. The evidence favours acceptance of a causal relationship; an association exists between the event and receipt of the study vaccine and there is a plausible mechanism for the event to be related to the study vaccine, and an alternative aetiology is not apparent.
Possible	There is a reasonable possibility that the adverse event was caused by study vaccine. There must be a plausible mechanism for the event to be related to study vaccine. The evidence is inadequate to accept or reject, or favours rejection of, a causal relationship; an association exists between the event and the study vaccine but there may also be an alternative aetiology, such as characteristics of the subject's clinical status or underlying condition.
Unlikely	There is less than a reasonable possibility that the adverse event

	was caused by study vaccine
Unrelated	There is no evidence of a causal relationship; another aetiology is known to have caused the adverse event. The alternative aetiology should be documented in the subject's study record.

The investigator may be assisted by the local medical monitor in determining causality. It is expected that in the event of any uncertainty, communication and consultation may occur in the assessment of the causality of adverse events. Certain, probable and possible are considered to be related. Unrelated and unlikely related are considered to be unrelated.

Every effort should be made by the investigator to determine the existence of any pre-existing conditions that should be taken into consideration when assessing the causal relationship of an adverse event. Pre-existing conditions should be recorded in the CRF as baseline medical history.

9.4 Adverse Event Treatment, Follow-up and Outcome

Treatment of any adverse events will be determined by the investigator using his/her best medical judgment and according to current clinical practice guidelines. All applied measures as well as follow-up will be recorded in the appropriate CRF. Adverse events will be considered resolved when the condition returns to normal or returns to the subject's baseline status as established on Study Day 0, or when the condition has stabilized with the expectation that it will remain chronic. Since the principal investigator will be blinded for this study, in the event of an emergency where knowledge of the treatment allocation is required for proper clinical management of the subject, the principal investigator may require the un-blinding of the treatment assignment (active treatment or placebo). If possible, sponsor approval should be sought prior to any emergency un-blinding procedure.

The investigator will continue follow-up on adverse events, including clinically significant laboratory abnormalities until the event has resolved, is otherwise satisfactorily explained, or the subject completes the study. If not reported earlier, the outcome of adverse events will be determined when the subject completes the study.

Follow-up for serious adverse events must continue until resolution and the outcome reported to ISS, even if this extends beyond the serious adverse event reporting period (i.e., after the final study visit). For analysis purposes, the outcome for serious adverse events will be determined on the final study visit.
If at any time after completion of the serious adverse event reporting period (the final study visit) the investigator becomes aware of a serious adverse event that is suspected by the investigator to be related to the study vaccine, the event must be reported to ISS.

9.5 Reporting of Serious Adverse Events/Serious Adverse Drug Reactions

Serious adverse events, which include SUSARs, are to be reported to the sponsor for the entire study period. SUSARs are reported even after the trial is over, if the sponsor, local medical monitor or principal investigator becomes aware of them. The site will be provided with specific reporting procedures including the Adverse Event CRF and any supplemental reporting forms to be used. Serious adverse events will be reported on the Adverse Event CRF using a recognized medical term or diagnosis that accurately reflects the event.

Serious adverse events will be assessed by the investigator as described in Section 9.1.1 for severity, causal relationship to the study vaccine, and expectedness. The onset and resolution dates of the event and the action taken in response to the event will be documented. If the event has not resolved by the final study visit, it will be documented as "ongoing" on the CRF, however, follow-up of the SAE must continue until resolved. Information recorded on the CRF must be substantiated in the source documents.

The AE CRF containing a serious adverse event and the SAE Report completed for that event must be **faxed** by the principal investigator or his/her designee **within 24 hours** (one calendar day) of the investigative site becoming aware of the event to the local medical monitor and to Triclinium. The AE CRF should be completed with all information known at the time and a hard copy printed for faxing; the SAE Report (paper form) should be completed and both forms faxed (even if all information concerning the event is not yet known) within the first 24 hours of awareness of the event.

All serious adverse events that the investigator suspects are related to the study vaccine (SUSARs) should be **telephoned to the local medical monitor and to Triclinium immediately upon the investigator's awareness of the event.** Since the principal investigator will be blinded for this study, in the event of an emergency where knowledge of the treatment allocation is required for proper clinical management of the subject, the PI may require the un-blinding of the treatment assignment (active treatment or placebo). If possible, sponsor approval should be sought prior to any emergency un-blinding procedure.

Contact information for all safety personnel are contained in the Team Contact List which will be stored on site in the investigator Site File and maintained by Triclinium.

Investigators **must not wait** to collect additional information to fully document the event before notifying the local medical monitor and Triclinium of a serious adverse event. The initial notification should include the following (at minimum):

- Protocol number and name and contact number of the investigator
- Subject study number (and initials and date of birth, if available)
- Date/s subject received study vaccine
- Serious adverse event(s) and date of event onset
- Current status of subject

ISS has authorized Triclinium to execute its responsibilities for safety report submission to the appropriate regulatory authorities within specific time periods of being notified of the event; the investigative site is responsible for notification of all SAE reports to the relevant Ethics Committee within the appropriate reporting time periods. Triclinium will notify the DSMB of all SAEs within three working days of becoming aware of an event and will provide all follow-up information in a timely manner.

9.6 Other Events Requiring Immediate Reporting

The investigator must report the following events by faxing the appropriate form to the local medical monitor and Triclinium within 24 hours of becoming aware of the event; Triclinium will forward the appropriate form to ISS:

- Withdrawal of consent during the study (Immediately Reportable Event Form)
- Emergency un-blinding (Immediately Reportable Event Form)

- Protocol violation affecting the safety of a subject or involving the vaccination process (Immediately Reportable Event Form)
- Adverse event thought to be an allergic reaction to the study vaccine (Immediately Reportable Event Form, unless event meets SAE criteria)
- Any event that, in the opinion of the investigator, precludes further administration of the study vaccine (Immediately Reportable Event Form, unless meets SAE criteria)
- Pregnancy (Immediately Reportable Event Form, and Pregnancy Notification Form)

In each case the investigator will make every effort to keep the subject in the study until the end of the follow-up period (week 48); if this is not possible, the final follow-up investigations will be conducted at the time of study termination. In case of study termination, the subject will be managed in accordance with the investigator's clinical judgment.

In case a subject misses a scheduled vaccination visit, the study staff will try to establish communication with him/her through all possible means (e.g., call the subject, contact his/her ARV clinic,) and will attempt to reschedule the vaccination visit within the 7 day window period; after this period the subject will be withdrawn from further vaccination, on the basis of a non-compliance to the protocol.
The requirement to attend all scheduled study visits will be emphasized to subjects at each visit.

9.7 Follow-up of Subjects Who Become Pregnant

Female subjects will be advised of the unknown risk of study vaccine to the unborn fetus and will be advised to use adequate birth control methods for at least 3 weeks prior to vaccination and for the whole the duration of the study.

If a subject becomes pregnant during the study, she should be encouraged to continue in the study for safety follow-up. Follow-up should continue for pregnancy outcome including premature terminations, and data are to be included in the safety reports.

The investigator must notify the Local Medical Monitor and Triclinium of the pregnancy immediately (even if already known to have resulted in spontaneous or elective abortion) by fax. At a minimum, the estimated date of conception, the estimated due date, and the date the subject received the study vaccine should be provided.

If a subject becomes pregnant, she will not have blood drawn as normally mandated by the protocol, but will undergo all other evaluations according to the Schedule of Procedures.

The health status of the mother and child, the date of delivery, and the child's sex and birth weight should be reported after delivery. If delivery occurs before the final study visit, the subject should continue to be followed for SAEs through the final study visit unless withdrawal of consent has occurred. If delivery occurs after the final study visit, the investigator should attempt to maintain contact with the subject to obtain information after delivery.

Pregnancy will not be recorded as an adverse event. If the pregnancy results in a miscarriage or a planned termination, the event (spontaneous abortion or elective abortion) will be reported as an adverse event or a serious adverse event per the investigator's judgment (e.g.,

if it was a medically important or life-threatening event that meets the definition of a serious adverse event).
A congenital anomaly or birth defect (i.e., an adverse finding in a child or fetus of a subject) within 12 months of the last exposure to the IP, must be reported as a serious adverse event.

If it is determined after completion of the study that a subject became pregnant during the study, the subject should notify the investigator. The pregnancy must be reported to Triclinium, and the status of the mother and child after delivery will be obtained and reported, when possible.

Male subjects will be advised of the unknown risk of study vaccine to the unborn fetus and will be advised to use adequate birth control methods (condoms) for the whole the duration of the study.

9.8 Subject Diary and Temperature Monitoring

Subjects will receive, and be instructed in, the operation of a daily adverse event diary and a thermometer to be used during the 7-day post-vaccination diary period after vaccine administration. The daily adverse event diary is a source document and will record solicited adverse events, daily thermometer assessments of body temperature, and any other adverse events. At the safety visits scheduled 7 days after each vaccination the diary card will be collected and reviewed by the principal investigator (or designee) at which time any clinical details required for complete understanding of the information recorded will be obtained. Diaries not brought to the scheduled visit should be obtained before adverse event assessment can be performed. Lost diaries will be reconstructed as possible on a new diary booklet by the subject from memory at the closest clinic visit and labeled as a reconstructed diary. Adverse events obtained from the diary will be recorded and completely assessed on the Adverse Event CRF. Body temperatures below 38º C will not be considered fevers.

Any change to an AE recorded by the subject on the diary card (e.g., the severity level of an event is changed after interviewing the subject) based on the investigator's evaluation of the event must be explained by notation in source documentation.

10 STUDY PAUSE/STOPPING RULES

10.1 General rules for a study pause

The Sponsor will appoint an independent DSMB to monitor the safety of the subjects enrolled in the study. The DSMB will review all serious adverse events reports in real-time and will receive periodic reports of all non-serious adverse events; the DSMB may un-blind any amount of safety information needed to conduct their assessments. The DSMB will determine whether the study should be continued, modified or stopped for safety concerns. Guidelines for making such a decision will be developed by the Sponsor in consultation with the DSMB.

10.2 Premature Discontinuation of the Study

ISS may stop the conduct of the trial at the clinical site for any of the following reasons:

- the site cannot include an adequate number of subjects
- significant and/or persistent non-compliance with the protocol
- careless or premeditated false documentation in the CRFs

- inadequate co-operation with the Sponsor
- non-compliance with ICH GCP, SOPs or the applicable regulatory requirements
- the investigator requests to discontinue the trial
- lack of confidentiality and/or non-compliance with the contractual agreement with the Sponsor.

If the trial is prematurely terminated or suspended, ISS will inform the investigators/institutions, and the regulatory authority and IEC/s, providing the justification for this course of action. All the subjects involved in the study will be informed promptly and appropriate follow-up and if relevant treatment will be assured.

11 DATA RECORDING, MONITORING AND DATA MANAGEMENT

11.1 Data Recording

Case Report Forms

All subject data generated by the study will be recorded on the CRFs provided. All original entries should be initialed and dated by the individual conducting the assessment. The forms will consist of 3-part no-carbon-required paper and should be completed in black ink (hard point pen).

Any correction or deletion should be made by drawing a single line through the entry so that the original entry is still legible. This change must be initialed and dated by the site study coordinator or Investigator. The top 2 pages will be collected by the study monitor. The bottom page should be retained by the Principal Investigator, and must be made available for review by the Sponsor, Institutional Review Board (IRB), or appropriate regulatory agencies.

Any changes made to data after collection of the CRF pages by Data Management will be made through the use of Data Clarification Forms (DCF). Data reported on the CRFs, which are derived from source documents, should be consistent with the source documents or the discrepancies should be explained. Case Report Forms will be considered complete when all missing and/or incorrect data have been resolved.

Source Documentation

Source documents are considered to be all information in original records and certified copies of original records of clinical findings, observations, data or other activities in a clinical study necessary for the reconstruction and evaluation of the study.
Source documents will be maintained for all data collected in the CRFs.
Investigators are required to prepare and maintain adequate and accurate case histories designed to record all observations and other data pertinent to the investigation on each individual entered into the study. Data reported on the Case Report Form (CRF) must be consistent with the source documents.

11.2 Data Monitoring

This trial will be conducted in accordance with the principles of Good Clinical Practice. Before clinical trial initiation, Triclinium personnel will visit the clinical site to:

- determine the adequacy of the site facilities

- discuss with the investigator(s) (and other personnel involved in the study) their responsibilities with regard to protocol adherence, and the responsibilities of Sponsor or its representatives.

During the study, a Clinical Trial Monitor will have regular contacts with clinical site, including visits to:

- provide information and support to the investigator(s)
- confirm that facilities remain acceptable
- confirm that the investigational team is adhering to the protocol, that data are being accurately recorded in the case report forms, and that investigational product accountability checks are being done
- perform source data verification (a cross-check between data recorded in CRF and subjects' medical records and/or other records relevant to the study). This will require direct access to all original data for each subject (e.g. clinical records).

11.3 Data Management

Independent, double-data entry will be performed into an electronic database by two different data capturers. Verification of the data will be performed after a comparison of the two entries. Data validation will include checks of limits, codes, missing data and logical inter- and intra-table consistency. Irresolvable inconsistencies will be referred to the investigational team for clarification. Upon completion of all validation processes, and after a blind review of the data, the database will be "locked". Data will be extracted from the database directly into data files for statistical analyses.

Modifications and updates to the database will be documented in an audit trail. All data management activities will be performed in a clinical data management system that is fully compliant with international and local guidelines and regulations pertaining to data management.

Medical and surgical history, concomitant illnesses and adverse events will be coded using the Medical Dictionary for Regulatory Activities (MedDRA) Version 12 or higher. Previous and concomitant medication taken at any stage during the study will be coded using the WHO Drug Dictionary (WHO-DD version 2011, quarter 1 or higher).

12 STATISTICAL ANALYSIS

12.1 Statistical Program

Statistical analyses will be performed using SAS® (Version 9.2 or higher, SAS Institute, Cary, NC, USA).

12.2 Endpoints

Primary Endpoint Variable

- Induction of anti-Tat antibodies (IgM, IgG or IgA)

Secondary Endpoint Variables

- Physical examination data
- Vital signs data
- Adverse events

- Laboratory safety parameters
- Virology

12.3 Sample Size Determination

To evaluate the humoral immune response to vaccination, "responders" will be defined as those vaccinated subjects that develop an immune response above a specific cut-off level.

If positive response rates are 80% for the Tat vaccine group and 60% for the placebo group, with alpha set at 0.05 (2-tailed), a sample size of 91 per group will have 80% power of showing statistical significance ($p<0.05$). The assumed response rate for the Tat vaccine group is based on the results of phase I and ongoing phase II studies (Fleiss J.L. Tytun A., Ury S.H.K., A simple approximation for calculating sample sizes for comparing independent proportions, Biometrics, 1980).

Sample size was calculated using nQuery Advisor® software, version 6.01.

It is anticipated that approximately 10% of subjects will not complete the study. Therefore, a total of 200 subjects will be enrolled to ensure that 91 subjects per arm complete the study.

12.4 Population for Statistical Evaluation

Two groups of subject populations will be considered for statistical analysis:

- The **immunogenicity population**, representing all randomized subjects who received at least 2 vaccinations.
- The **safety population**, representing all randomized subjects who received at least one administration of Tat.

12.5 Methods

Continuous data will be summarized using descriptive statistics including N, mean, standard deviation, minimum, medium and maximum. Categorical data will be presented using N and %. 95% confidence intervals will be determined for all data.
All statistical analyses will be performed at a 5% significance level and will be 2-tailed.

12.6 Primary Endpoint Analysis

*To evaluate the **humoral immune response** to vaccination, "responders" will be defined as those vaccinated subjects that develop an immune response greater than the following levels:*

- *Anti-Tat IgM titers ≥ 25*
- *Anti-Tat IgG titers ≥ 100*
- *Anti-Tat IgA titers ≥ 25*

The percentage of responders will be compared between active and placebo groups using the Chi-Square test. Additionally, geometric mean antibody titers (GMT) will be calculated and compared between treatment groups by using a t-Test for unpaired data.

12.7 Secondary Endpoint Analysis

Changes from baseline in physical examination results, vital signs data and laboratory parameters will be summarized for both treatment groups.

MedDRA dictionary will be used for coding adverse events. Adverse events will be summarized by system organ class, preferred term, severity and relationship with the

vaccination; the incidence of adverse events “possibly”, “probably” or “certainly” related to vaccination will be computed. All serious adverse events (SAEs) will be summarized by treatment group, subject, gender, age, duration of the event, action taken, investigator’s assessment of causality and outcome.

Change from baseline will be summarized for $CD4^+$ T cells and HIV viral load. Haematology, clinical chemistry and urinalysis results will be graded according to Appendix I and will be presented for both treatment groups.

12.8 Interim analysis

12.8.1 Rationale/reason for interim analysis

Sample size determination is based on hypothetical positive response rates of not less than 80% and not more than 60% in the treatment and placebo group, respectively (Paragraph 12.3). These assumptions stem from information related to vaccine trials and observational studies conducted both in Italy and South Africa. These studies are now completed and have provided additional information. In particular, data from the studies in Italy indicate about 18% of anti-Tat Ab positive subjects on ART vs 28% positive subjects in South Africa (ISS OBS T-004, ClinicalTrials.gov identifier: NCT01359800). In addition, a longitudinal observational study completed in Italy (ISS OBS T-002, ClinicalTrials.gov identifier: NCT01024556) indicate an anti-Tat seroconversion of 9%. Therefore, the cumulative rate of anti-Tat seroconversion in the placebo group of the present study is expected not to exceed 40%, which is lower than what was indicated in the clinical protocol (60%) It is therefore necessary to re-evaluate the sample size upon interim evaluation of the data.

The interim analysis will be conducted only if approved before the 200 participants foreseen by the protocol are enrolled into the study. All participants that have signed the informed consent form and have been vaccinated at least once by the time of the conclusion of the interim analysis will complete the study as per protocol, irrespective of sample size re-evaluation.

12.8.2 Statistical analysis and population

At least 50% of the original planned population will be included in the interim analysis (i.e. at least 100 subjects). At the time of the interim analysis, data for the humoral immune response to Tat will be used to re-calculate the sample size. “Responders” will be defined using the same criteria as described in section 12.6. Data for all subjects who were randomised and who received at least 2 vaccinations will be included in this analysis. No other data will be analysed for the purpose of the interim analysis.

12.8.3 Preserving of blindness

An independent statistical vendor, who will not be involved in the final statistical analysis, will perform the sample size re-calculation.

12.8.4 Timing of interim analysis

The sample size re-calculation will be performed when at least 100 subjects have received at least 2 vaccinations, i.e., have completed at least the Week 8 assessments (Visit 6).

12.8.5 Adjustments to Type 1 error

Since no formal treatment group comparison will be performed on the humoral immune response data, it will not be necessary to adjust for any inflation of the overall Type 1 error rate.

13 ETHICAL AND ADMINISTRATIVE PROCEDURES

13.1 Informed Consent

Preparation of the consent form is the responsibility of the Sponsor with the support of the CRO. The principles of informed consent in the current edition of the Declaration of Helsinki should be implemented. Informed consent will be obtained from each subject in accordance with the Declaration of Helsinki and ICH-GCP prior to performing any protocol-specified procedures.

Informed consent will be documented in writing. The written Subject Information and Informed Consent form will be approved by the IEC. The written consent document will embody the elements of informed consent as described in the Declaration of Helsinki and will also comply with local regulations.

The investigator or the investigator's qualified designee will provide all relevant information in both oral and written form in a way that is understandable to the subject. Ample time and opportunity must be given for the subject to inquire about details of the study. The subject must be informed about the study's purpose including why the subject was selected to participate, study goals, expected benefits and risks, potential risks, and that some potential risks are unforeseeable. The individual must be provided with a description of the procedures and the estimated duration of time required for participation in the study, as well as alternative interventions or courses of treatment, if applicable.

The subject must receive an explanation as to whether any compensation and any medical treatments are available if injury occurs and, if so, what they are; where further information may be obtained, and who to contact in the event of a study-related injury. Individuals must be told who to contact for answers to any questions related to the study.

The subject must be informed that their participation is voluntary and that they are free to withdraw from the study for any reason at any time without penalty or loss of benefits to which they are otherwise entitled. The extent of the confidentiality of subject records must be defined and the subject must be informed that applicable data protection legislation will applies.

The subject must be informed that the study monitor(s), auditor(s), and the applicable regulatory authorities and Ethics Committees will be granted direct access to the subject's original study medical records for verification of protocol-specified procedures and/or data, without violating the confidentiality of the subject to the extent permitted by the applicable laws and regulations.

The subject must be informed that his/her signature on the informed consent form indicates that he/she has decided to participate in the study, having read and discussed the information presented.

The informed consent and any other information provided to the subjects, should be revised whenever important new information becomes available that is relevant to the subject's consent, and should receive IEC approval/ prior to use. The Investigator, or the investigator's qualified designee, should fully inform the subject of all pertinent aspects of the study and of any new information relevant to the subject's willingness to continue participation in the study. This communication should be documented.

The original, signed informed consent form for each subject will be maintained by the investigator as part of the subject's study records. A copy of the signed informed consent form will be provided to each subject.

13.2 Ethical Issues and the Independent Ethics Committee

This study will be conducted in accordance with the protocol and any applicable amendments, the Declaration of Helsinki, Seoul 2008, ICH GCP, July 2002 and the Guidelines for Good Practice in the Conduct of Clinical Trials in Human Participants in South Africa, 2006.

The protocol and informed consent form will be reviewed and approved by the IEC prior to any protocol-specified procedures being conducted.
The investigator will inform the IEC as to the progress of the study on a regular basis, or at minimum, once a year.

All the documents the IEC may need to fulfil its responsibilities, such as the protocol, protocol amendments, information concerning subject recruitment, payment or compensation procedures, etc., will be submitted to the IEC by the investigator. Written, unconditional approval of the study protocol and the informed consent form by the IEC will be in the possession of the Investigator /clinical site staff prior to the conduct of any protocol-specified procedures.

Amendments to the protocol may not be implemented without prior written IEC approval except when necessary to eliminate immediate hazards to a subject/s or when the amendment involves only logistical or administrative aspects of the study. Such logistical or administrative amendments will be submitted to the IEC in writing by the investigator, and a copy of the correspondence to verify the submission will be maintained.

The investigator must inform the IEC of amendments to the informed consent or any other documents previously submitted for review/approval, of any new information that may adversely or otherwise affect the safety of the subjects or the conduct of the study, provide an annual update and/or request for re-approval, and advise the IEC when the study has been completed.

Any documents to be provided to the subject (e.g., information cards, letters from the investigator), and all forms of study advertising (flyers, brochures, print advertisements, radio or television scripts, etc.) must be approved by ISS or its designee prior to the clinical site submitting them to the IEC. Approval from the IEC must be obtained prior to providing the documents to the subject, or utilization of the study advertisement.

13.3 Subjects Data Protection – Direct Access to Source Data

The rights, safety and well-being of the individual trial participants are the most important considerations and should prevail over interests of science and society.

Study personnel involved in conducting this trial will be qualified by education, training, and experience to perform their respective tasks.
This trial will not use the services of study personnel where sanctions have been invoked or where there has been scientific misconduct or fraud (e.g., loss of medical licensure, debarment).

13.4 Insurance, Indemnity and Refunds

Adequate insurance coverage for all subjects to be included in the trial will be supplied by the Sponsor

13.5 Audits and Inspections

Authorized representatives of ISS, Regulatory authorities and Independent Ethics Committees may visit the clinical trial site to perform audits or inspections, including source data verification. The purpose of any audit or inspection is to systematically and independently examine all study related activities and documents in order to determine whether these activities were properly conducted, and data were recorded, analyzed, and accurately reported according to the protocol, Good Clinical Practice (GCP), the guidelines of the International Conference on Harmonisation (ICH), and applicable regulatory requirements. The investigator should notify ISS immediately if contacted by a regulatory agency about an inspection at his/her trial site.

13.6 Training of Study Staff

The principal investigator will maintain training records for all individuals involved in the study (medical, nursing and other staff). He/She will ensure that appropriate training is given to all staff, and that any new information of relevance to the conduct of this study is provided to the staff involved.

14 CASE REPORT FORMS & RECORD RETENTION

Data recorded on source documents will be transcribed onto ISS approved case report forms (CRFs) provided by Triclinium. Completed, original CRFs will be retrieved by Triclinium and a copy of each completed CRF will be retained at the clinical site as part of the study records.
The study will be monitored regularly by Triclinium throughout the study period.

All study records (source documents, signed informed consent forms, copies of CRFs, Regulatory Authority and IEC correspondence and approval letters, study vaccine management records) will be kept secured for a minimum period 2 years after the last approval of a marketing application in an ICH region and until there are no pending or contemplated marketing applications in an ICH region or after the formal discontinuation of the clinical development of the investigational product.
The investigator will ensure that study records are not disposed of or removed from the clinical site without prior notification and approval from ISS. ISS will notify the Investigator when retention of the trial records is no longer required.

If an Investigator withdraws from the study prematurely (e.g., relocation, retirement) the study records will be transferred to a mutually agreed designee (e.g., another Investigator, Ethics Committee). Notice of such transfer will be given in writing to ISS.

15 PUBLICATION OF RESULTS

All information regarding this study or obtained as a result of this study is regarded as confidential; by signature of this protocol, the Investigators agree that the scientific results of this study are the property of ISS.

Unpublished information contained herein, as well as any information received from the Sponsor for the purposes of this study, may not be disclosed to any third party without the prior written approval of the Sponsor. No data may be used for presentation at scientific meetings and/or publication in scientific journals without the prior written authorization from the Sponsor. The investigators and study site staff must send all manuscripts, abstracts, and presentations using data from this study to ISS for review prior to their submission. ISS reserves the right to delete any part or parts of such materials deemed to be confidential or proprietary.

ISS reserves the right to use the results of this trial for scientific applications and/or submissions to Regulatory Authorities. ISS is committed to publication of the results of this study after study conclusion.

16 STUDY TIMETABLE

The enrolment of the first subject is planned to take place in October 2011 and will continue until the last treatment number has been allocated. The investigators are expected to make every reasonable effort to recruit suitable subjects into the study. If the enrolment rate is low, or if a significant number of protocol violators are recruited or if no subjects are recruited within a reasonable period, ISS reserves the right to prematurely close enrolment or pause or terminate the study.

17 AMENDMENTS TO THE PROTOCOL

17.1 Summary of changes from Protocol Version 1.0 to Version 2.0

Section	Version 1.0	Version 2.0
**Cover page**	**Change/Rationale:**<br>Updated to amend the protocol version	
	**Protocol Version**:<br>ISS T-003 Protocol Final Version 1.0, 24-June-2010	**Protocol Version**:<br>ISS T-003 Protocol Final Version 2.0, 23-March-2011
**Protocol Synopsis**	**Change/Rationale:**<br>Amended to correspond to the sample size justification which is based only on humoral immune response; cellular immune response will be evaluated as a secondary and not a primary immunogenicity variable, and also to correct an error 'and/or' changed to 'and'.	
	**Primary Immunogenicity Variables**<br>Induction of anti-Tat antibodies (IgM, IgG or IgA) as specific anti-Tat humoral immune response;<br>Induction or increase of lymphoproliferation (CFSE staining) and/or in vitro γIFN, IL-4, IL-2 production (Elispot) in response to Tat, as specific anti-Tat cellular immune response.<br>**Safety Variables**<br>Adverse events, including any significant changes in hematological/biochemical/ coagulation and urinalysis laboratory parameters.<br>Final analysis will be performed after all subjects have completed the study, the database has been locked, and the study has been unblinded.	**Primary Immunogenicity Variable**<br>• Induction of anti-Tat antibodies (IgM, IgG or IgA) as specific anti-Tat humoral immune response.<br>**Secondary Variables**<br>• Induction or increase of lymphoproliferation (CFSE staining) and in vitro γIFN, IL-4, IL-2 production (Elispot) in response to Tat, as specific anti-Tat cellular immune response.<br>• Adverse events, including any significant changes in hematological/biochemical/ coagulation and urinalysis laboratory parameters.<br>Final analysis will be performed after all subjects have completed the study, the database has been locked, and the study has been unblinded.
**5.1.1 Investigational Product Preparation**	**Change/Rationale:**<br>Amended to allow for the vaccine dose to be administered via 4 x 0.5 ml injections rather than 2 x 1.0 ml injections; administering a smaller volume intradermally is considered to be more comfortable for the participant and technically more reliable.	
	Immediately prior to the administration of the vaccine (≤ 40 minutes before	Immediately prior to the administration of the vaccine (≤ 40 minutes before administration), the

	administration), the vial contents should be allowed to thaw and then diluted with sterile water according to the following process:<br>1. Place the vial containing the vaccine (0.5 ml) on ice and protected from light.<br>2. Add 1.5 ml of sterile water for injection to the vial containing the vaccine. Keep the vial protected from light.<br>3. Mix by gently agitating the vial to obtain a homogeneous solution (2.0 ml total volume).<br>4. Prepare the syringe for the first injection: draw up 1.0 ml of the vaccine preparation.<br>5. Prepare the syringe for the second injection: draw up 1.0 ml of the vaccine preparation.<br>6. Administer the vaccine by two separate intradermal injections into the deltoids of the right and left upper arms (1.0 ml into the right arm and 1.0 ml into the left arm).	vial contents should be allowed to thaw and then diluted with sterile water according to the following process:<br>1. Place the vial containing the vaccine (0.5 ml) on ice and protected from light.<br>2. Add 1.5 ml of sterile water for injection to the vial containing the vaccine. Keep the vial protected from light.<br>3. Mix by gently agitating the vial to obtain a homogeneous solution (2.0 ml total volume).<br>4. Prepare the syringe for the first injection: draw up 1.0 ml of the vaccine preparation.<br>5. Prepare the syringe for the second injection: draw up 1.0 ml of the vaccine preparation.<br>6. Administer the vaccine by four separate intradermal injections into the deltoids of the right and left upper arms (2 x 0.5 ml into two separate sites on the right arm and 2 x 0.5 ml into two separate sites on the left arm).
**5.5 Investigational Product Storage and Accountability**	**Change/Rationale:**<br>Amended to clarify that only un-used vials of investigational product will be returned to the Sponsor at the end of the study, and that used vials will be destroyed at site according to the site's standard operating procedures.	
	It is the investigator/clinical institution's responsibility to make all reasonable efforts to ensure that:<br>• IP delivered is correctly received (the received product should be checked against the shipment record, and the acknowledgement of receipt form for the IP should be completed appropriately and faxed back to the shipper immediately upon receipt).<br>• The investigational product is stored under the required conditions in a secure facility to which access is restricted to	It is the investigator/clinical institution's responsibility to make all reasonable efforts to ensure that:<br>• IP delivered is correctly received (the received product should be checked against the shipment record, and the acknowledgement of receipt form for the IP should be completed appropriately and faxed back to the shipper immediately upon receipt).<br>• The investigational product is stored under the required conditions in a secure facility to which access is restricted to authorized individuals only.

	authorized individuals only.<br>• The investigational product is only administered to eligible subjects in accordance with the protocol requirements<br>• All unused investigational product and residual used vials are returned to the Sponsor at the end of the study conduct.<br><br>Temperature logs of the storage facility will be maintained by the clinical site, and will be checked by the CRA at each monitoring visit.<br><br>The investigational product will be administered to subjects by authorized personnel only (Principal Investigator or designee). A drug inventory will be maintained and will include details of all received and returned investigational product. Drug accountability forms will also be maintained to detail all dispensed and administered vaccines and the subjects to whom they were administered.<br>At the conclusion of the clinical conduct of the study, all investigational product/placebo supplies (including used, unused, and partially used vials), will be returned to the Sponsor.	• The investigational product is only administered to eligible subjects in accordance with the protocol requirements<br>• All unused investigational product are returned to the Sponsor at the end of the study conduct.<br>• All used or partially used vials are destroyed according to the site's operating procedures.<br><br>Temperature logs of the storage facility will be maintained by the clinical site, and will be checked by the CRA at each monitoring visit.<br><br>The investigational product will be administered to subjects by authorized personnel only (Principal Investigator or designee). A drug inventory will be maintained and will include details of all received, returned and destroyed investigational product. Drug accountability forms will also be maintained to detail all dispensed and administered vaccines and the subjects to whom they were administered.<br>At the conclusion of the clinical conduct of the study, all unused investigational product/placebo supplies will be returned to the Sponsor.
**6.6.2 Standard Laboratory Assessments**	**Change/Rationale:**<br>Amended to correct error in urinalysis dipstick test parameters: 'nitrate' changed to 'nitrites'	
	**Urinalysis**:<br>Dipstick determination of Protein, Glucose, Nitrate, Albumin, Leukocytes and Red blood cells	**Urinalysis**:<br>Dipstick determination of Protein, Glucose, Nitrites, Albumin, Leukocytes and Red blood cells
**6.6.3 Immunological Evaluations**	**Change/Rationale:**<br>Amended to correspond to the sample size justification which is based only on humoral immune response; cellular immune response will be evaluated as a secondary and not a primary immunogenicity variable.	

	**First line immunology testing**<br>Assessment of anti-Tat humoral immune response:<br>• Determination of IgM, IgG and IgA anti-Tat antibodies in sera<br>• Titration of IgM, IgG and IgA anti-Tat antibodies<br>Assessment of anti-Tat cellular immune response:<br>• Lymphoproliferative response to Tat (CFSE staining)<br>• *In vitro* γIFN, IL-4 and IL-2 production in response to Tat (Elispot).<br><br>**Second line immunology testing**<br>The following parameters will be determined, depending on the availability of residual specimens (PBMC, serum, plasma):<br>• Characterization of lymphocyte subsets (CD3, CD4, CD8, CD16, CD56, CD19)<br>• Anti-Tat IgG (IgG1, IgG2, IgG3, IgG4) subclasses<br>• Epitope mapping of IgM and IgG anti-Tat antibodies<br>• Neutralization of Tat activity by in vitro assays (rescue inhibition assay)<br>• Anti-HIV regulatory and structural proteins antibodies<br>• Antibody-mediated cellular cytotoxicity (ADCC)<br>• Inhibition of Tat uptake by MDDCs<br>• Neutralization of primary isolates (all clades)<br>• Anti-CCR5 antibodies<br>• Anti-CD4 antibodies<br>• Lymphoproliferative response to mitogens and recall antigens<br>• Lymphoproliferative response to HIV-1 Env (CFSE staining)<br>• *In vitro* γIFN, IL-4 and IL-2 production in response to Env (ICS/Elispot)	**First line immunology testing**<br>Assessment of anti-Tat humoral immune response:<br>• Determination of IgM, IgG and IgA anti-Tat antibodies in sera<br>• Titration of IgM, IgG and IgA anti-Tat antibodies<br><br>**Second line immunology testing**<br>Assessment of anti-Tat cellular immune response:<br>• Lymphoproliferative response to Tat (CFSE staining)<br>• *In vitro* γIFN, IL-4 and IL-2 production in response to Tat (Elispot).<br>The following parameters will be determined, depending on the availability of residual specimens (PBMC, serum, plasma):<br>• Characterization of lymphocyte subsets (CD3, CD4, CD8, CD16, CD56, CD19)<br>• Anti-Tat IgG (IgG1, IgG2, IgG3, IgG4) subclasses<br>• Epitope mapping of IgM and IgG anti-Tat antibodies<br>• Neutralization of Tat activity by in vitro assays (rescue inhibition assay)<br>• Anti-HIV regulatory and structural proteins antibodies<br>• Antibody-mediated cellular cytotoxicity (ADCC)<br>• Inhibition of Tat uptake by MDDCs<br>• Neutralization of primary isolates (all clades)<br>• Anti-CCR5 antibodies<br>• Anti-CD4 antibodies<br>• Lymphoproliferative response to mitogens and recall antigens<br>• Lymphoproliferative response to HIV-1 Env (CFSE staining)<br>• *In vitro* γIFN, IL-4 and IL-2 production in response to Env (ICS/Elispot)<br>• B cells phenotype (naïve and memory) and antigen specific

	• B cells phenotype (naïve and memory) and antigen specific antibodies<br>• Phenotype and functional characterization of regulatory T cells<br>• Intracellular PBMC staining for granzyme, perforin, cytokines and chemokines<br>• Analysis of Th1 and Th2 cytokines in sera and in PBMC supernatants<br>• Analysis of chemokines in sera and in PBMC supernatants<br>• Lymphocytes spontaneous cell death and PBMC cell viability<br>• B cell cloning<br>• Functional and molecular characterization of clono-specific antibodies<br>• Serum/plasma determination of soluble CD4<br>• Serum/plasma determination of phenotypic and biochemical markers of immune activation (CD38 expression, neopterin, β2-microglobulin, C reactive protein, total immunoglobulin, sCD27, sCD14, LPS and IgM against endotoxin core antigen by ELISA)<br>• HLA typing	antibodies<br>• Phenotype and functional characterization of regulatory T cells<br>• Intracellular PBMC staining for granzyme, perforin, cytokines and chemokines<br>• Analysis of Th1 and Th2 cytokines in sera and in PBMC supernatants<br>• Analysis of chemokines in sera and in PBMC supernatants<br>• Lymphocytes spontaneous cell death and PBMC cell viability<br>• B cell cloning<br>• Functional and molecular characterization of clono-specific antibodies<br>• Serum/plasma determination of soluble CD4<br>• Serum/plasma determination of phenotypic and biochemical markers of immune activation (CD38 expression, neopterin, β2-microglobulin, C reactive protein, total immunoglobulin, sCD27, sCD14, LPS and IgM against endotoxin core antigen by ELISA)<br>• HLA typing
**6.6.4 Virology Evaluations**	**Change/Rationale:**<br>Amended to clarify that not all virology evaluations will be performed by a Core Laboratory; the evaluation of HIV-1 Plasma Viraemia will take place at the relevant local safety laboratory.	
	Virology evaluations will be performed according to "first line" and a "second line" laboratory testing by the designated Core Laboratory to determine............	Virology evaluations will be performed according to "first line" and a "second line" laboratory testing by the designated Laboratory to determine..........

<table>
<tr><td rowspan="2">6.6.5
Sample Collection, Storage and Shipment</td><td colspan="2">Change/Rationale:
Amended to correct the volumes of blood required for the safety and immunology assessments</td></tr>
<tr><td>The volume of blood to be collected at each study visit will vary depending on the specific analyses to be performed at that visit. Approximate volumes for the assessments are 40 ml for immunology and virology evaluations and 10 ml for assessing safety parameters. The total volume that can be collected at any one visit is ± 50 ml as per the following table:

Immunology/Virology: 40ml
Safety: Clinical Chemistry: 7ml
Safety: Haematology: 3ml
Total: 50ml</td><td>The volume of blood to be collected at each study visit will vary depending on the specific analyses to be performed at that visit. Approximate volumes for the assessments are 31 ml for immunology and virology evaluations and 19 ml for assessing safety parameters. The total volume that can be collected at any one visit is ± 50 ml as per the following table:

Immunology/Virology: 31ml
Safety: Clinical Chemistry: 5ml
Safety: Haematology: 14ml
Total: 50ml</td></tr>
<tr><td>6.7
Schedule of Study Procedures</td><td colspan="2">Change/Rationale:
Amended to add ARV adherence counselling at the following visits: Screening, Day 0 Post-vaccination, Day 28 Post-vaccination, Day 56 Post-vaccination, Day 84, Day 112, Day 140 & Day 168.
Counselling on ARV adherence, risk reduction and the prevention of pregnancy was added to the Day 336 procedures.</td></tr>
<tr><td rowspan="2">6.7
Day 0 Post-vaccination procedures</td><td colspan="2">Change/Rationale:
Amended to correct the visit window for the first vaccination safety follow-up visit</td></tr>
<tr><td>The next visit will be scheduled for 7 days (28 ± 1 day) later.</td><td>The next visit will be scheduled for 7 days (± 1 day) later.</td></tr>
<tr><td rowspan="2">8.
Endpoints

8.1
Primary Endpoint Variables and Measurements</td><td colspan="2">Change/Rationale:
Amended to correspond to the move of cellular immune response from a primary to a secondary immunogenicity variable.</td></tr>
<tr><td>In order to evaluate the immunogenicity of Tat protein in the study population, the following primary endpoints will be considered:

1. Induction of specific anti-Tat humoral immune response in terms of IgM, IgG or IgA anti-Tat antibodies.
2. Induction or increase of anti-Tat cellular-mediated immune</td><td>In order to evaluate the immunogenicity of Tat protein in the study population, the following primary endpoint will be considered:

• Induction of specific anti-Tat humoral immune response in terms of IgM, IgG or IgA anti-Tat antibodies.</td></tr>
</table>

	response, in terms of lymphoproliferative response to Tat (CFSE staining), in vitro γIFN, IL-4, IL-2 production (Elispot) by peripheral blood mononuclear cells (PBMC) in response to Tat.<br><br>The induction, magnitude and persistence of the humoral and cellular immune responses to the administered vaccination schedule will be compared between the active and placebo groups.<br><br>The induction of the anti-Tat specific **humoral immune response** will be evaluated in the active as compared to the placebo group as follows:<br>1. Percentage of "responders", defined as those subjects that develop an immune response greater than the following levels:<br>• Anti-Tat IgM titers ≥ 25<br>• Anti-Tat IgG titers ≥ 100<br>• Anti-Tat IgA titers ≥ 25.<br>2. Geometric mean antibody titers (GMT) induced by vaccination.<br><br>The induction of the anti-Tat specific **cellular immune response** will be evaluated in the active as compared to the placebo group as follows:<br>1. Percentage of subjects with a positive cellular immune response to the vaccination schedule (responders). Responders will be defined as those vaccinated subjects that develop immune responses, at one or more time points after vaccination, above the following thresholds:<br>• γIFN production ≥ 3 fold	The induction, magnitude and persistence of the humoral response to the administered vaccination schedule will be compared between the active and placebo groups.<br><br>The induction of the anti-Tat specific **humoral immune response** will be evaluated in the active as compared to the placebo group as follows:<br>1. Percentage of "responders", defined as those subjects that develop an immune response greater than the following levels:<br>• Anti-Tat IgM titers ≥ 25<br>• Anti-Tat IgG titers ≥ 100<br>• Anti-Tat IgA titers ≥ 25<br>2. Geometric mean antibody titers (GMT) induced by vaccination.

	and 30 spots/10^6 cells<br>• IL-2 production ≥ 3 fold<br>• IL-4 production ≥ 3 fold<br>• Lymphoproliferation fold increase ≥ 2<br>2. Increase of anti-Tat cellular-mediated immune response, evaluated in terms of fold/spots/proliferation index.	
**8.2 Secondary Endpoint Variables and Measurements**	In order to evaluate the safety of the Tat protein vaccination schedule in the study population, the following secondary endpoints will be considered and compared between active and placebo groups:<br><br>• Changes in physical examination findings from baseline;<br>• Changes in vital signs from baseline;<br>• Adverse events (including local and systemic reactions to the vaccination schedule occurring during the course of the study);<br>• Changes in standard laboratory safety parameters from baseline	In order to evaluate the safety of the Tat protein vaccination schedule in the study population, the following secondary endpoints will be considered and compared between active and placebo groups:<br><br>• Induction or increase of anti-Tat cellular-mediated immune response, in terms of lymphoproliferative response to Tat (CFSE staining), in vitro γIFN, IL-4, IL-2 production (Elispot) by peripheral blood mononuclear cells (PBMC) in response to Tat.<br>• Changes in physical examination findings from baseline;<br>• Changes in vital signs from baseline;<br>• Adverse events (including local and systemic reactions to the vaccination schedule occurring during the course of the study);<br>• Changes in standard laboratory safety parameters from baseline<br><br>The induction of the anti-Tat specific **cellular immune response** will be evaluated in the active as compared to the placebo group as follows:<br>1. Percentage of subjects with a positive cellular immune response to the vaccination schedule (responders). Responders will be defined as those vaccinated subjects that develop immune responses, at one or more time

		points after vaccination, above the following thresholds:<br>• γIFN production ≥ 3 fold and 30 spots/10^6 cells<br>• IL-2 production ≥ 3 fold<br>• IL-4 production ≥ 3 fold<br>• Lymphoproliferation fold increase ≥ 2<br>2. Increase of anti-Tat cellular-mediated immune response, evaluated in terms of fold/spots/proliferation index.
**9.1.1.1 Study Vaccine Pausing Rules for the Principal Investigator**	**Change/Rationale:**<br>Amended to correct an error in the causality grades and clarification of the lines of communication and reporting.	
	If the principal investigator determines that a SUSAR OR a serious adverse event OR a Toxicity Grade 4 event OR an adverse event pattern of concern that is judged to be CERTAINLY, PROBABLY or DEFINITELY related to study vaccine has occurred, the principal investigator will pause administration of study vaccine in the trial. If the principal investigator pauses study vaccine administration he or she will record this in a memorandum to the study file and notify the sponsor.	If the principal investigator determines that a SUSAR OR a serious adverse event OR a Toxicity Grade 4 event OR an adverse event pattern of concern that is judged to be POSSIBLY, PROBABLY or CERTAINLY related to study vaccine has occurred, the principal investigator will pause administration of study vaccine in the trial. If the principal investigator pauses study vaccine administration he or she will record this in a memorandum to the study file and notify the sponsor and Local Medical Monitor.
**9.1.2.1.1 Study Vaccine Pausing Rules for the Local Medical Monitor**	**Change/Rationale:**<br>Amended to correct an error in the causality grades.	
	If the local medical monitor determines that a SUSAR OR a serious adverse event OR a Toxicity Grade 4 event OR an adverse event pattern of concern that is judged to be CERTAINLY, PROBABLY or DEFINITELY related to study vaccine has occurred, the local medical monitor will pause administration of study vaccine in the trial.	If the local medical monitor determines that a SUSAR OR a serious adverse event OR a Toxicity Grade 4 event OR an adverse event pattern of concern that is judged to be POSSIBLY, PROBABLY or CERTAINLY related to study vaccine has occurred, the local medical monitor will pause administration of study vaccine in the trial.

<table>
<tr><td rowspan="2">9.2.1
Adverse Event (AE)</td><td colspan="2">Change/Rationale:
Amended to clarify that only clinically significant changes in laboratory safety parameters will be reported as adverse events.</td></tr>
<tr><td>Adverse events include but are not limited to: adverse changes from baseline that represent increases in toxicity grade according to the Toxicity Table, adverse changes in the general condition of the subject, signs and symptoms noted by the subject, concomitant disease with onset or increased severity after study vaccine administration, and changes in laboratory safety parameters occurring after study vaccine administration.</td><td>Adverse events include but are not limited to: adverse changes from baseline that represent increases in toxicity grade according to the Toxicity Table, adverse changes in the general condition of the subject, signs and symptoms noted by the subject, concomitant disease with onset or increased severity after study vaccine administration, and clinically significant changes in laboratory safety parameters occurring after study vaccine administration.</td></tr>
<tr><td rowspan="2">9.2.4
Serious Adverse Drug Reaction (SADR)</td><td colspan="2">Change/Rationale:
Amended to include the definition of a SUSAR</td></tr>
<tr><td>A SADR is a SAE suspected to be causally related to the investigational product.</td><td>A SADR is a SAE suspected to be causally related to the investigational product; a SUSAR is a Serious Unexpected Suspected Adverse Drug Reaction or unexpected SADR.</td></tr>
<tr><td rowspan="2">9.4
Adverse Event treatment, Follow-Up and Outcome</td><td colspan="2">Change/Rationale:
Amended to clarify that only clinically significant changes in laboratory safety parameters will be reported as adverse events.</td></tr>
<tr><td>The investigator will continue follow-up on adverse events, including laboratory abnormalities until the event has resolved, is otherwise satisfactorily explained, or the subject completes the study. If not reported earlier, the outcome of adverse events will be determined when the subject completes the study.</td><td>The investigator will continue follow-up on adverse events, including clinically significant laboratory abnormalities until the event has resolved, is otherwise satisfactorily explained, or the subject completes the study. If not reported earlier, the outcome of adverse events will be determined when the subject completes the study.</td></tr>
<tr><td rowspan="2">9.5
Reporting of Serious Adverse Events/Serious Adverse Drug Reactions</td><td colspan="2">Change/Rationale:
Amended to clarify that ALL SAEs that are suspected to be related to the study vaccine would be subject to immediate telephonic reporting to the local Medical Monitor, and in addition to clarify the subsequent lines of communication and reporting.</td></tr>
<tr><td>Fatal or life-threatening serious adverse events that the investigator suspects are related</td><td>All serious adverse events that the investigator suspects are related to the study vaccine (SUSARs) should</td></tr>
</table>

	to the study vaccine should be **telephoned to the local medical monitor immediately upon the investigator's awareness of the event.** If the local medical monitor is required by the protocol or chooses to suspend enrolment she/he shall immediately create a written memorandum for record to the study file and telephonically notify the sponsor and Triclinium of this act.	be **telephoned to the local medical monitor immediately upon the investigator's awareness of the event.** Following assessment of the information, if the local medical monitor is required by the protocol or chooses to suspend enrolment she/he shall immediately create a written memorandum for record to the study file and telephonically notify the Principal Investigator, Sponsor and Triclinium of this act.
	**Change/Rationale:**<br>Amended to clarify that the investigative site is responsible for the notification of all SAEs to the relevant Ethics Committee.	
	ISS has authorized Triclinium to execute its responsibilities for safety report submission to the appropriate regulatory authorities within specific time periods of being notified of the event. ISS will notify the DSMB of all SAEs within three working days of becoming aware of an event and will provide all follow-up information in a timely manner.	ISS has authorized Triclinium to execute its responsibilities for safety report submission to the appropriate regulatory authorities within specific time periods of being notified of the event; the investigative site is responsible for notification of all SAE reports to the relevant Ethics Committee within the appropriate reporting time periods. ISS will notify the DSMB of all SAEs within three working days of becoming aware of an event and will provide all follow-up information in a timely manner.
**9.6<br>Other Events Requiring Immediate Reporting**	**Change/Rationale:**<br>Amended to clarify the lines of communication and reporting.	
	The investigator must report the following events by faxing the appropriate form to the local medical monitor within 24 hours of becoming aware of the event:	The investigator must report the following events by faxing the appropriate form to the local medical monitor and Triclinium within 24 hours of becoming aware of the event; Triclinium will forward the appropriate form to ISS:
**12.2<br>Endpoints**	**Change/Rationale:**<br>Amended to correspond to the move of cellular immune response from a primary to a secondary immunogenicity variable.	
	**Primary Endpoint Variables**<br>• Induction of anti-Tat antibodies (IgM, IgG or IgA),	**Primary Endpoint Variable**<br>• Induction of anti-Tat antibodies (IgM, IgG or IgA)

	• lymphoproliferative response to Tat (CFSE staining),<br>• in vitro γIFN, IL-4, IL-2 production against Tat.<br><br>**Secondary Endpoint Variables**<br>• Physical examination data,<br>• Vital signs data,<br>• Adverse events,<br>• Laboratory safety parameters,<br>• Virology	**Secondary Endpoint Variables**<br>• lymphoproliferative response to Tat (CFSE staining)<br>• in vitro γIFN, IL-4, IL-2 production against Tat<br>• Physical examination data<br>• Vital signs data<br>• Adverse events<br>• Laboratory safety parameters<br>• Virology
**12.6 Primary Endpoint Analysis**	**Change/Rationale:**<br>Amended to correspond to the move of cellular immune response from a primary to a secondary immunogenicity variable.	
	To evaluate the **humoral immune response** to vaccination, “responders” will be defined as those vaccinated subjects that develop an immune response greater than the following levels:<br>• Anti-Tat IgM titers ≥ 25<br>• Anti-Tat IgG titers ≥ 100<br>• Anti-Tat IgA titers ≥ 25<br>The percentage of responders will be compared between active and placebo groups using the Chi-Square test. Additionally, geometric mean antibody titers (GMT) will be calculated and compared between treatment groups by using a t-Test for unpaired data.<br><br>To evaluate the **cellular immune response** to vaccination, “responders” will be defined as vaccinated subjects that develop an immune response at one or more time points after vaccination and above the following levels:<br>• γIFN production ≥3 fold and 30 spots/10^6 cells,<br>• IL-2 production ≥ 3 fold<br>• IL-4 production ≥3 fold<br>• Lymphoproliferation fold increase ≥ 2<br>The percentage of responders will be compared between active and placebo groups using the Chi-	To evaluate the **humoral immune response** to vaccination, “responders” will be defined as those vaccinated subjects that develop an immune response greater than the following levels:<br>• Anti-Tat IgM titers ≥ 25<br>• Anti-Tat IgG titers ≥ 100<br>• Anti-Tat IgA titers ≥ 25<br>The percentage of responders will be compared between active and placebo groups using the Chi-Square test. Additionally, geometric mean antibody titers (GMT) will be calculated and compared between treatment groups by using a t-Test for unpaired data.

	Square test. Increases of anti-Tat cellular-mediated immune response will also be evaluated in terms of fold/spots/proliferation index. Multivariate regression models will be used to test for any dependency of variables.	
**12.7 Secondary Endpoint Analysis**	**Change/Rationale:**<br>Amended to correspond to the move of cellular immune response from a primary to a secondary immunogenicity variable, and in addition clarification of the adverse event causality terms.	
	Change from baseline in physical examination results, vital signs data and laboratory parameters will be summarized for both treatment groups.<br><br>MedDRA dictionary will be used for coding adverse events. Adverse events will be summarized by system organ class, preferred term, severity and relationship with the vaccination; the incidence of adverse events "possibly", "likely" or "clearly" related to vaccination will be computed. All serious adverse events (SAEs) will be summarized by treatment group, subject, gender, age, duration of the event, action taken, investigator's assessment of causality and outcome.<br><br>Change from baseline will be summarized for $CD4^+$ T cells and HIV viral load. Haematology, clinical chemistry and urinalysis results will be graded according to Appendix I and will be presented for both treatment groups.	To evaluate the **cellular immune response** to vaccination, "responders" will be defined as vaccinated subjects that develop an immune response at one or more time points after vaccination and above the following levels:<br>• γIFN production ≥3 fold and 30 spots/10^6 cells<br>• IL-2 production ≥ 3 fold<br>• IL-4 production ≥3 fold<br>• Lymphoproliferation fold increase ≥ 2<br>The percentage of responders will be compared between active and placebo groups using the Chi-Square test. Increases of anti-Tat cellular-mediated immune response will also be evaluated in terms of fold/spots/proliferation index. Multivariate regression models will be used to test for any dependency of variables.<br><br>Change from baseline in physical examination results, vital signs data and laboratory parameters will be summarized for both treatment groups.<br><br>MedDRA dictionary will be used for coding adverse events. Adverse events will be summarized by system organ class, preferred term, severity and relationship with the vaccination; the incidence of adverse events "possibly",

		"probably" or "certainly" related to vaccination will be computed. All serious adverse events (SAEs) will be summarized by treatment group, subject, gender, age, duration of the event, action taken, investigator's assessment of causality and outcome.<br><br>Change from baseline will be summarized for $CD4^{+}$ T cells and HIV viral load. Haematology, clinical chemistry and urinalysis results will be graded according to Appendix I and will be presented for both treatment groups.
**Section 17: Amendments to the Protocol**	**Change/Rationale:**<br>New section added to summarize protocol changes. As a consequence of this, sections numbered previously as 17, 18, 19, 20, 21 and 22 are now numbered 18, 19, 20, 21, 22 and 23.	
**Appendix ll. Schedule of Protocol Visits and Procedures**	**Change/Rationale:**<br>Amended to add ARV adherence counselling at the following visits: Screening, Day 0 Post-vaccination, Day 28 Post-vaccination, Day 56 Post-vaccination, Day 84, Day 112, Day 140, Day 168 & Day 336.	

17.2 Summary of changes from Protocol Version 2.0 to Version 3.0

Section	Version 2.0	Version 3.0
**Cover page**	**Change/Rationale:**<br>Updated to amend the protocol version	
	**Protocol Version**:<br>ISS T-003 Protocol Final Version 2.0, 23-March-2011	**Protocol Version**:<br>ISS T-003 Protocol Final Version 3.0, 29-July-2011
**1 Protocol Synopsis**	**Change/Rationale:**<br>Amended to allow for the change in estimated study start date.	
	**Planned Study Dates**:<br>24 months. Study start is estimated to be January 2011.	**Planned Study Dates**:<br>24 months. Study start is estimated to be October 2011.
	**Change/Rationale:**<br>This amendment is based on the results of the observational study (Protocol ISS OBS T-004) conducted recently in the study area.<br>An upper viral load cut-off value of 400 copies/ml is still compatible with effective HAART and good adherence to therapy.<br>In addition as it is now known that regular monitoring of the CD4 T cells and viral load by local ARV clinics is performed on average every 12 months, the window period for previous results has been extended to 12 months instead of six months.	
	**Inclusion Criteria:**<br>4. Chronically suppressed HIV-1 infection as indicated by a HIV-1 plasma viraemia < 50 copies/ml and a CD4+ T cell count ≥ 200 cells/µl at screening, and documented at least once during the 6 month period prior to screening, irrespective of the pre-ARV CD4 nadir.	**Inclusion Criteria:**<br>4. Chronically suppressed HIV-1 infection as indicated by a HIV-1 plasma viraemia < 400 copies/ml and a CD4+ T cell count ≥ 200 cells/µl at screening, and documented at least once during the 12 month period prior to screening, irrespective of the pre-ARV CD4 nadir.
	**Change/Rationale:**<br>Amended to correct an error with respect to cellular immune response being included as a secondary endpoint; the statistical analysis of safety data will be the only secondary endpoint.	
	**Statistical Analyses**<br>**Secondary Variables:**<br>• Induction or increase of lymphoproliferation (CFSE staining) and in vitro γIFN, IL-4, IL-2 production (Elispot) in response to Tat, as specific	**Statistical Analyses**<br>**Secondary Variables:**<br>Adverse events, including any significant changes in hematological/biochemical/coagulation and urinalysis laboratory parameters.

	anti-Tat cellular immune response.<br>• Adverse events, including any significant changes in hematological/biochemical/coagulation and urinalysis laboratory parameters.<br><br>Final analysis will be performed after all subjects have completed the study, the database has been locked, and the study has been un-blinded.	Final analysis will be performed after all subjects have completed the study, the database has been locked, and the study has been un-blinded.
**2 Introduction and Rationale**	**Change/Rationale:**<br>As per inclusion criterion # 4 above.	
	This Phase II study is directed at evaluating the immunogenicity (as a primary end-point) and the safety (as a secondary end-point), of a therapeutic, recombinant, biologically active HIV-1 Tat vaccine in HIV-1 infected, anti-Tat antibody negative, ARV-treated adult volunteers with chronically suppressed HIV-1 infection as indicated by a HIV-1 plasma viraemia < 50 copies/ml and a CD4+ T cell count ≥ 200 cells/µl, at screening and documented at least once during the 6 month period prior to screening, irrespective of the pre-ARV CD4 nadir.	This Phase II study is directed at evaluating the immunogenicity (as a primary end-point) and the safety (as a secondary end-point), of a therapeutic, recombinant, biologically active HIV-1 Tat vaccine in HIV-1 infected, anti-Tat antibody negative, ARV-treated adult volunteers with chronically suppressed HIV-1 infection as indicated by a HIV-1 plasma viraemia < 400 copies/ml and a CD4+ T cell count ≥ 200 cells/µl, at screening and documented at least once during the 12 month period prior to screening, irrespective of the pre-ARV CD4 nadir.
**2.1 Background**	**Change/Rationale:**<br>Several updates essentially to note that<br>1. preminary results are now available from 87 participants enrolled in the ongoing ISS T-002 trial in Italy,<br>**2.** the Ensoli B et al manuscript previouly noted as having been submitted, was published in PloS ONE in 2010.	
	In addition the following paragraph was inserted:<br><br>Furthermore these results have been confirmed up to 2 years after the first immunization. Based on these results, and in view of the urgency to improve HIV treatment particularly in individuals with a more advanced immune deterioration, the Investigators and the Data Safety Monitoring Board (DSMB) endorsed an amendment that has been approved by the Ethical Committees. This amendment extended the enrolment criteria to include more immune compromised individuals (CD4+ T cell counts ≥	

	200 cells/µl, with any CD4 nadir) and expanded the total sample size from 128 to 160 volunteers. The enrolment was recently completed and follow-up of the subjects is continuing.	
**5.2**<br>**Study Duration**	**Change/Rationale:**<br>Amended to allow for the change in estimated study start date.	
	Subjects will be screened for up to 21 days prior to treatment initiation, and will be followed for 48 weeks post first vaccination. Study start is estimated to be January 2011.	Subjects will be screened for up to 21 days prior to treatment initiation, and will be followed for 48 weeks post first vaccination. Study start is estimated to be October 2011.
**6.1**<br>**Inclusion Criteria**	**Change/Rationale:**<br>As per above.	
	**Inclusion Criteria:**<br>4. Chronically suppressed HIV-1 infection as indicated by a HIV-1 plasma viraemia < 50 copies/ml and a CD4+ T cell count ≥ 200 cells/µl at screening, and documented at least once during the 6 month period prior to screening, irrespective of the pre-ARV CD4 nadir.	**Inclusion Criteria:**<br>4. Chronically suppressed HIV-1 infection as indicated by a HIV-1 plasma viraemia < 400 copies/ml and a CD4+ T cell count ≥ 200 cells/µl at screening, and documented at least once during the 12 month period prior to screening, irrespective of the pre-ARV CD4 nadir.
**6.5**<br>**Blinding/ Unblinding**	**Change/Rationale:**<br>To clarify that the DSMB will also be notified of any emergency un-blinding at site.	
	The sponsor and CRO will be informed immediately of any emergency un-blinding that occurs at the site. This will be detailed in the Clinical Study Report.	The sponsor and CRO will be informed immediately of any emergency un-blinding that occurs at the site. This information will be communicated to the DSMB and will be detailed in the Clinical Study Report.
**6.6.4**<br>**Virology Evaluations**	**Change/Rationale:**<br>To delete the possible detection of antibodies to several co-infections and to rather include the possible testing for these co-infections, the analysis of HPV infection in females at 3 time points and to include the possible analysis of antiretroviral drug concentrations.	
	**Second line virology testing**<br>The following parameters will be determined, depending on the availability of residual specimens (PBMC, serum, plasma):<br>• HIV-1 sequencing and	**Second line virology testing**<br>The following parameters will be determined, depending on the availability of residual specimens (PBMC, serum, plasma):<br>• HIV-1 sequencing and

<table>
<tr><td></td><td>phylogenetic analysis
• Genotypic resistance
• Viral tropism
• Anti-HTLV-I antibodies
• Anti-HTLV-II antibodies
• Anti-HBV antibodies
• HBV antigens (HbsAg, HbeAg)
• Anti-HCV antibodies
• HHV-8 antibodies and plasma viraemia
• HIV-1 proviral DNA copies (including 1- and 2-LTR, episomic and linear DNA)</td><td>phylogenetic analysis
• HIV-1 Genotypic resistance
• HIV Viral tropism
• HIV-1 proviral DNA copies
• Assessment of co-infections (e.g. Syphilis, HBV, HCV, HHV8 and others).

Female participants only: Cervical swabs and Pap smears will be taken on 3 occasions during the course of the study in order to test for Human Papilloma Virus (HPV) infection.

Should any of the above co-infections be detected during the course of the study, participants will be referred to their healthcare provider for clinical management.

In addition, the assessment of antiretroviral drug concentration may be performed.</td></tr>
<tr><td rowspan="2">6.6.5 Sample Collection, Storage and Shipment</td><td colspan="2">Change/Rationale:
Clarification of the split of immunonology and virology testing between NHLS and the Italian Core Laboratory, and storage conditions of samples awaiting shipment.
Inclusion of cervical swab and Pap smear sample collection from female participants and the relevant analytical laboratory.</td></tr>
<tr><td>Specialised immunological and virological evaluations (as detailed in sections 6.6.3 and 6.6.4) will be performed at the designated Core Lab. A Core Laboratory of Immunology and Virology (Core Laboratory of Immunology and Virology Ospedale S. Gallicano IFO, Rome) will centralize all the immunologic and virological assessment that go beyond the routine clinical monitoring of the patients.

Blood samples to be transferred to the Core Laboratory for immunologic and virologic investigations will be collected and processed according to the procedures described by the</td><td>Specialised immunological and virological evaluations (as detailed in sections 6.6.3 and 6.6.4) will be performed in part at the NHLS Laboratory and at the designated Core Lab. A Core Laboratory of Immunology and Virology (Core Laboratory of Immunology and Virology Ospedale S. Gallicano IFO, Rome) will perform all the immunologic testing to assess the anti-Tat immune response and the virological assessments that go beyond the routine clinical monitoring of the patients.

Blood samples to be transferred to the NHLS and/or to the Core Laboratory for immunologic and</td></tr>
</table>

	protocol-specific Specimen Management Manual which will be provided to the investigational site prior to study initiation. Thereafter, they will be stored at -80° C to be shipped to the Core Lab of immunology and virology (IFO San Gallicano Hospital, Rome). Shipment to the Core Lab will be done on dry ice, through a specialized courier, within 15 days of the date of sample collection.	virologic investigations will be collected and processed according to the procedures described by the protocol-specific Specimen Management Manual which will be provided to the investigational site prior to study initiation. Thereafter, cryopreserved cellular specimens and sera will be stored in liquid nitrogen or at -80 °C prior to be shipped to the Core Lab of immunology and virology (IFO San Gallicano Hospital, Rome). Shipment to the Core Lab will be done on dry ice, through a specialized courier, within 15 days of the date of sample collection.<br><br>As an exploratory test to detect possible HPV infection, cervical swabs and Pap smears will be collected from female participants at 3 time points throughout the study. Samples will be analysed at the S. Orsola Malpighi Hospital (Bologna, Italy)
**6.7 Schedule of Study Procedures**	**Change/Rationale:**<br>As cellular immune response will no longer be evaluated as a secondary endpoint, the following tests have been deleted from the relevant visit-specific procedures on **Days 0, 28, 56, 84, 140 & 336**:<br>• Lymphoproliferative response to Tat<br>• γIFN, IL-4 and IL-2 production in response to Tat	
	Addition of the following procedure to **Days 0, 140 & 336**:<br>• Cervical swab and Pap smear for detection of HPV infection (Female participants only)	
**8.2 Secondary Endpoint Variables and Measurements**	**Change/Rationale:**<br>Amended to correct an error with respect to cellular immune response being included as a secondary endpoint; the statistical analysis of safety data will be the only secondary endpoint.	
	In order to evaluate the safety of the Tat protein vaccination schedule in the study population, the following secondary endpoints will be considered and compared	In order to evaluate the safety of the Tat protein vaccination schedule in the study population, the following secondary endpoints will be considered and compared

	between active and placebo groups:<br><br>• Induction or increase of anti-Tat cellular-mediated immune response, in terms of lymphoproliferative response to Tat (CFSE staining), in vitro γIFN, IL-4, IL-2 production (Elispot) by peripheral blood mononuclear cells (PBMC) in response to Tat.<br>• Changes in physical examination findings from baseline;<br>• Changes in vital signs from baseline;<br>• Adverse events (including local and systemic reactions to the vaccination schedule occurring during the course of the study);<br>• Changes in standard laboratory safety parameters from baseline<br><br>The induction of the anti-Tat specific **cellular immune response** will be evaluated in the active as compared to the placebo group as follows:<br>1. Percentage of subjects with a positive cellular immune response to the vaccination schedule (responders). Responders will be defined as those vaccinated subjects that develop immune responses, at one or more time points after vaccination, above the following thresholds:<br>• γIFN production ≥ 3 fold and 30 spots/10^6 cells<br>• IL-2 production ≥ 3 fold<br>• IL-4 production ≥ 3 fold<br>• Lymphoproliferation fold increase ≥ 2<br>2. Increase of anti-Tat cellular-mediated immune response, evaluated in terms of fold/spots/proliferation index.	between active and placebo groups:<br><br>• Changes in physical examination findings from baseline;<br>• Changes in vital signs from baseline;<br>• Adverse events (including local and systemic reactions to the vaccination schedule occurring during the course of the study);<br>• Changes in standard laboratory safety parameters from baseline

<table>
<tr><td rowspan="2">9
Safety

9.1.1 Principal Investigator</td><td colspan="2">Change/Rationale:
Amended to clarify the relationship between the PI, Sponsor and DSMB, to indicate that the evaluation of adverse events should take into consideration the fact that subjects will be taking antiretroviral drugs and may default, and to clarify the procedures for un-blinding.</td></tr>
<tr><td>The principal investigator has a personal responsibility to closely monitor trial subjects and an inherent authority to take whatever measures necessary to insure their safety.
The principal investigator has the authority to terminate, suspend or request changes to a clinical trial for safety concerns and may delay an individual’s study vaccine administration if the investigator has some suspicion that the study vaccine might place a subject at significant risk.
The principal investigator or qualified designee determines severity and causality with respect to the investigational vaccine for each adverse event. The principal investigator will be blinded for this study.</td><td>The Principal Investigator (PI) has a personal responsibility to closely monitor trial subjects and to ensure their safety.
The principal investigator has the authority to request that the Sponsor convene the DSMB in the event of any safety concerns, and may delay an individual’s study vaccine administration if the investigator has some suspicion that the study vaccine might place a subject at significant risk. The principal investigator or qualified designee determines severity and causality with respect to the investigational vaccine for each adverse event. As the study will be conducted on patients taking antiretroviral therapy, the management of subject safety should take into consideration the known adverse reactions caused by the drugs used in antiretroviral combination therapy, and should also explore the possibility of any “antiretroviral drug exchange” practiced by the subject.
The principal investigator will be blinded for this study. In the event of an emergency where knowledge of the treatment allocation is required for proper clinical management of the subject, the principal investigator may require the un-blinding of the treatment assignment (active treatment or placebo). If possible, sponsor approval should be sought prior to any emergency un-blinding procedure (See Section 6.5).</td></tr>
</table>

**9.1.1.1 Study Vaccine Pausing Rules for the Principal Investigator**	**Change/Rationale:** Amended to remove the Principal Investigator's pause rules and to confirm that all decisions relating to safety will be referred to the DSMB.	
	If the principal investigator determines that a SUSAR OR a serious adverse event OR a Toxicity Grade 4 event OR an adverse event pattern of concern that is judged to be POSSIBLY, PROBABLY or CERTAINLY related to study vaccine has occurred, the principal investigator will pause administration of study vaccine in the trial. If the principal investigator pauses study vaccine administration he or she will record this in a memorandum to the study file and notify the sponsor and Local Medical Monitor.<br><br>If the principal investigator pauses study vaccine administration in a trial under the rules in this section and additional clinical information becomes available that reduces the principal investigator's assessment of causality, severity or toxicity grade such that the adverse event's causality, severity or toxicity grade no longer requires pausing then the principal investigator, with the agreement of the local medical monitor, may resume study vaccine administration with a memorandum to the study file and notification of the sponsor.	In the event of a safety concern, the principal investigator has the authority to request that the Sponsor convene an ad hoc review of the safety data by the DSMB. Based on this review, the DSMB may choose to pause further vaccine administration in the study. (See Section 10).
**9.1.2 Study Sponsor**	**Change/Rationale:** Amended to confirm that all decisions relating to safety will be referred to the DSMB.	
	The Sponsor has an institutional responsibility to ensure subject safety. This responsibility is vested in a Local Medical Monitor and a Data Safety Monitoring Board.	The Sponsor has an institutional responsibility to ensure subject safety. This responsibility is vested in a Data Safety Monitoring Board.

**9.1.2.1**<br>**Local Medical Monitor (LMM)**	**Change/Rationale:**<br>Amended to clarify the role of the Local Medical Monitor.	
	The LMM is the sponsor's representative and is a registered medical practitioner in his/her country of residence with experience in HIV/AIDS related research.<br>The local medical monitor will regularly review the safety of the product and may make a sponsor's assessment of severity and causality for adverse events that may upgrade the degree of severity and causality determined by the principal investigator.<br>The local medical monitor, like the principal investigator, will be blinded.	The LMM is the sponsor's representative and is a registered medical practitioner in his/her country of residence with experience in HIV/AIDS related research and HAART treatment.<br>The LMM will regularly review all safety data while being cognizant of the known adverse reactions caused by drugs used in antiretroviral combination therapy.<br>The LMM may assist the Principal Investigator in the assessment of severity and causality for adverse events, and help the PI in exploring the possibility of any "antiretroviral drug exchange" that may have been practiced by the subject. In addition to the ongoing review of adverse events, the LMM, by virtue of his/her location will be immediately available to discuss the significance and possible impact of a SUSAR/SAE with the principal investigator.<br>The LMM, like the principal investigator, will be blinded.
**9.1.2.1.1**<br>**Study Vaccine Pausing Rules for the Local Medical Monitor**	**Change/Rationale:**<br>Amended to remove the Local Medical Monitor's pause rules and to confirm that all decisions relating to safety will be referred to the DSMB.	
	If the local medical monitor determines that a SUSAR OR a serious adverse event OR a Toxicity Grade 4 event OR an adverse event pattern of concern that is judged to be POSSIBLY, PROBABLY or CERTAINLY related to study vaccine has occurred, the local medical monitor will pause administration of study vaccine in the trial. If the local	In the event of a safety concern the Local Medical Monitor in collaboration with the Principal Investigator will request that the Sponsor convene an ad hoc review of the relevant safety data by the DSMB. Based on this review, the DSMB may choose to pause further vaccine administration in the study (See Section 10).

	medical monitor independently pauses the administration of study vaccine in the study, he or she will record this in a memorandum to the study file and notify the principal investigator and sponsor who will then convene the DSMB. In all cases administration of study vaccine may resume only if permitted by the DSMB. Any changes to the protocol required by the DSMB as a condition of study vaccine resumption must be approved by, or submitted to, the Institutional Review Board/Ethics Committee and the national regulatory authority.	
**9.1.2.2 Data Safety Monitoring Board (DSMB)**<br><br>**(Section re-numbered 9.1.3)**	**Change/Rationale:**<br>Amended to clarify the role and responsibilities of the DSMB	
	The Sponsor will provide for the constitution of the Data Safety Monitoring Board for the monitoring of subjects' safety.<br>The DSMB is composed of medical specialists with experience in HIV/AIDS-related clinical research and evaluation of investigational product safety issues. The voting members cannot be directly involved with the conduct of the study. Additional subject area experts may be present to provide expertise if requested by the DSMB. The DSMB may review an individual SAE or it may choose to review adverse events, serious adverse events, solicited adverse events, and laboratory and vital signs data. The DSMB may un-blind any amount of safety information needed to conduct their assessment. All procedures associated with this review, including objectives, data handling, and elements to be included for review will be documented in DSMB minutes.<br><br>Based on its review and the	The Sponsor will provide for the constitution of the Data Safety Monitoring Board for the monitoring of subjects' safety.<br>The DSMB is composed of medical specialists with experience in HIV/AIDS-related clinical research and evaluation of investigational product safety issues. The primary responsibility of the DSMB is to protect the safety of subjects enrolled in the trial, by regular review of all safety data to verify no adverse changes to the pre-study risk-benefit ratio. The members cannot be directly involved with the conduct of the study. Additional subject area experts may be present to provide expertise if requested by the DSMB.<br>According to their requirements the DSMB may review an individual SAE, or it may choose to review adverse events, serious adverse events, solicited adverse events, and laboratory and vital signs data. The DSMB may un-blind any amount of safety information needed to conduct

	protocol stopping/pause rules the DSMB will make recommendations in the DSMB minutes to ISS regarding further conduct of the study and further administration of study treatment. The conclusions of the DSMB will be communicated to the investigator, Local Medical Monitor, Ethics Committees and the national regulatory authority for their concurrence. The sponsor agrees to abide by the decision of its DSMB with concurrence of the national regulatory authority, the Ethics Committee, the principal investigator and local Medical Monitor.	their assessment. All procedures associated with this review, including objectives, data handling, and elements to be included for review will be documented in the DSMB minutes.<br><br>Based on its review of the safety data, the DSMB will make recommendations in the DSMB minutes to ISS regarding further conduct of the study and further administration of study treatment. The conclusions of the DSMB will be communicated to the Sponsor, Principal Investigator, Local Medical Monitor, Ethics Committees and the national regulatory authority for their concurrence. The sponsor agrees to abide by the decision of its DSMB with concurrence of the national regulatory authority and the Ethics Committee.
**9.2.2 Serious Adverse Event (SAE)**	**Change/Rationale:**<br>Amended to clarify that the Principal Investigator or qualified designee will determine the 'serious' classification of an adverse event.	
	Seriousness refers to the outcome of an adverse event. Seriousness is determined by both the principal investigator and local medical monitor. If either the principal investigator or local medical monitor determines an event to be serious, it will be classified as such.	Seriousness refers to the outcome of an adverse event. Seriousness is determined by the principal investigator (or qualified designee).
**9.2.3 Adverse Drug Reaction (ADR)**	**Change/Rationale:**<br>Amended to clarify that the Principal Investigator or qualified designee will determine the 'relatedness' classification of an adverse event to the investigational product.	
	An ADR is any untoward and unintended response to any dose of an investigational medicinal product administered in a clinical trial when the Investigator, Sponsor or Local Medical Monitor has judged that there is at least a reasonable possibility that the event	An ADR is any untoward and unintended response to any dose of an investigational medicinal product administered in a clinical trial when the Principal Investigator (or qualified designee) has judged that there is at least a reasonable possibility

	was related to the product.	that the event was related to the product.
**9.3.2 Assessing a Causal Relationship (Relatedness)**	**Change/Rationale:**<br>Amended to clarify that the investigator may be assisted by the local medical monitor in determining the 'relatedness' classification of an adverse event to the investigational product.	
	For all adverse events, the investigator and the local medical monitor may determine a **causal relationship,** to the study vaccine without the knowledge of whether the Tat protein vaccine or placebo was administered.<br>.........<br>The principal investigator and the local medical monitor may both determine causality. It is expected that in the event of any uncertainty, communication and consultation may occur in the assessment of the causality of adverse events. The greatest degree of causal relationship (certain > probable > possible > unlikely related > unrelated) determined by either the investigator or local medical monitor after their discussions will determine the ultimate classification of the adverse event. Certain, probable and possible are considered to be related. Unrelated and unlikely related are considered to be unrelated.	For all adverse events, the investigator determines a **causal relationship** to the study vaccine without the knowledge of whether the Tat protein vaccine or placebo was administered.<br>………<br>The investigator may be assisted by the local medical monitor in determining causality. It is expected that in the event of any uncertainty, communication and consultation may occur in the assessment of the causality of adverse events. Certain, probable and possible are considered to be related. Unrelated and unlikely related are considered to be unrelated.
**9.4 Adverse Event Treatment, Follow-up and Outcome**	**Change/Rationale:**<br>Amended to add a paragraph clarifying the procedures for un-blinding.	
		Since the principal investigator will be blinded for this study, in the event of an emergency where knowledge of the treatment allocation is required for proper clinical management of the subject, the principal investigator may require the un-blinding of the treatment assignment (active treatment or placebo). If possible, sponsor approval should be sought prior to any emergency un-blinding procedure.

<table>
<tr><td rowspan="2">9.5
Reporting of Serious Adverse Events/Serious Adverse Drug Reactions</td><td colspan="2">Change/Rationale:
Amended to clarify the resposnibility for assessment of SAEs, to include Triclinium into the telephonic reporting of a SUSAR and to add a paragraph clarifying the procedures for un-blinding.</td></tr>
<tr><td>Serious adverse events, which include SUSARs, are to be reported to the sponsor for the entire study period. SUSARs are reported even after the trial is over, if the sponsor, local medical monitor or principal investigator becoming aware of them.
The site will be provided with specific reporting procedures including the Adverse Event CRF and any supplemental reporting forms to be used. Serious adverse events will be reported on the Adverse Event CRF using a recognized medical term or diagnosis that accurately reflects the event.

Serious adverse events will be assessed by the investigator and the local medical monitor according to their roles (as described in Sections 9.1.1 and 9.1.2.1) for severity, causal relationship to the study vaccine, and expectedness. The onset and resolution dates of the event and the action taken in response to the event will be documented. If the event has not resolved by the final study visit, it will be documented as “ongoing” on the CRF, however, follow-up of the SAE must continue until resolved. Information recorded on the CRF must be substantiated in the source documents.

The AE CRF containing a serious adverse event and the SAE Report completed for that event must be faxed by the principal investigator or his/her designee within 24</td><td>Serious adverse events, which include SUSARs, are to be reported to the sponsor for the entire study period. SUSARs are reported even after the trial is over, if the sponsor, local medical monitor or principal investigator becomes aware of them.
The site will be provided with specific reporting procedures including the Adverse Event CRF and any supplemental reporting forms to be used. Serious adverse events will be reported on the Adverse Event CRF using a recognized medical term or diagnosis that accurately reflects the event.

Serious adverse events will be assessed by the investigator as described in Section 9.1.1 for severity, causal relationship to the study vaccine, and expectedness. The onset and resolution dates of the event and the action taken in response to the event will be documented. If the event has not resolved by the final study visit, it will be documented as “ongoing” on the CRF, however, follow-up of the SAE must continue until resolved. Information recorded on the CRF must be substantiated in the source documents.

The AE CRF containing a serious adverse event and the SAE Report completed for that event must be faxed by the principal investigator or his/her designee within 24 hours (one calendar day) of the</td></tr>
</table>

	**hours** (one calendar day) of the investigative site becoming aware of the event to the local medical monitor and to Triclinium. The AE CRF should be completed with all information known at the time and a hard copy printed for faxing; the SAE Report (paper form) should be completed and both forms faxed (even if all information concerning the event is not yet known) within the first 24 hours of awareness of the event.<br><br>All serious adverse events that the investigator suspects are related to the study vaccine (SUSARs) should be **telephoned to the local medical monitor immediately upon the investigator's awareness of the event.** Following assessment of the information, if the local medical monitor is required by the protocol or chooses to suspend enrolment she/he shall immediately create a written memorandum for record to the study file and telephonically notify the Principal Investigator, Sponsor and Triclinium of this act.<br><br>Contact information for all safety personnel are contained in the Team Contact List which will be stored on site in the investigator Site File and maintained by Triclinium.<br><br>Investigators **must not wait** to collect additional information to fully document the event before notifying the local medical monitor and Triclinium of a serious adverse event. The initial notification should include the following (at minimum):<br><br>• Protocol number and name and contact number of the investigator<br>• Subject study number (and	investigative site becoming aware of the event to the local medical monitor and to Triclinium. The AE CRF should be completed with all information known at the time and a hard copy printed for faxing; the SAE Report (paper form) should be completed and both forms faxed (even if all information concerning the event is not yet known) within the first 24 hours of awareness of the event.<br><br>All serious adverse events that the investigator suspects are related to the study vaccine (SUSARs) should be **telephoned to the local medical monitor and to Triclinium immediately upon the investigator's awareness of the event.** Since the principal investigator will be blinded for this study, in the event of an emergency where knowledge of the treatment allocation is required for proper clinical management of the subject, the PI may require the un-blinding of the treatment assignment (active treatment or placebo). If possible, sponsor approval should be sought prior to any emergency un-blinding procedure.<br><br>Contact information for all safety personnel are contained in the Team Contact List which will be stored on site in the investigator Site File and maintained by Triclinium.<br><br>Investigators **must not wait** to collect additional information to fully document the event before notifying the local medical monitor and Triclinium of a serious adverse event. The initial notification should include the following (at minimum):

	initials and date of birth, if available)<br>• Date/s subject received study vaccine<br>• Serious adverse event(s) and date of event onset<br>• Current status of subject<br><br>ISS has authorized Triclinium to execute its responsibilities for safety report submission to the appropriate regulatory authorities within specific time periods of being notified of the event; the investigative site is responsible for notification of all SAE reports to the relevant Ethics Committee within the appropriate reporting time periods. ISS will notify the DSMB of all SAEs within three working days of becoming aware of an event and will provide all follow-up information in a timely manner.	• Protocol number and name and contact number of the investigator<br>• Subject study number (and initials and date of birth, if available)<br>• Date/s subject received study vaccine<br>• Serious adverse event(s) and date of event onset<br>• Current status of subject<br><br>ISS has authorized Triclinium to execute its responsibilities for safety report submission to the appropriate regulatory authorities within specific time periods of being notified of the event; the investigative site is responsible for notification of all SAE reports to the relevant Ethics Committee within the appropriate reporting time periods. Triclinium will notify the DSMB of all SAEs within three working days of becoming aware of an event and will provide all follow-up information in a timely manner.
**9.7<br>Follow-up of Subjects Who Become Pregnant**	**Change/Rationale**:<br>Amended to remove the LMM from the reporting lines in the event of the investigator only becoming aware of a participant pregnancy post completion of the study.	
	If it is determined after completion of the study that a subject became pregnant during the study, the subject should notify the investigator. The pregnancy must be reported to the local medical monitor and the status of the mother and child after delivery will be obtained and reported, when possible.	If it is determined after completion of the study that a subject became pregnant during the study, the subject should notify the investigator. The pregnancy must be reported to Triclinium and the status of the mother and child after delivery will be obtained and reported, when possible.

<table>
<tr>
<td rowspan="2">10
STUDY PAUSE /STOPPING RULES
10.1 General rules for a study pause</td>
<td colspan="2">Change/Rationale:
Amended to remove the predefined pause/stopping rules for the DSMB, allowing them to review any event that represents a safety concern and to take whatever appropriate action is required.</td>
</tr>
<tr>
<td>These rules govern the stopping of study vaccine administration at any time during the study. The rules for pausing study vaccine administration by the principal investigator and local medical monitor are in Section 9.1.1.1 and 9.1.2.1.1).

The rules for stopping further enrolment and study vaccine administration by the DSMB are below:
• Death in any subject unless the DSMB determines it is UNRELATED to the Tat protein vaccine
• An anaphylactic reaction to the Tat protein vaccine in any subject
• A life-threatening adverse event in any subject unless the DSMB determines it is UNRELATED to the Tat protein vaccine.
• The occurrence of a severe (Toxicity Grade 4) or serious adverse event unless the DSMB determines it is UNRELATED to the Tat protein vaccine.
• A pattern of significant symptoms, physical findings or laboratory abnormalities (adverse events) that, although individually minor, collectively represent a safety concern in the opinion of the investigator or the medical monitor and are judged by the DSMB to be CERTAINLY, PROBABLY or POSSIBLY related to the Tat protein vaccine.

Please note that PREGNANCY in any subject will automatically</td>
<td>The sponsor will appoint an independent DSMB to monitor the safety of the subjects enrolled in the study. The DSMB will review all serious adverse events reports in real-time and will receive periodic reports of all non-serious adverse events; the DSMB may un-blind any amount of safety information needed to conduct their assessments. The DSMB will determine whether the study should be continued, modified or stopped for safety concerns. Guidelines for making such a decision will be developed by the sponsor in consultation with the DSMB</td>
</tr>
</table>

	discontinue an individual subject from any further administration of study vaccine.<br><br>The DSMB may permit resumption of study vaccine administration if the study pause was for reasons less severe than those in the DSMB stopping rules. The DSMB may permit resumption of enrolment if it judges that changes to the study protocol will eliminate or greatly reduce the safety risks specified in the stopping rules. In the absence of study protocol changes the DSMB must follow the DSMB study stopping rules.<br><br>If a decision to resume study enrolment and study vaccine administration is made the DSMB will record their judgment in a memorandum to the study file and notify the sponsor, who will then forward the DSMB memorandum to the medical monitors and principal investigators. The clinical site will be allowed to resume activities upon receipt of written notification from the sponsor. The appropriate regulatory authority will be informed in writing if the study is stopped and will be informed in writing of the decision by the DSMB to resume or discontinue study activities.	

**12<br>Statistical Analysis<br>12.2 Endpoints**	**Change/Rationale:**<br>Amended to correct an error with respect to cellular immune response being included as a secondary endpoint; the statistical analysis of safety data will be the only secondary endpoint.	
	**Secondary Endpoint Variables**<br>• Lymphoproliferative response to Tat (CFSE staining)<br>• *in vitro* γIFN, IL-4, IL-2 production against Tat<br>• Physical examination data<br>• Vital signs data<br>• Adverse events<br>• Laboratory safety parameters<br>• Virology	**Secondary Endpoint Variables**<br>• Physical examination data<br>• Vital signs data<br>• Adverse events<br>• Laboratory safety parameters<br>• Virology
**12.7<br>Secondary Endpoint Analysis**	**Change/Rationale:**<br>Amended to correct an error with respect to cellular immune response being included as a secondary endpoint; the statistical analysis of safety data will be the only secondary endpoint.	
	To evaluate the **cellular immune response** to vaccination, "responders" will be defined as vaccinated subjects that develop an immune response at one or more time points after vaccination and above the following levels:<br>• γIFN production ≥3 fold and 30 spots/10^6 cells,<br>• IL-2 production ≥ 3 fold<br>• IL-4 production ≥3 fold<br>• Lymphoproliferation fold increase ≥ 2<br>The percentage of responders will be compared between active and placebo groups using the Chi-Square test. Increases of anti-Tat cellular-mediated immune response will also be evaluated in terms of fold/spots/proliferation index. Multivariate regression models will be used to test for any dependency of variables.<br><br>Change from baseline in physical	Changes from baseline in physical examination results, vital signs data and laboratory parameters will be summarized for both treatment groups.<br>MedDRA dictionary will be used for coding adverse events. Adverse events will be summarized by system organ class, preferred term, severity and relationship with the vaccination; the incidence of adverse events "possibly", "probably" or "certainly" related to vaccination will be computed. All serious adverse events (SAEs) will be summarized by treatment group, subject, gender, age, duration of the event, action taken, investigator's assessment of causality and outcome.<br><br>Change from baseline will be summarized for $CD4^+$ T cells and HIV viral load. Haematology,

	examination results, vital signs data and laboratory parameters will be summarized for both treatment groups.<br>MedDRA dictionary will be used for coding adverse events. Adverse events will be summarized by system organ class, preferred term, severity and relationship with the vaccination; the incidence of adverse events "possibly", "probably" or "certainly" related to vaccination will be computed. All serious adverse events (SAEs) will be summarized by treatment group, subject, gender, age, duration of the event, action taken, investigator's assessment of causality and outcome.<br><br>Change from baseline will be summarized for $CD4^+$ T cells and HIV viral load. Haematology, clinical chemistry and urinalysis results will be graded according to Appendix I and will be presented for both treatment groups.	clinical chemistry and urinalysis results will be graded according to Appendix I and will be presented for both treatment groups.
**16**<br>**Study Timetable**	**Change/Rationale:**<br>Amended to allow for the change in estimated study start date.	
	The enrolment of the first subject is planned to take place in January 2011 and will continue until the last treatment number has been allocated.	The enrolment of the first subject is planned to take place in October 2011 and will continue until the last treatment number has been allocated.

**19**<br>**References**	Addition of the following references:<br><br>1. **Cafaro A, Bellino S, Titti F, Maggiorella MT, Sernicola L, Wiseman WR, Venson D, Karl AJ, O'Connor D, Monini P, Robert-Guroff M, Ensoli B.** Impact of Viral Dose and Major Histocompatibility Complex Class IB Haplotype on Viral Outcome in Tat-vaccinated Mauritian Cynomolgus Monkeys upon Challenge with SHIV89.6P Journal of Virology.84:8953-8958, 2010.<br><br>2. **Ensoli B, Bellino S, Tripiciano A, Longo O, Francavilla V, Marcotullio S, Cafaro A, Picconi O, Paniccia G, Scoglio A, Arancio A, Ariola C, Ruiz Alvarez MJ, Campagna M, Scaramuzzi D, Iori C, Esposito R, Mussini C, Ghinelli F, Sighinolfi L, Palamara G, Latini A, Angarano G, Ladisa N, Soscia F, Mercurio VS, Lazzarin A, Tambussi G, Visintini R, Mazzotta F, Di Pietro M, Galli M, Rusconi S, Carosi G, Torti C, Di Perri G, Bonora S, Ensoli F, Garaci E.** Therapeutic Immunization with HIV-1 Tat Reduces Immune Activation and Loss of Regulatory T-Cells and Improves Immune Function in Subjects on HAART. PLoS ONE, 5(11):e13540 (2010).
**17.2**<br>**Amendments to the Protocol**	**Change/Rationale:**<br>New section added to summarize protocol changes from version 2.0 to version 3.0
**Appendix ll.**<br>**Schedule of Protocol Visits and Procedures**	**Change/Rationale:**<br>Amended to delete cellular immune response tests from the relevant visit-specific procedures and to add the collection of cervical swabs and pap smear for the detection of HPV infection in females at 4 time points.

Section and Change rationale	Version 3.0	Version 4.0
**Cover Page and document footer**<br>Updated to amend the protocol version	ISS T-003 Protocol Version 3.0, 29-July-2011	ISS T-003 Protocol Version 4.0, 04-Dec-2012
**Emergency contacts**<br>Updated to include current Project Manager	Ms Tracy Southwood<br>tracy.southwood@triclinium.net	Dr Havana Chikoto<br>Havana.chikoto@triclinium.net
**Section: protocol synopsis, 4** (study design), **5.2** (study duration) **, 6.7** (schedule of study procedures) **and Appendix II** (schedule of protocol visits and procedures)<br>Changed the screening window from -21 days to -35 days. The relatively long turn around time for the receipt, by the site, of anti-tat results from the IFO lab resulted in difficulties in completing screening procedures within 21 days.	-21 days	- 35 days
**Section: protocol synopsis, 6.2** (Exclusion criteria)<br>Clarified exclusion criterion number 4	Any current neoplastic disease	Any current neoplastic disease *[NOTE: Criterion is meant to exclude volunteers with active invasive cancer. Volunteers who present with intraepithelial neoplasia during the course of the study are to continue with all study procedures.]*
**Section: protocol synopsis**<br>Added text on interim analysis	There was no text on interim analysis	**Interim Analyses**<br>At least 50% of the original planned population (at least 100 subjects) will be included in an interim analysis. Data derived from the interim analysis will be used to re-calculate the sample size.

Section and Change rationale	Version 3.0	Version 4.0
**Section 6.6.3: Immunological evaluations**<br>Clarification of when the second line testing will take place	The following parameters will be determined:<br>Assessment of anti-Tat cellular immune response:<br>- Lymphoproliferative response to Tat (CFSE staining)<br>- *In vitro* γIFN, IL-4 and IL-2 production in response to Tat (Elispot).<br><br>Depending on the availability of residual specimens (PBMC, serum and plasma), the following tests will also be performed:	**Second line immunology testing**<br>The second line immunology testing will be performed retrospectively after the un-blinding, at the end of the study, on subgroups of participants defined on the basis of immunogenicity results.<br>In particular, lymphoproliferative responses (CFSE staining) or *in vitro* γIFN, IL-4 and IL-2 production in response to Tat (ELIspot) will be performed depending on the sample cell viability and recovery.<br><br>In addition, depending on the availability of residual specimens (PBMC, serum and plasma), the following tests will also be performed:
**Section 6.8: Schedule of study procedures**<br>Added text that outlines the process of giving results to subjects post Visit 12	There was no text on giving results post visit 12	**DAY 343: Dissemination of results (Day 343 ± 7 days)**<br>Within 7 days of the study termination visit, a site staff member will telephonically contact the subject to inform them of the results of the following tests done at the study termination visit:<br>• Standard haematology, clinical chemistry, coagulation and urinalysis assessments<br>• $CD4^+$ T cell counts<br>• HIV-1 plasma viraemia<br><br>Results of the Pap smear will be telephonically communicated to the subject as soon as they become available.<br><br>In both cases, if results are abnormal and require that the subject be referred to a health facility, the subject will be requested to visit the site for the collection of a referral letter.

Section and Change rationale	Version 3.0	Version 4.0
**Section 12.8 Interim analysis**<br>Added text on interim analysis	There was no text on interim analysis	**12.8 Interim analysis**<br><br>**12.8.1 Rationale/reason for interim analysis**<br>Sample size determination is based on hypothetical positive response rates of not less than 80% and not more than 60% in the treatment and placebo group, respectively (Paragraph 12.3). These assumptions stem from information related to vaccine trials and observational studies conducted both in Italy and South Africa. These studies are now completed and have provided additional information. In particular, data from the studies in Italy indicate about 18% of anti-Tat Ab positive subjects on ART vs 28% positive subjects in South Africa (ISS OBS T-004, ClinicalTrials.gov identifier: NCT01359800). In addition, a longitudinal observational study completed in Italy (ISS OBS T-002, ClinicalTrials.gov identifier: NCT01024556) indicate an anti-Tat seroconversion of 9%. Therefore, the cumulative rate of anti-Tat seroconversion in the placebo group of the present study is expected not to exceed 40%, which is lower than what was indicated in the clinical protocol (60%) It is therefore necessary to re-evaluate the sample size upon interim evaluation of the data.<br><br>The interim analysis will be conducted only if approved before the 200 participants foreseen by the protocol are enrolled into the study. All participants that have signed the informed consent form and have been vaccinated at least once by the time of the conclusion of the interim analysis will complete the study as per protocol, irrespective of sample size re-evaluation.<br><br>**12.8.2 Statistical analysis and population**<br>At least 50% of the original planned population will be included in the interim analysis (i.e. at least 100 subjects). At the time of the interim analysis, data for the humoral immune response to Tat will be used to re-calculate the sample size. "Responders" will be

Section and Change rationale	Version 3.0	Version 4.0
		defined using the same criteria as described in section 12.6. Data for all subjects who were randomised and who received at least 2 vaccinations will be included in this analysis. No other data will be analysed for the purpose of the interim analysis.<br><br>**12.8.3 Preserving of blindness**<br>An independent statistical vendor, who will not be involved in the final statistical analysis, will perform the sample size re-calculation.<br><br>**12.8.4 Timing of interim analysis**<br>The sample size re-calculation will be performed when at least 100 subjects have received at least 2 vaccinations, i.e., have completed at least the Week 8 assessments (Visit 6).<br><br>**12.8.5 Adjustments to Type 1 error**<br>Since no formal treatment group comparison will be performed on the humoral immune response data, it will not be necessary to adjust for any inflation of the overall Type 1 error rate.
**Appendix 2: Schedule of protocol visits and procedures**<br>Changed the screening period and added telephonic contact with subject post Visit 12	Screening period was up to Week -3 (Day -21)<br><br>There was no text on the telephonic contact	Screening period is now up to Week -5 (Day -35)<br><br>Added telephonic contact at Week 55 and Day 343. Also added the following footnote:<br><br>"Site to contact subject telephonically to give results of the following tests done during Visit 12: haematology, clinical chemistry, coagulation and urinalysis assessments CD4+ T cell counts, HIV-1 plasma viraemia. Pap smear results will be communicated as soon as they become available. Subject will be requested to come to the site if there is a need for a referral to a health facility."

18 INVESTIGATORS AGREEMENT

A Phase II, Randomized, Double-Blind, Placebo-Controlled Trial to evaluate the Immunogenicity and Safety of a Therapeutic, Recombinant, Biologically Active HIV-1 Tat Protein Vaccine in HIV-Infected, Anti-Tat Negative, ARV-Treated Adult Volunteers

Protocol Number: ISS T-003

Protocol Version: ISS T-003 Protocol Version 4.0 20-Feb-2013

Sponsor Representative

[signature] 4/MAR/2013

Barbara Ensoli, MD, PhD — Signature — Date
AIDS National Center
Istituto Superiore di Sanità
Viale Regina Elena, 299
00161 - Rome, Italy

Clinical Site - Principal Investigator

I have carefully read this protocol and agree to conduct the study in accordance with GCP, Declaration of Helsinki, local laws and regulations relevant to the use of new and approved therapeutic agents in human subjects.
I agree that ISS, its delegates and Regulatory Authorities have direct access to all study documentation.
I agree to obtain Written Informed Consent from all participating subjects or their legal representative.
I agree to maintain the confidentiality of all information received or developed in connection with this protocol.

[signature] 07 | Mar | 2013

Prof. Maphoshane Nchabeleng — Signature — Date
Medunsa Clinical Research Unit (MeCRU)
Medunsa Campus
University of Limpopo
Gauteng
South Africa

19 REFERENCES

1. **Arora VK, Fredericksen BL, Garcia JV.** Nef: agent of cell subversion. Microbes and Infections 2002, 4:189-199.

2. **Arya SK, Guo C, Josephs SF, Wong-Staal F**. Trans-activator gene of human T-lymphotropic virus type III (HTLV-III). Science 1985, 229:69-73.

3. **Bellino S, Francavilla V, Longo O, Tripiciano A, Paniccia G, Arancio A, Fiorelli V, Scoglio A, Collacchi B, Campagna M, Lazzarin A, Tambussi G, Tassan Din C, Visintini R, Narciso P, Antinori A, D'Offizi G, Giulianelli M, Carta M, Di Carlo A, Palamara G, Giuliani M, Laguardia ME, Monini P, Magnani M, Ensoli F, Ensoli B.** Parallel Conduction of the Phase I Preventive and Therapeutic Trials Based on the Tat Vaccine Candidate. Rev Rec Clin Trials, 2009, 4, 195-204.

4. **Battegay M, Nuesch R, Hirschel B, Kaufmann GR**, Immunological recovery and antiretroviral therapy in HIV-1 infection. Lancet Infect. Dis. 6, 280-287 (2006).

5. **Burton DR.** A vaccine for HIV type 1: the antibody perspective. Proc Natl Acad Sci USA 1997, 94:10018-10023.2004, 2:357-376.

6. **Buttò S, Fiorelli V, Tripiciano A, Ruiz-Alvarez MJ, Scoglio A, Ensoli F, Ciccozzi M, Collacchi B, Sabbatucci M, Cafaro A, Guzmán CA, Borsetti A, Aiuti F, Vardas E, Colvin M, Lukwyia M, Rezza G, Ensoli B, the Tat Multicentric Study Group.** Recognition of a clade B-derived HIV-1 Tat protein by antibodies of infected individuals from Uganda, South Africa and Italy is associated with conservation of Tat epitopes in the infecting viruses Cross-Recognition of the Clade B HIV-1 Tat Protein Vaccine Candidate by Antibodies from HIV-1-Infected Italian, Ugandan and South African Individuals. J Infect Dis 2003, 188:1171-1180.

7. **Byrnes AA, Harris DM, Atabani SF, Sabundayo BP, Langan SJ, Margolick JB, Karp CL**, Immune activation and IL-12 production during acute/early HIV infection in the absence and presence of highly active, antiretroviral therapy. J. Leukoc. Biol. 84, 1447-1453 (2008).

8. **Cafaro A, Caputo A, Fracasso C, Maggiorella MT, Goletti D, Baroncelli S, Pace M, Sernicola L, Koanga-Mogtomo ML, Betti M, Borsetti A, Belli R, Akerblom L, Corrias F, Buttò S, Heeney J, Verani P, Titti F, Ensoli B.** Control of SHIV-89.6P-infection of cynomolgus monkeys by HIV-1 Tat protein vaccine. Nat Med 1999, 5:643-650.

9. **Cafaro A, Caputo A, Maggiorella MT, Baroncelli S, Fracasso C, Pace M, Borsetti A, Sernicola L, Negri DR, Ten Haaft P, Betti M, Michelini Z, Macchia I, Fanales-Belasio E, Belli R, Corrias F, Buttò S, Verani P, Titti F, Ensoli B.** SHIV89.6P pathogenicity in cynomolgus monkeys and control of viral replication and disease onset by human immunodeficiency virus type 1 Tat vaccine. J Med Primatol 2000, 29:193-208.

10. **Cafaro A, Titti F, Fracasso C, Maggiorella MT, Baroncelli S, Caputo A, Goletti D, Borsetti A, Pace M, Fanales-Belasio E, Ridolfi B, Negri DR, Sernicola L, Belli R, Corrias F, Macchia I, Leone P, Michelini Z, Ten Haaft P, Buttò S, Verani P, Ensoli B.** Vaccination with DNA containing tat coding sequences and unmethylated CpG motifs protects cynomolgus monkeys upon infection with simian/human immunodeficiency virus (SHIV89.6P). Vaccine 2001, 19:2862-2877.

11. **Cafaro A, Bellino S, Titti F, Maggiorella MT, Sernicola L, Wiseman WR, Venson D, Karl AJ, O'Connor D, Monini P, Robert-Guroff M, Ensoli B.** Impact of Viral Dose and Major Histocompatibility Complex Class IB Haplotype on Viral Outcome in Tat-vaccinated Mauritian Cynomolgus Monkeys upon Challenge with SHIV89.6P Journal of Virology.84:8953-8958, 2010.

12. **Caputo A, Gavioli R, Ensoli B.** Recent advances in the development of HIV-1 Tat-based vaccines. Curr HIV Res, 2004(2)347-376

13. **Chang HC, Samaniego F, Nair BC, Buonaguro L, Ensoli B.** HIV-1 Tat protein exits from cells via a leaderless secretory pathway and binds to extracellular matrix-associated heparan sulfate proteoglycans through its basic region. AIDS 1997, 11:1421-1431.

14. **Chehimi J, Azzoni L, Farabaugh M,Creer SA, Tomescu C, Hancock A, Mackiewicz A, D'Alessandro L, Ghanekar S, Foulkes AS, Mounzer K,Kostman J, Montaner LJ**, Baseline viral load and immune activation determine the extent of reconstitution of innate immune effectors in HIV-1-infected subjects undergoing antiretroviral treatment. J. Immunol. 179, 2642-2650 (2007).

15. **Demirhan I, Chandra A, Sarin PS, Hasselmayer O, Hofmann D, Chandra P**. Inhibition of tat-mediated HIV-1-LTR transactivation and virus replication by sulfhydryl compounds with chelating properties. Anticancer Res 2000, 20:2513-2517.

16. **Ensoli B, Barillari G, Salahuddin SZ, Gallo RC, Wong-Staal F**. Tat protein of HIV-1 stimulates growth of cells derived from Kaposi's sarcoma lesions of AIDS patients. Nature 1990, 345:84-86.

17. **Ensoli B, Buonaguro L, Barillari G, Fiorelli V, Gendelman R, Morgan RA, Wingfield P, Gallo RC**. Release, uptake, and effects of extracellular human immunodeficiency virus type1 Tat protein on cell growth and viral transactivation. J Virol 1993, 67:277-287.

18. **Ensoli B, Gendelman R, Markham P, Fiorelli V, Colombini S, Raffeld M, Cafaro A, Chang HK, Brady JN, Gallo RC**. Synergy between basic fibroblast growth factor and HIV-1 Tat protein in induction of Kaposi's sarcoma. Nature 1994, 371:674-680.

19. **Ensoli B, Fiorelli V, Ensoli F, Cafaro A, Titti F, Buttò S, Monini P, Magnani M, Caputo A, Garaci E.** Candidate HIV-1 Tat vaccine development: from basic science to clinical trials. AIDS 2006, 20:2245-2261.

20. **Ensoli B, Fiorelli V, Ensoli F, Lazzarin A, Visintini R, Narciso P, Di Carlo A, Monini P, Magnani M, Garaci E.** The therapeutic phase I trial of the recombinant native HIV-1 Tat protein. AIDS 2008, 22:2207–2217

21. **Ensoli B, Fiorelli V, Ensoli F, Lazzarin A, Visintini R, Narciso P, Di Carlo A, Tripiciano, Longo O, Bellino S, Francavilla V, Paniccia G, Arancio A, Scoglio A, Collacchi B, Ruiz Alvarez MJ, Tambussi G, Tassan Din C, Palamara G, Latini A, Antinori A, D'Offizi G, Giuliani M, Giulianelli M, Carta M, Monini P, Magnani M,Garaci E**. The preventive phase I trial with the HIV-1 Tat-based vaccine;Vaccine 28**,** 371-378 (2009).

22. **Ensoli B, Bellino S, Tripiciano A, Longo O, Francavilla V, Marcotullio S, Cafaro A, Picconi O, Paniccia G, Scoglio A, Arancio A, Ariola C, Ruiz Alvarez MJ, Campagna M, Scaramuzzi D, Iori C, Esposito R, Mussini C, Ghinelli F, Sighinolfi L, Palamara G, Latini A, Angarano G, Ladisa N, Soscia F, Mercurio VS, Lazzarin A, Tambussi G, Visintini R, Mazzotta F, Di Pietro M, Galli M, Rusconi S, Carosi G, Torti C, Di Perri G, Bonora S, Ensoli F, Garaci E.** Therapeutic Immunization with HIV-1 Tat Reduces Immune Activation and Loss of Regulatory T-Cells and Improves Immune Function in Subjects on HAART. PLoS ONE, 5(11):e13540 (2010).

23. **Ferrantelli F, Cafaro A, Ensoli B**. Nonstructural HIV proteins as targets for prophylactic or therapeutic vaccines. Curr Opin Biotechnol. 2004 Dec, 15(6):543-556.

24. **Fisher AG, Feinberg MB, Josephs SF, Harper ME, Marselle LM, Reyes G, Gonda MA, Aldovini A, Debouk C, Gallo RC, et al.** The trans-activator gene of HTLV-III is essential for virus replication. Nature 1986, 320:367-371.

25. **Glencross DK, Janossy G, Coetzee LM, Lawrie D,Scott LE, Sanne I, McIntyre JA, Stevens W**, CD8/CD38 activation yields important clinical information of effective antiretroviral therapy: findings from the first year of the CIPRA-SA cohort. Cytometry B Clin. Cytom. 74 Suppl 1, S131-S140 (2008).
26. **Hazenberg MD, Otto SA, van Benthem BH, Roos MT, Coutinho RA, Lange JM, Hamann D, Prins M, Miedema F**, Persistent immune activation in HIV-1 infection is associated with progression to AIDS. AIDS 17, 1881-1888 (2003).

27. **Hunt PW, Martin JN, Sinclair E, Bredt B, Hagos E, Lampiris H, Deeks SG**, T cell activation is associated with lower CD4+ T cell gains in human immunodeficiency virus infected patients with sustained viral suppression during antiretroviral therapy. J. Infect. Dis. 187, 1534-1543 (2003).

28. **Hunt PW**, Role of immune activation in HIV pathogenesis. Curr. HIV. /AIDS Rep. 4, 42-47 (2007).

29. **James CO, Huang MB, Khan M, Garcia-Barrio M, Powell MD, Bond VC.** Extracellular Nef protein targets $CD4^+$ T cells for apoptosis by interacting with CXCR4 surface receptors. J Virol 2004 Mar, 78(6):3099-3109.

30. **Kelley CF, Kitchen CM, Hunt PW, Rodriguez B, Hecht FM, Kitahata M, Crane HM, Willig J, Mugavero M, Saag M, Martin JN, Deeks SG**, Incomplete peripheral

CD4+ cell count restoration in HIV-infected patients receiving long-term antiretroviral treatment. Clin. Infect. Dis. 48, 787-794 (2009).

31. **Koedel U, Kohleisen B, Sporer B et al.** HIV Type 1 Nef protein is a viral Factor for Leukocyte Recruitment into the Central Nervous System. Journal of Immunology 1999, 163:1237-1245.

32. **Krone WJ, Debouck C, Epstein LG, Heutink P, Meloen R, Goudsmit J**. Natural antibodies to HIV-tat epitopes and expression of HIV-1 genes in vivo. J Med Virol 1988, 26:261-270.

33. **Longo O, Tripiciano A, Fiorelli V, Bellino S, Scoglio A, Collacchi B, Ruiz Alvarez MJ, Francavilla V, Arancio A, Paniccia G, Lazzarin A, Tambussi G, Tassan Din C, Visintini R, Narciso P, Antinori A, D'Offizi G, Giulianelli M, Carta M, Di Carlo A, Palamara G, Giuliani M, Laguardia ME, Monini P, Magnani M, Ensoli F, Ensoli B.** Phase I therapeutic trial of the HIV-1 Tat protein and long term follow-up. Vaccine 27 (2009) 3306–3312

34. **Merck/HVTN News release.** Vaccination and Enrolment Are Discontinued in Phase II Trials of Merck's Investigational HIV Vaccine Candidate-Interim Analysis of STEP Study Shows Vaccine was not Effective, 2007.

35. **Ratner L, Haseltine W, Patarca R, Livak KJ, Starcich B, Josephs SF, Doran ER, Rafalski JA, Whitehorn EA, Baumeister K, Ivanoff L, Petteway SR Jr, Pearson ML, Lautenberger JA, Papas TS, Ghrayeb J, Chang NT, Gallo RC, Wong-Staal F**. Complete nucleotide sequence of the AIDS virus, HTLV-III. Nature 1985, 313:277-284.

36. **Re MC, Furlini G, Vignoli M, Ramazzotti E, Roderigo G, De Rosa V, Zauli G, Lolli S, Capitani S, La Placa M**. Effect of Antibody to HIV-1 Tat Protein on Viral Replication in Vitro and Progression of HIV-1 Disease in Vivo. J Acquired Immune Defic Syndr Hum Retrovirol 1995, 10:408-416.

37. **Re MC, Vignoli M, Furlini G, Gibellini D, Colangeli V, Vitone F, La Placa M.** Antibodies against full-lenght Tat protein and some low-molecular-weight Tat-peptides correlate with low or undetectable viral load in HIV-1 seropositive patients. J Clin Virol 2001, 21:81-89.

38. **Reiss P, Lange JM, de Ronde A, de Wolf F, Dekker J, Debouck C, Goudsmit J.** Speed of progression to AIDS and degree of antibody response to accessory gene products of HIV-1. J Med Virol 1990, 30:163-168.

39. **Rezza G, Fiorelli V, Dorrucci M, Ciccozzi M, Tripiciano A, Scoglio A, Collacchi B, Ruiz-Alvarez M, Giannetto C, Caputo A, Tomasoni L, Castelli F, Sciandra M, Sinicco A, Ensoli F, Butto S, Ensoli B.** The presence of anti-Tat antibodies is predictive of long-term nonprogression to AIDS or severe immunodeficiency: findings in a cohort of HIV-1 seroconverters. J Infect Dis 2005, 15:1321-1324.

40. **Rodman TC, To SE, Hashish H, Manchester K.** Epitopes for natural antibodies of human immunodeficiency virus (HIV)-negative (normal) and HIV-positive sera are

coincident with two key functional sequences of HIV Tat protein. Proc Natl Acad Sci USA 1993, 90:7719-7723.

41. **Shutt DC, Soll DR.** HIV-induced T-cell syncytia release a two component T-helper cell chemoattractant composed of Nef and Tat. J Cell Sci 1999, 112 (Pt 22):3931-3941.

42. **Smith DE, Walker BD, Cooper DA, Rosenberg ES, Kaldor JM**, Is antiretroviral treatment of primary HIV infection clinically justified on the basis of current evidence? AIDS 18, 709-718 (2004).

43. **Valdez H,Connick E, Smith KY, Lederman MM, Bosch RJ, Kim RS, St Clair M, Kuritzkes DR, Kessler H, Fox L, Blanchard-Vargas M, Landay A**, Limited immune restoration after 3 years' suppression of HIV-1 replication in patients with moderately advanced disease. AIDS 16, 1859-1866 (2002).

44. **Wahren B, Ljungberg K, Rollman E, Levi M, Zuber B, Kjerrstrom Zuber A, Hinkula J, Leandersson AC, Calarota S, Hejdeman B, Bratt G, Sandstrom E**. HIV subtypes and recombination strains--strategies for induction of immune responses in man. Vaccine 2002, 20(15):1988-1993.

45. **Wu Y, Marsh JW.** Selective transcription and modulation of resting T cell activity by preintegrated HIV DNA. Science 2001, 293:1503-1506.

46. **Zagury JF, Sill A, Blattner W, Lachgar A, Le Buanec H, Richardson M, Rappaport J, Hendel H, Bizzini B, Gringeri A, Carcagno M, Criscuolo M, Burny A, Gallo RC, Zagury D.** Antibodies to the HIV-1 Tat protein correlated with non progression to AIDS: a rationale for the use of Tat toxoid as an HIV-1 vaccine. J Hum Virol 1998, 1:282-292.

47. **Zagury D, Lachgar A, Chams V, Fall LS, Bernard J, Zagury JF, Bizzini B, Gringeri A, Santagostino E, Rappaport J, Feldman M, Burny A, Gallo RC**. Interferon alpha and Tat involvement in the immuno suppression of uninfected T cells and C-C chemokine decline in AIDS. Proc Natl Acad Sci USA 1998, 95:3851-3856.

48. **Dixon W.J., Massey F.J.,** Introduction to Statistical Analysis, 4th Edition McGraw-Hill, 1983.

49. **Fleiss J.L. Tytun A., Ury S.H.K.,** A simple approximation for calculating sample sizes for comparing independent proportions, Biometrics, 1980.

50. **Machin D., Campbell M.J.**, Statistical Tables for Design of Clinical Trials, Blackwell Scientific Publications, Oxford 1987.

20 APPENDIX I: TOXICITY TABLE

This toxicity table has been adapted for Protocol ISS T-003 from the Division of Aids table for grading the severity of Adult and Pediatric Adverse Events Version 1.0, December, 2004; clarification August 2009

**PARAMETER**	**GRADE 1 MILD**	**GRADE 2 MODERATE**	**GRADE 3 SEVERE**	**GRADE 4 POTENTIALLY LIFE-THREATENING**
**SYSTEMIC**				
Acute systemic allergic reaction	Localized urticaria (wheals) with no medical intervention indicated	Localized urticaria with medical intervention indicated OR Mild angioedema with no medical intervention indicated	Generalized urticaria OR Angioedema with medical intervention indicated OR Symptomatic mild bronchospasm	Acute anaphylaxis OR Life-threatening bronchospasm OR laryngeal edema
Chills	Symptoms causing no or minimal interference with usual social & functional activities	Symptoms causing greater than minimal interference with usual social & functional activities	Symptoms causing inability to perform usual social & functional activities	NA
Fatigue<br>Malaise	Symptoms causing no or minimal interference with usual social & functional activities	Symptoms causing greater than minimal interference with usual social & functional activities	Symptoms causing inability to perform usual social & functional activities	Incapacitating fatigue/ malaise symptoms causing inability to perform basic self-care functions
Fever<br>(non-axillary)	37.7 – 38.6°C	38.7 – 39.3°C	39.4 – 40.5°C	> 40.5°C
Pain<br>DO NOT use for pain due to injection (See Injection Site Reactions: Injection site pain)	Pain causing no or minimal interference with usual social & functional activities	Pain causing greater than minimal interference with usual social & functional activities	Pain causing inability to perform usual social & functional activities	Disabling pain causing inability to perform basic self-care functions OR Hospitalization (other than emergency room visit) indicated
Unintentional weight loss	NA	5 – 9% loss in body weight from baseline	10 – 19% loss in body weight from baseline	≥ 20% loss in body weight from baseline OR Aggressive intervention indicated [e.g., tube feeding or total parenteral nutrition (TPN)]

PARAMETER	GRADE 1<br>MILD	GRADE 2<br>MODERATE	GRADE 3<br>SEVERE	GRADE 4<br>POTENTIALLY LIFE-THREATENING
**INFECTION**				
Infection (any other than HIV infection)	Localized, no systemic antimicrobial treatment indicated AND Symptoms causing no or minimal interference with usual social & functional activities	Systemic antimicrobial treatment indicated OR Symptoms causing greater than minimal interference with usual social & functional activities	Systemic antimicrobial treatment indicated AND Symptoms causing inability to perform usual social & functional activities OR Operative intervention (other than simple incision and drainage) indicated	Life-threatening consequences (e.g., septic shock)
**INJECTION SITE REACTIONS**				
Injection site pain (pain without touching)<br>Or<br>Tenderness (pain when area is touched)	Pain/tenderness causing no or minimal limitation of use of limb	Pain/tenderness limiting use of limb OR Pain/tenderness causing greater than minimal interference with usual social & functional activities	Pain/tenderness causing inability to perform usual social & functional activities	Pain/tenderness causing inability to perform basic self-care function OR Hospitalization (other than emergency room visit) indicated for management of pain/tenderness
Injection site reaction (localized)	Erythema OR Induration of 5x5 cm – 9x9 cm (or 25 cm^2 – 81cm^2)	Erythema OR Induration OR Edema > 9 cm any diameter (or > 81 cm^2)	Ulceration OR Secondary infection OR Phlebitis OR Sterile abscess OR Drainage	Necrosis (involving dermis and deeper tissue)
Pruritis associated with injection	Itching localized to injection site AND Relieved spontaneously or with < 48 hours treatment	Itching beyond the injection site but not generalized OR Itching localized to injection site requiring ≥ 48 hours treatment	Generalized itching causing inability to perform usual social & functional activities	NA
**SKIN – DERMATOLOGICAL**				
Cutaneous reaction – rash	Localized macular rash	Diffuse macular, maculopapular, or morbilliform rash OR Target lesions	Diffuse macular, maculopapular, or morbilliform rash with vesicles or limited number of bullae OR Superficial ulcerations of mucous membrane limited to one site	Extensive or generalized bullous lesions OR Stevens-Johnson syndrome OR Ulceration of mucous membrane involving two or more distinct mucosal sites OR Toxic epidermal necrolysis (TEN)
Hyperpigmentation	Slight or localized	Marked or generalized	NA	NA
Hypopigmentation	Slight or localized	Marked or generalized	NA	NA

**PARAMETER**	**GRADE 1 MILD**	**GRADE 2 MODERATE**	**GRADE 3 SEVERE**	**GRADE 4 POTENTIALLY LIFE-THREATENING**
Pruritis (itching – no skin lesions)	Itching causing no or minimal interference with usual social & functional activities	Itching causing greater than minimal interference with usual social & functional activities	Itching causing inability to perform usual social & functional activities	NA
**CARDIOVASCULAR**				
Cardiac arrhythmia (general)<br>(By ECG or physical exam)	Asymptomatic AND No intervention indicated	Asymptomatic AND Non-urgent medical intervention indicated	Symptomatic, non-life-threatening AND Non-urgent medical intervention indicated	Life-threatening arrhythmia OR Urgent intervention indicated
Hemorrhage (significant acute blood loss)	NA	Symptomatic AND No transfusion indicated	Symptomatic AND Transfusion of ≤ 2 units packed RBCs (for children ≤ 10 cc/kg) indicated	Life-threatening hypotension OR Transfusion of > 2 units packed RBCs (for children > 10 cc/kg) indicated
Hypertension	140 – 159 mmHg systolic<br>OR<br>90 – 99 mmHg diastolic	160 – 179 mmHg systolic<br>OR<br>100 – 109 mmHg diastolic	≥ 180 mmHg systolic<br>OR<br>≥ 110 mmHg diastolic	Life-threatening consequences (e.g., malignant hypertension) OR Hospitalization indicated (other than emergency room visit)
Hypotension	NA	Symptomatic, corrected with oral fluid replacement	Symptomatic, IV fluids indicated	Shock requiring use of vasopressors or mechanical assistance to maintain blood pressure
Pericardial effusion	Asymptomatic, small effusion requiring no intervention	Asymptomatic, moderate or larger effusion requiring no intervention	Effusion with non-life threatening physiologic consequences OR Effusion with non-urgent intervention indicated	Life-threatening consequences (e.g., tamponade) OR Urgent intervention indicated
Vasovagal episode (associated with a procedure of any kind)	Present without loss of consciousness	Present with transient loss of consciousness	NA	NA
**GASTRO-INTESTINAL**				
Constipation	NA	Persistent constipation requiring regular use of dietary modifications, laxatives, or enemas	Obstipation with manual evacuation indicated	Life-threatening consequences (e.g., obstruction)

PARAMETER	GRADE 1<br>MILD	GRADE 2<br>MODERATE	GRADE 3<br>SEVERE	GRADE 4<br>POTENTIALLY LIFE-THREATENING
Diarrhea	Transient or intermittent episodes of unformed stools OR Increase of ≤ 3 stools over baseline per 24-hour period	Persistent episodes of unformed to watery stools OR Increase of 4 – 6 stools over baseline per 24-hour period	Bloody diarrhea OR Increase of ≥ 7 stools per 24-hour period OR IV fluid replacement indicated	Life-threatening consequences (e.g., hypotensive shock)
Dysphagia-Odynophagia	Symptomatic but able to eat usual diet	Symptoms causing altered dietary intake without medical intervention indicated	Symptoms causing severely altered dietary intake with medical intervention indicated	Life-threatening reduction in oral intake
Nausea	Transient (< 24 hours) or intermittent nausea with no or minimal interference with oral intake	Persistent nausea resulting in decreased oral intake for 24 – 48 hours	Persistent nausea resulting in minimal oral intake for > 48 hours OR Aggressive rehydration indicated (e.g., IV fluids)	Life-threatening consequences (e.g., hypotensive shock)
Vomiting	Transient or intermittent vomiting with no or minimal interference with oral intake	Frequent episodes of vomiting with no or mild dehydration	Persistent vomiting resulting in orthostatic hypotension OR Aggressive rehydration indicated (e.g., IV fluids)	Life-threatening consequences (e.g., hypotensive shock)
**NEUROLOGIC**				
Alteration in personality-behavior or in mood (e.g., agitation, anxiety, depression, mania, psychosis)	Alteration causing no or minimal interference with usual social & functional activities	Alteration causing greater than minimal interference with usual social & functional activities	Alteration causing inability to perform usual social & functional activities	Behavior potentially harmful to self or others (e.g., suicidal and homicidal ideation or attempt, acute psychosis) OR Causing inability to perform basic self-care functions
Ataxia	Asymptomatic ataxia detectable on exam OR Minimal ataxia causing no or minimal interference with usual social & functional activities	Symptomatic ataxia causing greater than minimal interference with usual social & functional activities	Symptomatic ataxia causing inability to perform usual social & functional activities	Disabling ataxia causing inability to perform basic self-care functions

PARAMETER	GRADE 1<br>MILD	GRADE 2<br>MODERATE	GRADE 3<br>SEVERE	GRADE 4<br>POTENTIALLY LIFE-THREATENING
Headache	Symptoms causing no or minimal interference with usual social & functional activities	Symptoms causing greater than minimal interference with usual social & functional activities	Symptoms causing inability to perform usual social & functional activities	Symptoms causing inability to perform basic self-care functions OR Hospitalization indicated (other than emergency room visit) OR Headache with significant impairment of alertness or other neurologic function
Insomnia	NA	Difficulty sleeping causing greater than minimal interference with usual social & functional activities	Difficulty sleeping causing inability to perform usual social & functional activities	Disabling insomnia causing inability to perform basic self-care functions
Neuromuscular weakness (including myopathy & neuropathy)	Asymptomatic with decreased strength on exam OR Minimal muscle weakness causing no or minimal interference with usual social & functional activities	Muscle weakness causing greater than minimal interference with usual social & functional activities	Muscle weakness causing inability to perform usual social & functional activities	Disabling muscle weakness causing inability to perform basic self-care functions OR Respiratory muscle weakness impairing ventilation
Neurosensory alteration (including paresthesia and painful neuropathy)	Asymptomatic with sensory alteration on exam or minimal paresthesia causing no or minimal interference with usual social & functional activities	Sensory alteration or paresthesia causing greater than minimal interference with usual social & functional activities	Sensory alteration or paresthesia causing inability to perform usual social & functional activities	Disabling sensory alteration or paresthesia causing inability to perform basic self-care functions
Syncope (not associated with a procedure)	NA	Present	NA	NA
Vertigo	Vertigo causing no or minimal interference with usual social & functional activities	Vertigo causing greater than minimal interference with usual social & functional activities	Vertigo causing inability to perform usual social & functional activities	Disabling vertigo causing inability to perform basic self-care functions
**RESPIRATORY**				
Bronchospasm (acute)	FEV1 or peak flow reduced to 70 – 80%	FEV1 or peak flow 50 – 69%	FEV1 or peak flow 25 – 49%	Cyanosis OR FEV1 or peak flow < 25% OR Intubation

PARAMETER	GRADE 1 MILD	GRADE 2 MODERATE	GRADE 3 SEVERE	GRADE 4 POTENTIALLY LIFE-THREATENING
Dyspnea or respiratory distress	Dyspnea on exertion with no or minimal interference with usual social & functional activities	Dyspnea on exertion causing greater than minimal interference with usual social & functional activities	Dyspnea at rest causing inability to perform usual social & functional activities	Respiratory failure with ventilatory support indicated
**MUSCULOSKELETAL**				
Arthralgia<br>See also Arthritis	Joint pain causing no or minimal interference with usual social & functional activities	Joint pain causing greater than minimal interference with usual social & functional activities	Joint pain causing inability to perform usual social & functional activities	Disabling joint pain causing inability to perform basic self-care functions
Arthritis<br>See also Arthralgia	Stiffness or joint swelling causing no or minimal interference with usual social & functional activities	Stiffness or joint swelling causing greater than minimal interference with usual social & functional activities	Stiffness or joint swelling causing inability to perform usual social & functional activities	Disabling joint stiffness or swelling causing inability to perform basic self-care functions
Myalgia<br>(non-injection site)	Muscle pain causing no or minimal interference with usual social & functional activities	Muscle pain causing greater than minimal interference with usual social & functional activities	Muscle pain causing inability to perform usual social & functional activities	Disabling muscle pain causing inability to perform basic self-care functions
**OCULAR/VISUAL**				
Visual changes (from baseline)	Visual changes causing no or minimal interference with usual social & functional activities	Visual changes causing greater than minimal interference with usual social & functional activities	Visual changes causing inability to perform usual social & functional activities	Disabling visual loss in affected eye(s)
**HEMATOLOGY**	***Standard International Units are listed in italics***			
Absolute neutrophil count (ANC)	1,000 – 1,300/mm^3<br>*1.000 x 10^9 – 1.300 x 10^9/L*	750 – 999/mm^3<br>*0.750 x 10^9 – 0.999 x 10^9/L*	500 – 749/mm^3<br>*0.500 x 10^9 – 0.749 x 10^9/L*	< 500/mm^3<br>*< 0.500 x 10^9/L*
Fibrinogen, decreased	100 – 200 mg/dL<br>*1.00 – 2.00 g/L*<br>OR<br>0.75 – 0.99 x LLN	75 – 99 mg/dL<br>*0.75 – 0.99 g/L*<br>OR<br>0.50 – 0.74 x LLN	50 – 74 mg/dL<br>*0.50 – 0.74 g/L*<br>OR<br>0.25 – 0.49 x LLN	< 50 mg/dL<br>*< 0.50 g/L*<br>OR<br>< 0.25 x LLN<br>OR<br>Associated with gross bleeding
Hemoglobin (Hgb)<br>(HIV POSITIVE ONLY)	8.5 – 10.0 g/dL<br>*5.24 – 6.23 mmol/L*	7.5 – 8.4 g/dL<br>*4.62–5.23 mmol/L*	6.50 – 7.4 g/dL<br>*4.03–4.61 mmol/L*	< 6.5 g/dL<br>*< 4.03 mmol/L*
International Normalized Ratio of prothrombin time (INR)	1.1 – 1.5 x ULN	1.6 – 2.0 x ULN	2.1 – 3.0 x ULN	> 3.0 x ULN
Methemoglobin	5.0 – 10.0%	10.1 – 15.0%	15.1 – 20.0%	> 20.0%
Prothrombin Time (PT)	1.1 – 1.25 x ULN	1.26 – 1.50 x ULN	1.51 – 3.00 x ULN	> 3.00 x ULN

**PARAMETER**	**GRADE 1**<br>**MILD**	**GRADE 2**<br>**MODERATE**	**GRADE 3**<br>**SEVERE**	**GRADE 4**<br>**POTENTIALLY LIFE-THREATENING**
Partial Thromboplastin Time (PTT)	1.1 – 1.66 x ULN	1.67 – 2.33 x ULN	2.34 – 3.00 x ULN	> 3.00 x ULN
Platelets, decreased	100,000 – 124,999/mm^3<br>*100.000 x 10^9 – 124.999 x 10^9/L*	50,000 – 99,999/mm^3<br>*50.000 x 10^9 – 99.999 x 10^9/L*	25,000 – 49,999/mm^3<br>*25.000 x 10^9 – 49.999 x 10^9/L*	< 25,000/mm^3<br>*< 25.000 x 10^9/L*
WBC, decreased	2,000 – 2,500/mm^3<br>*2.000 x 10^9 – 2.500 x 10^9/L*	1,500 – 1,999/mm^3<br>*1.500 x 10^9 – 1.999 x 10^9/L*	1,000 – 1,499/mm^3<br>*1.000 x 10^9 – 1.499 x 10^9/L*	< 1,000/mm^3<br>*< 1.000 x 10^9/L*
**CHEMISTRIES**	***Standard International Units are listed in italics***			
Albumin, serum, low	3.0 g/dL – < LLN<br>*30 g/L – < LLN*	2.0 – 2.9 g/dL<br>*20 – 29 g/L*	< 2.0 g/dL<br>*< 20 g/L*	NA
Alkaline Phosphatase	1.25 – 2.5 x ULN[†]	2.6 – 5.0 x ULN[†]	5.1 – 10.0 x ULN[†]	> 10.0 x ULN[†]
ALT (SGPT)	1.25 – 2.5 x ULN	2.6 – 5.0 x ULN	5.1 – 10.0 x ULN	> 10.0 x ULN
AST (SGOT)	1.25 – 2.5 x ULN	2.6 – 5.0 x ULN	5.1 – 10.0 x ULN	> 10.0 x ULN
Bicarbonate, serum, low	16.0 mEq/L – < LLN<br>*16.0 mmol/L – < LLN*	11.0 – 15.9 mEq/L<br>*11.0 – 15.9 mmol/L*	8.0 – 10.9 mEq/L<br>*8.0 – 10.9 mmol/L*	< 8.0 mEq/L<br>*< 8.0 mmol/L*
Bilirubin (Total)	1.1 – 1.5 x ULN	1.6 – 2.5 x ULN	2.6 – 5.0 x ULN	> 5.0 x ULN
Calcium, serum, high (corrected for albumin)	10.6 – 11.5 mg/dL<br>*2.65 – 2.88 mmol/L*	11.6 – 12.5 mg/dL<br>*2.89 – 3.13 mmol/L*	12.6 – 13.5 mg/dL<br>*3.14 – 3.38 mmol/L*	> 13.5 mg/dL<br>*> 3.38 mmol/L*
Calcium, serum, low (corrected for albumin)	7.8 – 8.4 mg/dL<br>*1.95 – 2.10 mmol/L*	7.0 – 7.7 mg/dL<br>*1.75 – 1.94 mmol/L*	6.1 – 6.9 mg/dL<br>*1.53 – 1.74 mmol/L*	< 6.1 mg/dL<br>*< 1.53 mmol/L*
Creatine Kinase	3.0 – 5.9 x ULN[†]	6.0 – 9.9 x ULN[†]	10.0 – 19.9 x ULN[†]	≥ 20.0 x ULN[†]
Creatinine	1.1 – 1.3 x ULN[†]	1.4 – 1.8 x ULN[†]	1.9 – 3.4 x ULN[†]	≥ 3.5 x ULN[†]
Glucose, serum, high (Non-fasting)	116 – 160 mg/dL<br>*6.44 – 8.88 mmol/L*	161 – 250 mg/dL<br>*8.89 – 13.88 mmol/L*	251 – 500 mg/dL<br>*13.89 – 27.75 mmol/L*	> 500 mg/dL<br>*> 27.75 mmol/L*
Glucose, serum, low	55 – 64 mg/dL<br>*3.05 – 3.55 mmol/L*	40 – 54 mg/dL<br>*2.22 – 3.06 mmol/L*	30 – 39 mg/dL<br>*1.67 – 2.23 mmol/L*	< 30 mg/dL<br>*< 1.67 mmol/L*
Lactate	ULN - < 2.0 x ULN without acidosis	≥ 2.0 x ULN without acidosis	Increased lactate with pH < 7.3 without life-threatening consequences	Increased lactate with pH < 7.3 with life-threatening consequences
Lipase	1.1 – 1.5 x ULN	1.6 – 3.0 x ULN	3.1 – 5.0 x ULN	> 5.0 x ULN
Magnesium, serum, low	1.2 – 1.4 mEq/L<br>*0.60 – 0.70 mmol/L*	0.9 – 1.1 mEq/L<br>*0.45 – 0.59 mmol/L*	0.6 – 0.8 mEq/L<br>*0.30 – 0.44 mmol/L*	< 0.60 mEq/L<br>*< 0.30 mmol/L*
Pancreatic amylase	1.1 – 1.5 x ULN	1.6 – 2.0 x ULN	2.1 – 5.0 x ULN	> 5.0 x ULN
Phosphate, serum, low	2.5 mg/dL – < LLN<br>*0.81 mmol/L – < LLN*	2.0 – 2.4 mg/dL<br>*0.65 – 0.80 mmol/L*	1.0 – 1.9 mg/dL<br>*0.32 – 0.64 mmol/L*	< 1.00 mg/dL<br>*< 0.32 mmol/L*
Potassium, serum, high	5.6 – 6.0 mEq/L<br>*5.6 – 6.0 mmol/L*	6.1 – 6.5 mEq/L<br>*6.1 – 6.5 mmol/L*	6.6 – 7.0 mEq/L<br>*6.6 – 7.0 mmol/L*	> 7.0 mEq/L<br>*> 7.0 mmol/L*
Potassium, serum, low	3.0 – 3.4 mEq/L<br>*3.0 – 3.4 mmol/L*	2.5 – 2.9 mEq/L<br>*2.5 – 2.9 mmol/L*	2.0 – 2.4 mEq/L<br>*2.0 – 2.4 mmol/L*	< 2.0 mEq/L<br>*< 2.0 mmol/L*
Sodium, serum, high	146 – 150 mEq/L<br>*146 – 150 mmol/L*	151 – 154 mEq/L<br>*151 – 154 mmol/L*	155 – 159 mEq/L<br>*155 – 159 mmol/L*	≥ 160 mEq/L<br>*≥ 160 mmol/L*
Sodium, serum, low	130 – 135 mEq/L<br>*130 – 135 mmol/L*	125 – 129 mEq/L<br>*125 – 129 mmol/L*	121 – 124 mEq/L<br>*121 – 124 mmol/L*	≤ 120 mEq/L<br>*≤ 120 mmol/L*

**PARAMETER**	**GRADE 1 MILD**	**GRADE 2 MODERATE**	**GRADE 3 SEVERE**	**GRADE 4 POTENTIALLY LIFE-THREATENING**
Triglycerides (fasting)	NA	500 – 750 mg/dL<br>*5.65 – 8.48 mmol/L*	751 – 1,200 mg/dL<br>*8.49 – 13.56 mmol/L*	> 1,200 mg/dL<br>*> 13.56 mmol/L*
Uric acid	7.5 – 10.0 mg/dL<br>*0.45 – 0.59 mmol/L*	10.1 – 12.0 mg/dL<br>*0.60 – 0.71 mmol/L*	12.1 – 15.0 mg/dL<br>*0.72 – 0.89 mmol/L*	> 15.0 mg/dL<br>*> 0.89 mmol/L*
**URINALYSIS**				
Hematuria (microscopic)	6 – 10 RBC/HPF	> 10 RBC/HPF	Gross, with or without clots OR with RBC casts	Transfusion indicated
Proteinuria, random collection	1 +	2 – 3 +	4 +	NA
Proteinuria, 24 hr collection	200 – 999 mg/24 h<br>*0.200 – 0.999 g/d*	1,000 – 1,999 mg/24 h<br>*1.000 – 1.999 g/d*	2,000 – 3,500 mg/24 h<br>*2.000 – 3.500 g/d*	> 3,500 mg/24 h<br>*> 3.500 g/d*

† Use age and sex appropriate values (e.g., bilirubin).

21 APPENDIX II: SCHEDULE OF PROTOCOL VISITS & PROCEDURES

**Week**	**-5**	**0**	**1**	**4**	**5**	**8**	**9**	**12**	**16**	**20**	**24**	**48**	**55**
**Day**	**-35**	**0**	**7**	**28**	**35**	**56**	**63**	**84**	**112**	**140**	**168**	**336**	**343**
**Study Visit**	**1**	**2**	**3**	**4**	**5**	**6**	**7**	**8**	**9**	**10**	**11**	**12**	
Vaccination		x		x		x							
Informed consent	x												
Medical history	x	x[1]											
Eligibility verification	x	x		x		x							
Physical examination	x	x		x		x		x	x	x	x	x	
Chest X-ray[2]	x												
Vital signs[3]	x	x	x	x	x	x	x	x	x	x	x	x	
Serum pregnancy[4]	x												
Urine pregnancy[5]		x		x		x						x	
Haematology	x	x	x		x		x	x	x	x	x	x	
Clinical Chemistry	x	x	x		x		x	x	x	x	x	x	
Coagulation	x	x	x		x		x	x	x	x	x	x	
Urinalysis[6]	x	x	x		x		x	x	x	x	x	x	
Thyroid function (T4 & TSH)	x												
HIV-1/2 ELISA	x												
CD4	x	x		x		x		x	x	x	x	x	
HIV-1 plasma viraemia	x	x		x		x		x	x	x	x	x	
Anti-tat antibodies (IgG, IgM, IgA)	x	x		x		x		x	x	x	x	x	
Blood sample collection & storage for immunological & virological analyses		x		x		x		x	x	x	x	x	
Cervical swab and Pap smear collection (Females only)		x								x		x	
Counselling[7]	x	x		x		x		x	x	x	x	x	
Diary Card issue		x		x		x							
Diary card collection & review			x		x		x						
Adverse events		x	x	x	x	x	x	x	x	x	x	x	
Concomitant medication	x	x	x	x	x	x	x	x	x	x	x	x	
Telephonic contact with subject[8]													x

1 Medical history to be reviewed at Visit 2 prior to the first vaccination
2 If no chest x-ray available within 6 months of screening period
3 After 5 minutes in a resting position (seated blood pressure, heart rate, respiratory rate and axillary body temperature)
4 All females
5 Females of child-bearing potential only
6 The same sample of urine to be submitted for microscopy and culture if dipstick results are abnormal and clinically significant
7 Counselling: Pre- and post HIV test at screening, and ARV adherence, risk reduction and prevention of pregnancy at other designated visits
8. Site to contact subject telephonically to give results of the following tests done during Visit 12: haematology, clinical chemistry, coagulation and urinalysis assessments CD4+ T cell counts, HIV-1 plasma viraemia. Pap smear results will be communicated as soon as they become available. Subject will be requested to come to the site if there is a need for a referral to a health facility.

22 APPENDIX III: DECLARATION OF HELSINKI

WORLD MEDICAL ASSOCIATION DECLARATION OF HELSINKI
Ethical Principles for Medical Research Involving Human Subjects
Adopted by the 18th WMA General Assembly, Helsinki, Finland, June 1964, and amended by the:
29th WMA General Assembly, Tokyo, Japan, October 1975
35th WMA General Assembly, Venice, Italy, October 1983
41st WMA General Assembly, Hong Kong, September 1989
48th WMA General Assembly, Somerset West, Republic of South Africa, October 1996
52nd WMA General Assembly, Edinburgh, Scotland, October 2000
53th WMA General Assembly, Washington 2002 (Note of Clarification on paragraph 29 added)
55th WMA General Assembly, Tokyo 2004 (Note of Clarification on Paragraph 30 added)
59th WMA General Assembly, Seoul, October 2008

A. INTRODUCTION
1. The World Medical Association (WMA) has developed the Declaration of Helsinki as a statement of ethical principles for medical research involving human subjects**,** including research on identifiable human material and data. The Declaration is intended to be read as a whole and each of its constituent paragraphs should not be applied without consideration of all other relevant paragraphs.
2. Although the Declaration is addressed primarily to physicians, the WMA encourages other participants in medical research involving human subjects to adopt these principles.
3. It is the duty of the physician to promote and safeguard the health of patients, including those who are involved in medical research. The physician's knowledge and conscience are dedicated to the fulfilment of this duty.
4. The Declaration of Geneva of the WMA binds the physician with the words, “The health of my patient will be my first consideration,” and the International Code of Medical Ethics declares that, “A physician shall act in the patient's best interest when providing medical care.”
5. Medical progress is based on research that ultimately must include studies involving human subjects. Populations that are underrepresented in medical research should be provided appropriate access to participation in research.
6. In medical research involving human subjects, the well-being of the individual research subject must take precedence over all other interests.
7. The primary purpose of medical research involving human subjects is to understand the causes, development and effects of diseases and improve preventive, diagnostic and therapeutic interventions (methods, procedures and treatments). Even the best current interventions must be evaluated continually through research for their safety, effectiveness, efficiency, accessibility and quality.
8. In medical practice and in medical research, most interventions involve risks and burdens.
9. Medical research is subject to ethical standards that promote respect for all human subjects and protect their health and rights. Some research populations are particularly vulnerable and need special protection. These include those who cannot give or refuse consent for themselves and those who may be vulnerable to coercion or undue influence.
10. Physicians should consider the ethical, legal and regulatory norms and standards for research involving human subjects in their own countries as well as applicable international norms and standards. No national or international ethical, legal or regulatory requirement should reduce or eliminate any of the protections for research subjects set forth in this Declaration.

B. PRINCIPLES FOR ALL MEDICAL RESEARCH
11. It is the duty of physicians who participate in medical research to protect the life, health, dignity, integrity, right to self-determination, privacy, and confidentiality of personal information of research subjects.
12. Medical research involving human subjects must conform to generally accepted scientific principles, be based on a thorough knowledge of the scientific literature, other relevant sources of information, and adequate laboratory and, as appropriate, animal experimentation. The welfare of animals used for research must be respected.
13. Appropriate caution must be exercised in the conduct of medical research that may harm the environment.
14. The design and performance of each research study involving human subjects must be clearly described in a research protocol. The protocol should contain a statement of the ethical considerations involved and should indicate how the principles in this Declaration have been addressed. The protocol should include information regarding funding, sponsors, institutional affiliations, other potential conflicts of interest, incentives for subjects

and provisions for treating and/or compensating subjects who are harmed as a consequence of participation in the research study. The protocol should describe arrangements for post-study access by study subjects to interventions identified as beneficial in the study or access to other appropriate care or benefits.
15. The research protocol must be submitted for consideration, comment, guidance and approval to a research ethics committee before the study begins. This committee must be independent of the researcher, the sponsor and any other undue influence. It must take into consideration the laws and regulations of the country or countries in which the research is to be performed as well as applicable international norms and standards but these must not be allowed to reduce or eliminate any of the protections for research subjects set forth in this Declaration. The committee must have the right to monitor ongoing studies. The researcher must provide monitoring information to the committee, especially information about any serious adverse events. No change to the protocol may be made without consideration and approval by the committee.
16. Medical research involving human subjects must be conducted only by individuals with the appropriate scientific training and qualifications. Research on patients or healthy volunteers requires the supervision of a competent and appropriately qualified physician or other health care professional. The responsibility for the protection of research subjects must always rest with the physician or other health care professional and never the research subjects, even though they have given consent.
17. Medical research involving a disadvantaged or vulnerable population or community is only justified if the research is responsive to the health needs and priorities of this population or community and if there is a reasonable likelihood that this population or community stands to benefit from the results of the research.
18. Every medical research study involving human subjects must be preceded by careful assessment of predictable risks and burdens to the individuals and communities involved in the research in comparison with foreseeable benefits to them and to other individuals or communities affected by the condition under investigation.
19. Every clinical trial must be registered in a publicly accessible database before recruitment of the first subject.
20. Physicians may not participate in a research study involving human subjects unless they are confident that the risks involved have been adequately assessed and can be satisfactorily managed. Physicians must immediately stop a study when the risks are found to outweigh the potential benefits or when there is conclusive proof of positive and beneficial results.
21. Medical research involving human subjects may only be conducted if the importance of the objective outweighs the inherent risks and burdens to the research subjects.
22. Participation by competent individuals as subjects in medical research must be voluntary. Although it may be appropriate to consult family members or community leaders, no competent individual may be enrolled in a research study unless he or she freely agrees.
23. Every precaution must be taken to protect the privacy of research subjects and the confidentiality of their personal information and to minimize the impact of the study on their physical, mental and social integrity.
24. In medical research involving competent human subjects, each potential subject must be adequately informed of the aims, methods, sources of funding, any possible conflicts of interest, institutional affiliations of the researcher, the anticipated benefits and potential risks of the study and the discomfort it may entail, and any other relevant aspects of the study. The potential subject must be informed of the right to refuse to participate in the study or to withdraw consent to participate at any time without reprisal. Special attention should be given to the specific information needs of individual potential subjects as well as to the methods used to deliver the information. After ensuring that the potential subject has understood the information, the physician or another appropriately qualified individual must then seek the potential subject's freely-given informed consent, preferably in writing. If the consent cannot be expressed in writing, the non-written consent must be formally documented and witnessed.
25. For medical research using identifiable human material or data, physicians must normally seek consent for the collection, analysis, storage and/or reuse. There may be situations where consent would be impossible or impractical to obtain for such research or would pose a threat to the validity of the research. In such situations the research may be done only after consideration and approval of a research ethics committee.
26. When seeking informed consent for participation in a research study the physician should be particularly cautious if the potential subject is in a dependent relationship with the physician or may consent under duress. In such situations the informed consent should be sought by an appropriately qualified individual who is completely independent of this relationship.
27. For a potential research subject who is incompetent, the physician must seek informed consent from the legally authorized representative. These individuals must not be included in a research study that has no likelihood of benefit for them unless it is intended to promote the health of the population represented by the potential subject, the research cannot instead be performed with competent persons, and the research entails only minimal risk and minimal burden.

28. When a potential research subject who is deemed incompetent is able to give assent to decisions about participation in research, the physician must seek that assent in addition to the consent of the legally authorized representative. The potential subject's dissent should be respected.
29. Research involving subjects who are physically or mentally incapable of giving consent, for example, unconscious patients, may be done only if the physical or mental condition that prevents giving informed consent is a necessary characteristic of the research population. In such circumstances the physician should seek informed consent from the legally authorized representative. If no such representative is available and if the research cannot be delayed, the study may proceed without informed consent provided that the specific reasons for involving subjects with a condition that renders them unable to give informed consent have been stated in the research protocol and the study has been approved by a research ethics committee. Consent to remain in the research should be obtained as soon as possible from the subject or a legally authorized representative.
30. Authors, editors and publishers all have ethical obligations with regard to the publication of the results of research. Authors have a duty to make publicly available the results of their research on human subjects and are accountable for the completeness and accuracy of their reports. They should adhere to accepted guidelines for ethical reporting. Negative and inconclusive as well as positive results should be published or otherwise made publicly available. Sources of funding, institutional affiliations and conflicts of interest should be declared in the publication. Reports of research not in accordance with the principles of this Declaration should not be accepted for publication.

C. ADDITIONAL PRINCIPLES FOR MEDICAL RESEARCH COMBINED WITH MEDICAL CARE

31. The physician may combine medical research with medical care only to the extent that the research is justified by its potential preventive, diagnostic or therapeutic value and if the physician has good reason to believe that participation in the research study will not adversely affect the health of the patients who serve as research subjects.
32. The benefits, risks, burdens and effectiveness of a new intervention must be tested against those of the best current proven intervention, except in the following circumstances:
• The use of placebo, or no treatment, is acceptable in studies where no current proven intervention exists; or
• Where for compelling and scientifically sound methodological reasons the use of placebo is necessary to determine the efficacy or safety of an intervention and the patients who receive placebo or no treatment will not be subject to any risk of serious or irreversible harm. Extreme care must be taken to avoid abuse of this option.
33. At the conclusion of the study, patients entered into the study are entitled to be informed about the outcome of the study and to share any benefits that result from it, for example, access to interventions identified as beneficial in the study or to other appropriate care or benefits.
34. The physician must fully inform the patient which aspects of the care are related to the research. The refusal of a patient to participate in a study or the patient's decision to withdraw from the study must never interfere with the patient-physician relationship.
35. In the treatment of a patient, where proven interventions do not exist or have been ineffective, the physician, after seeking expert advice, with informed consent from the patient or a legally authorized representative, may use an unproven intervention if in the physician's judgement it offers hope of saving life, re-establishing health or alleviating suffering. Where possible, this intervention should be made the object of research, designed to evaluate its safety and efficacy. In all cases, new information should be recorded and, where appropriate, made publicly available.

23 APPENDIX IV: GLOSSARY

Abs	Absorbance
ADCC	Antibody-mediated Cellular Cytotoxicity
ADR	Adverse Drug Reaction
AE	Adverse Event
Ag	Antigen
AIDS	Acquired Immune Deficiency Syndrome
ALT	Alanine amino Transferase
ANOVA	Analysis of Variance
APCs	Antigen-Presenting Cells
APTT	Activated Partial Thromboplastin Time
AST	Aspartate amino Transferase
AVIP	AIDS Vaccine Integrated Programme
BAER	Brainstem Auditory Evoked Response
CAB	Community Advisory Board
CCR-5	Chemokine Receptor 5
CD	Cluster of Differentiation
CDC	Center of Disease Control
CEC	Central Ethical Committee
CFSE	CarboxyFluoroscein Succinimidyl Ester
CL-CRF	Core Laboratory Case Report Form
cm	Centimeter
CNS	Central Nervous System
CRF	Case Report Form
CRO	Contract Research Organization
CSF	CerebroSpinal Fluid
CTLs	Cytotoxic T Lymphocytes
CTM	Clinical Trial Monitor
DEAE	Diethylaminoethyl
dl	Deciliter
DNA	Deoxyribonucleic Acid
DSMB	Data Safety Monitoring Board
e-CRF	Electronic Case Report Form
ELISA	Enzyme-Linked Immunosorbent Assay
EudraCT	European Clinical Trials DatabaseEuropea
FP6	Framework Programme 6
g	Gram
GCP	Good Clinical Practice
GGT (γGT)	Gamma Glutamyl Transferase
GMP	Good Manufacturing Practice
GMT	Geometric Mean Titers
HAART	Highly Active Antiretroviral Therapy
Hb	Hemoglobin
Hbe Ag	Hepatitis B e Antigen
Hbs Ag	Hepatitis B surface Antigen
HBV	Hepatitis B Virus
HCT	Hematocrit
HCV	Hepatitis C Virus

HHV-8	Human Herpes Virus 8
HIV-1	Human Immunodeficiency Virus type 1
HIV-1 LTR	Human Immunodeficiency Virus type 1 Long Terminal Repeat
HIV-2	Human Immunodeficiency Virus type 2
HLA	Human Leukocyte Antigen
HPLC	High Performance Liquid Chromatography
HTLV-I	Human T-Lymphotropic Virus I
HTLV-II	Human T-Lymphotropic Virus II
HTLV-III	Human T-Lymphotropic Virus III
HSA	Human Serum Albumin
ICAV	Italian Concerted Action on HIV/AIDS Vaccine Development
ICH	International Conference on Harmonization
ICS	Intracellular Cytokine Staining
ID	Identification number
id	Intradermally
IFO	Istituti Fisioterapici Ospitalieri
IgA	Immunoglobulin A
IgE	Immunoglobulin E
IgG	Immunoglobulin G
IgM	Immunoglobulin M
IL-2	Interleukin-2
IL-4	Interleukin-4
IL-12	Interleukin-12
INMI	Istituto Nazionale Malattie Infettive
ISS	Istituto Superiore di Sanità
Kg	Kilogram
LEC	Local Ethical Committee
MCV	Mean Corpuscolar Volume
MDDCs	Monocyte-derived dendritic cells
MedDRA	Medical Dictionary for Regulatory Activities
mg	Milligram
MHC	Major Histocompatibility Complex
MID_{50}	Monkey 50% Infectious Doses
min	Minute
MIP-1α	Macrophage Inflammatory Protein 1α
MIP-1β	Macrophage Inflammatory Protein-1β
ml	Millilitre
mm^3	Millimetre cube
NA	Neutralizing Antibodies
ng	Nanogram
NIH	National Institutes of Health
NK	Natural Killer
nm	Nanometre
NMR	Nuclear Magnetic Resonance
PBMC	Peripheral Blood Mononuclear Cell
PPD	Purified Protein Derivative
RANTES	Regulated on Activation Normal T cell Expressed and Secreted
RBC	Red blood cell
RD	Relationship to Drug
RNA	Ribonucleic Acid

RPR	Rapid Plasma Reagin
SADR	Serious Adverse Drug Reaction
SAE	Serious Adverse Event
SAS	Statistical and data Analysis Software
sc	Subcutaneous
SHIV	Simian/Human Immunodeficiency Virus
SIV	Simian Immunodeficiency Virus
SOC	System Organ Classes
SOP	Standard Operating Procedure
SUSAR	Suspected Unexpected Serious Adverse Reaction
T4	Thyroxine
Th1	Type 1 helper T Cells
Th2	Type 2 helper T Cells
TM	Trade Mark
TNF-α	Tumor Necrosis Factor-α
TSH	Thyroid-stimulating Hormone
UI	International Unit
WBC	White Blood Cell
WHO	World Health Organization
WMA	World Medical Association
**γIFN**	**γ Interferon**
μg	Microgram
μl	Microliter
°C	Temperature in degrees Celsius
