# Supplementary material for: Therapeutic HIV-1 Tat vaccination promotes durable immune reconstitution and reservoir reduction in ART-treated adults with clade C infection: a 12-year follow-up study
Source: Front Immunol. 2026 Apr 10;17:1769223. doi: 10.3389/fimmu.2026.1769223 (PMC13106519; doi:10.3389/fimmu.2026.1769223)
Supplement: Supplementary file 1 [file DataSheet1.zip › Data Sheet1/Supplementary figures and tables.docx]

**Supplementary Table 1a. Characteristics of males by treatment group at the ISS T-003 trial baseline**

|  | **n** | **Tat vaccine** | **n** | **Placebo** | **p value** |
| --- | --- | --- | --- | --- | --- |
|  | 28 |  | 14 |  |  |
| **Age** |  |  |  |  |  |
| Mean+ SD | 28 | 37+ 6 | 14 | 36+7 | 0.4903^++^ |
|  |  |  |  |  |  |
| **Years from HIV diagnosis** |  |  |  |  |  |
| Mean+ SD | 28 | 4+ 2 | 14 | 4+ 2 | 0.3091^++^ |
|  |  |  |  |  |  |
| **Years from cART initiation** |  |  |  |  |  |
| Mean+ SD | 28 | 4+ 2 | 14 | 3+ 2 | 0.3725^++^ |
|  |  |  |  |  |  |
| **cART regimen** |  |  |  |  |  |
| NNRTI or NRTI-based | 27 | 96.43% | 14 | 100.00% | 1.0000^+++^ |
| PI-based | 1 | 3.57% | 0 | 0.00% |  |
|  |  |  |  |  |  |
| **CD4+ (cells/μL)** |  |  |  |  |  |
| Mean+ SD | 27 | 418+140 | 14 | 485+187 | 0.2504^++^ |
|  |  |  |  |  |  |
| **HIV RNA (copies/mL)** |  |  |  |  |  |
| <40 (assay cut-off) | 26 | 92.86% | 14 | 100.00% | 0.5447^+++^ |
| ≥40 | 2 | 7.14% | 0 | 0.00% |  |
|  |  |  |  |  |  |
| **HIV DNA (log_10_ copies/10^6^ CD4^+^)** |  |  |  |  |  |
| Mean+ SD | 25 | 3.40+0.29 | 14 | 3.21+0.44 | 0.1658^++^ |

^++^ T Student Test; ^+++^ Fisher Exact Test; SD: Standard Deviation

**Supplementary Table 1b. Characteristics of females by treatment group at the ISS T-003 trial baseline**

|  | **n** | **Tat vaccine** | **n** | **Placebo** | **p value** |
| --- | --- | --- | --- | --- | --- |
|  | 58 |  | 61 |  |  |
| **Age** |  |  |  |  |  |
| Mean+ SD | 58 | 35+ 6 | 61 | 35+ 6 | 0.8251^++^ |
|  |  |  |  |  |  |
| **Years from HIV diagnosis** |  |  |  |  |  |
| Mean+ SD | 58 | 6+ 3 | 61 | 5+ 3 | 0.8398^++^ |
|  |  |  |  |  |  |
| **Years from cART initiation** |  |  |  |  |  |
| Mean+ SD | 58 | 4+ 2 | 61 | 5+ 3 | 0.9139^++^ |
|  |  |  |  |  |  |
| **cART regimen** |  |  |  |  |  |
| NNRTI or NRTI-based | 57 | 98.28% | 59 | 96.72% | 1.0000^+++^ |
| PI-based | 1 | 1.72% | 2 | 3.28% |  |
|  |  |  |  |  |  |
| **CD4+ (cells/μL)** |  |  |  |  |  |
| Mean+ SD | 58 | 572+249 | 61 | 577+169 | 0.8959^++^ |
|  |  |  |  |  |  |
| **HIV RNA (copies/mL)** |  |  |  |  |  |
| <40 (assay cut-off) | 55 | 96.49% | 61 | 100 00% | 0.2312^+++^ |
| ≥40 | 2 | 3.51% | 0 | 0 00% |  |
|  |  |  |  |  |  |
| **HIV DNA (log_10_ copies/10^6^ CD4^+^)** |  |  |  |  |  |
| Mean+ SD | 56 | 3.10+0.47 | 60 | 3.12+0.67 | 0.8938^++^ |
|  |  |  |  |  |  |

^++^ T Student Test; ^+++^ Fisher Exact Test; SD: Standard Deviation

**Supplementary Table 2. Median peak reciprocal titers for vaccinees and placebos**

|  | **Tat vaccinees** | | | | |  | **Placebos** | | | | |  |
| --- | --- | --- | --- | --- | --- | --- | --- | --- | --- | --- | --- | --- |
| **Anti-Tat Ab** | **N** | **Mean** | **Median** | **Minimum** | **Maximum** |  | **N** | **Mean** | **Median** | **Minimum** | **Maximum** | **p value** |
| **IgM** | 68 | 60 | 38 | 25 | 400 |  | 10 | 43 | 38 | 25 | 100 | 0.6341 |
| **IgG** | 82 | 1643 | 800 | 100 | 12800 |  | 11 | 227 | 100 | 100 | 800 | <0.0001 |
| **IgA** | 65 | 143 | 50 | 25 | 1600 |  | 6 | 46 | 38 | 25 | 100 | 0.2071 |

Median peak with minimum and maximum of reciprocal titers for vaccinees and placebos are shown. Statistical comparisons between the two groups were performed using the Mann-Whitney test.

**Supplementary Table 3: Between-arm differences and confidence intervals of CD4^+^ T cells and proviral DNA changes from baseline at each year and overall**

**A. All volunteers**

| **year** | **n Vaccinees** | **n Placebos** |
| --- | --- | --- |
| **1** | 86 | 75 |
| **3** | 86 | 75 |
| **4** | 55 | 52 |
| **11** | 54 | 47 |
| **12** | 22 | 15 |

| **CD4 changes from baseline, Differences between Vaccinees and Placebos by year, all volunteers** | | | | | |
| --- | --- | --- | --- | --- | --- |
| **year** | **Estimate difference vaccinees vs placebos** | **Standard Error** | **Adj P value** | **Adj Lower** | **Adj Upper** |
| 1 | 32.16 | 20.19 | 0.8522 | -31.73 | 96.05 |
| 3 | 12.93 | 25.36 | 1.0000 | -67.32 | 93.17 |
| 4 | 28.94 | 34.55 | 0.9980 | -80.36 | 138.23 |
| 11 | 44.66 | 45.90 | 0.9937 | -100.54 | 189.86 |
| 12 | 2.16 | 81.18 | 1.0000 | -254.67 | 258.00 |

| **Overall effect** | |  |  |  |
| --- | --- | --- | --- | --- |
| **Estimate difference vaccinees vs placebos** | **Standard Error** | **P value** | **Lower** | **Upper** |
| 24.17 | 28.02 | 0.3883 | -30.74 | 79.08 |

| **Proviral DNA changes from baseline, Differences between Vaccinees and Placebos by year, all volunteers** | | | | | |
| --- | --- | --- | --- | --- | --- |
| **year** | **Estimate difference vaccinees vs placebos** | **Standard Error** | **Adj P value** | **Adj Lower** | **Adj Upper** |
| 1 | **-0.1981** | 0.0666 | **0.0859** | -0.4087 | 0.0125 |
| 3 | -0.0524 | 0.0636 | 0.9982 | -0.2535 | 0.1488 |
| 4 | -0.0322 | 0.0931 | 1.0000 | -0.3266 | 0.2623 |
| 11 | -0.3183 | 0.2513 | 0.9610 | -1.1133 | 0.4767 |
| 12 | 0.1062 | 0.4111 | 1.0000 | -1.1942 | 1.4067 |

| **Overall effect** | |  |  |  |
| --- | --- | --- | --- | --- |
| **Estimate difference vaccinees vs placebos** | **Standard Error** | **P value** | **Lower** | **Upper** |
| -0.0990 | 0.1101 | 0.3689 | -0.3148 | 0.1169 |

**B. Females**

| **year** | **n Vaccinees** | **n Placebos** |
| --- | --- | --- |
| **1** | 58 | 61 |
| **3** | 58 | 61 |
| **4** | 36 | 43 |
| **11** | 38 | 37 |
| **12** | 14 | 12 |

| **CD4 changes from baseline, Differences between Vaccinees and Placebos by year, females** | | | | | |
| --- | --- | --- | --- | --- | --- |
| **year** | **Estimate difference vaccinees vs placebos** | **Standard Error** | **Adj P value** | **Adj Lower** | **Adj Upper** |
| 1 | 25.70 | 25.63 | 0.9922 | -55.39 | 106.79 |
| 3 | 2.44 | 31.98 | 1.0000 | -98.73 | 103.61 |
| 4 | 18.20 | 38.83 | 1.0000 | -104.63 | 141.03 |
| 11 | 44.53 | 55.88 | 0.9986 | -132.26 | 221.32 |
| 12 | -47.30 | 99.08 | 1.0000 | -360.75 | 266.16 |

| **Overall effect vaccinees vs placebos, females** | | | |  |
| --- | --- | --- | --- | --- |
| **Estimate difference vaccinees vs placebos** | **Standard Error** | **P value** | **Lower** | **Upper** |
| 24.17 | 8.71 | 0.7935 | -56.54 | 73.97 |

| **Proviral DNA changes from baseline, Differences between Vaccinees and Placebos by year, females** | | | | | |
| --- | --- | --- | --- | --- | --- |
| **year** | **Estimate difference vaccinees vs placebos** | **Standard Error** | **Adj P value** | **Adj Lower** | **Adj Upper** |
| 1 | -0.2000 | 0.0897 | 0.4361 | -0.4838 | 0.0838 |
| 3 | -0.0381 | 0.0739 | 1.0000 | -0.2718 | 0.1955 |
| 4 | -0.0251 | 0.0908 | 1.0000 | -0.3124 | 0.2623 |
| 11 | -0.4123 | 0.2985 | 0.9333 | -1.3566 | 0.5321 |
| 12 | 0.1634 | 0.5696 | 1.0000 | -1.6385 | 1.9653 |

| **Overall effect vaccinees vs placebos, females** | | | |  |
| --- | --- | --- | --- | --- |
| **Estimate difference vaccinees vs placebos** | **Standard Error** | **P value** | **Lower** | **Upper** |
| -0.1024 | 0.1363 | 0.4524 | -0.3695 | 0.1647 |

**C. Males**

| **year** | **n Vaccinees** | **n Placebos** |
| --- | --- | --- |
| **1** | 28 | 14 |
| **3** | 28 | 14 |
| **4** | 19 | 9 |
| **11** | 16 | 10 |
| **12** | 8 | 3 |

| **CD4 changes from baseline, Differences between Vaccinees and Placebos by year, males** | | | | | |
| --- | --- | --- | --- | --- | --- |
| **year** | **Estimate difference vaccinees vs placebos** | **Standard Error** | **Adj P value** | **Adj Lower** | **Adj Upper** |
| 1 | 75.09 | 38.76 | 0.6432 | -47.52 | 197.70 |
| 3 | 60.50 | 49.55 | 0.9692 | -96.26 | 217.26 |
| 4 | 92.77 | 73.59 | 0.9621 | -140.03 | 325.58 |
| 11 | 69.44 | 80.36 | 0.9974 | -184.81 | 323.68 |
| 12 | 132.56 | 128.48 | 0.9904 | -273.90 | 539.01 |

| **Overall effect vaccinees vs placebos, males** | | | |  |
| --- | --- | --- | --- | --- |
| **Estimate difference vaccinees vs placebos** | **Standard Error** | **P value** | **Lower** | **Upper** |
| 86.07 | 58.14 | 0.1388 | -27.88 | 200.02 |

| **Proviral DNA changes from baseline, Differences between Vaccinees and Placebos by year, males** | | | | | |
| --- | --- | --- | --- | --- | --- |
| **year** | **Estimate difference vaccinees vs placebos** | **Standard Error** | **Adj P value** | **Adj Lower** | **Adj Upper** |
| 1 | **-0.2455** | 0.0804 | **0.0692** | -0.4999 | 0.0089 |
| 3 | -0.1147 | 0.1120 | 0.9909 | -0.4691 | 0.2397 |
| 4 | -0.2866 | 0.2146 | 0.9455 | -0.9654 | 0.3922 |
| 11 | -0.1603 | 0.4114 | 1.0000 | -1.4618 | 1.1412 |
| 12 | -0.2462 | 0.2189 | 0.9823 | -0.9386 | 0.4462 |

| **Overall effect vaccinees vs placebos, males** | | | |  |
| --- | --- | --- | --- | --- |
| **Estimate difference vaccinees vs placebos** | **Standard Error** | **P value** | **Lower** | **Upper** |
| -0.2106 | 0.1495 | 0.1588 | -0.5036 | 0.0823 |

**D. Aviremics**

| **year** | **n Vaccinees** | **n Placebos** |
| --- | --- | --- |
| **1** | 58 | 45 |
| **3** | 58 | 45 |
| **4** | 35 | 32 |
| **11** | 38 | 30 |
| **12** | 13 | 11 |

| **CD4 changes from baseline, Differences between Vaccinees and Placebos by year, persistently aviremic** | | | | | |
| --- | --- | --- | --- | --- | --- |
| **year** | **Estimate difference vaccinees vs placebos** | **Standard Error** | **Adj P value** | **Adj Lower** | **Adj Upper** |
| 1 | 28.94 | 26.19 | 0.9844 | -53.92 | 111.80 |
| 3 | 26.78 | 28.89 | 0.9956 | -64.60 | 118.17 |
| 4 | 46.95 | 39.70 | 0.9751 | -78.63 | 172.54 |
| 11 | 37.76 | 56.41 | 0.9997 | -140.71 | 216.23 |
| 12 | -134.06 | 87.42 | 0.8790 | -410.64 | 142.52 |

| **Overall effect vaccinees vs placebos, persistently aviremic** | | | | |
| --- | --- | --- | --- | --- |
| **Estimate difference vaccinees vs placebos** | **Standard Error** | **P value** | **Lower** | **Upper** |
| 1.28 | 32.38 | 0.9686 | -62.18 | 64.73 |

| **Proviral DNA changes from baseline, Differences between Vaccinees and Placebos by year, persistently aviremic** | | | | | |
| --- | --- | --- | --- | --- | --- |
| **year** | **Estimate difference vaccinees vs placebos** | **Standard Error** | **Adj P value** | **Adj Lower** | **Adj Upper** |
| 1 | **-0.2624** | 0.0719 | **0.0098** | -0.4899 | -0.0350 |
| 3 | -0.0554 | 0.0507 | 0.9855 | -0.2158 | 0.1050 |
| 4 | -0.1239 | 0.0909 | 0.9384 | -0.4116 | 0.1637 |
| 11 | -0.3767 | 0.2767 | 0.9389 | -1.2522 | 0.4988 |
| 12 | -0.3390 | 0.5705 | 0.9999 | -2.1437 | 1.4658 |

| **Overall effect vaccinees vs placebos, persistently aviremic** | | | | |
| --- | --- | --- | --- | --- |
| **Estimate difference vaccinees vs placebos** | **Standard Error** | **P value** | **Lower** | **Upper** |
| **-0.2315** | 0.1304 | **0.0759** | -0.4871 | 0.0241 |

**E. Viremics**

| **year** | **n Vaccinees** | **n Placebos** |
| --- | --- | --- |
| **1** | 28 | 25 |
| **3** | 28 | 25 |
| **4** | 20 | 16 |
| **11** | 16 | 12 |
| **12** | 9 | 3 |

| **CD4 changes from baseline, Differences between Vaccinees and Placebos by year, with at least 1 episode of viral blip up to year 4** | | | | | |
| --- | --- | --- | --- | --- | --- |
| **year** | **Estimate difference vaccinees vs placebos** | **Standard Error** | **Adj P value** | **Adj Lower** | **Adj Upper** |
| 1 | 41.95 | 34.19 | 0.9682 | -66.21 | 150.12 |
| 3 | -22.19 | 51.24 | 1.0000 | -184.31 | 139.93 |
| 4 | 10.56 | 61.38 | 1.0000 | -183.62 | 204.75 |
| 11 | 82.53 | 95.22 | 0.9974 | -218.70 | 383.77 |
| 12 | 318.05 | 122.80 | 0.2221 | -70.43 | 706.53 |

| **Overall effect vaccinees vs placebos, with at least 1 episode of viral blip up to year 4** | | | | |
| --- | --- | --- | --- | --- |
| **Estimate difference vaccinees vs placebos** | **Standard Error** | **P value** | **Lower** | **Upper** |
| **86.18** | 46.75 | **0.0653** | -5.44 | 177.81 |

| **Proviral DNA changes from baseline, Differences between Vaccinees and Placebos by year, with at least 1 episode of viral blip up to year 4** | | | | | |
| --- | --- | --- | --- | --- | --- |
| **year** | **Estimate difference vaccinees vs placebos** | **Standard Error** | **Adj P value** | **Adj Lower** | **Adj Upper** |
| 1 | -0.0523 | 0.0680 | 0.9990 | -0.2673 | 0.1626 |
| 3 | 0.0504 | 0.0850 | 0.9999 | -0.2185 | 0.3193 |
| 4 | 0.0993 | 0.1952 | 1.0000 | -0.5183 | 0.7168 |
| 11 | -0.1396 | 0.5218 | 1.0000 | -1.7906 | 1.5113 |
| 12 | 0.2330 | 0.2328 | 0.9923 | -0.5036 | 0.9696 |

| **Overall effect vaccinees vs placebos, with at least 1 episode of viral blip up to year 4** | | | | |
| --- | --- | --- | --- | --- |
| **Estimate difference vaccinees vs placebos** | **Standard Error** | **P value** | **Lower** | **Upper** |
| 0.0381 | 0.1425 | 0.7889 | -0.2411 | 0.3174 |

Multiple pairwise comparisons of CD4^+^ T-cells and HIV Proviral DNA changes from baseline adjusted with the Tukey’s post hoc test by year and overall between vaccine and placebo groups in A. all volunteers, B. only Females, C. only Males, D. only aviremics and E. only vIremics are shown.

**Supplementary Table 4. Number of episodes of detectable viremia during the study for vaccinees and placebos.**

|  | **Tat vaccinees** | **Placebos** |
| --- | --- | --- |
|  | **n (%)** | **n (%)** |
| **VL always undetactable** | 58 (67%) | 45 (60%) |
| **only 1 VL rebound** | 11 (13%) | 15 (20%) |
| **2+ VL rebounds** | 17 (20%) | 15 (20%) |
| **Total** | 86 (100%) | 75 (100%) |

**Supplementary Figure 1. Anti-Tat Ab persistence in vaccinees and placebos up to year 12 of follow up, by Ig isotypes**

**
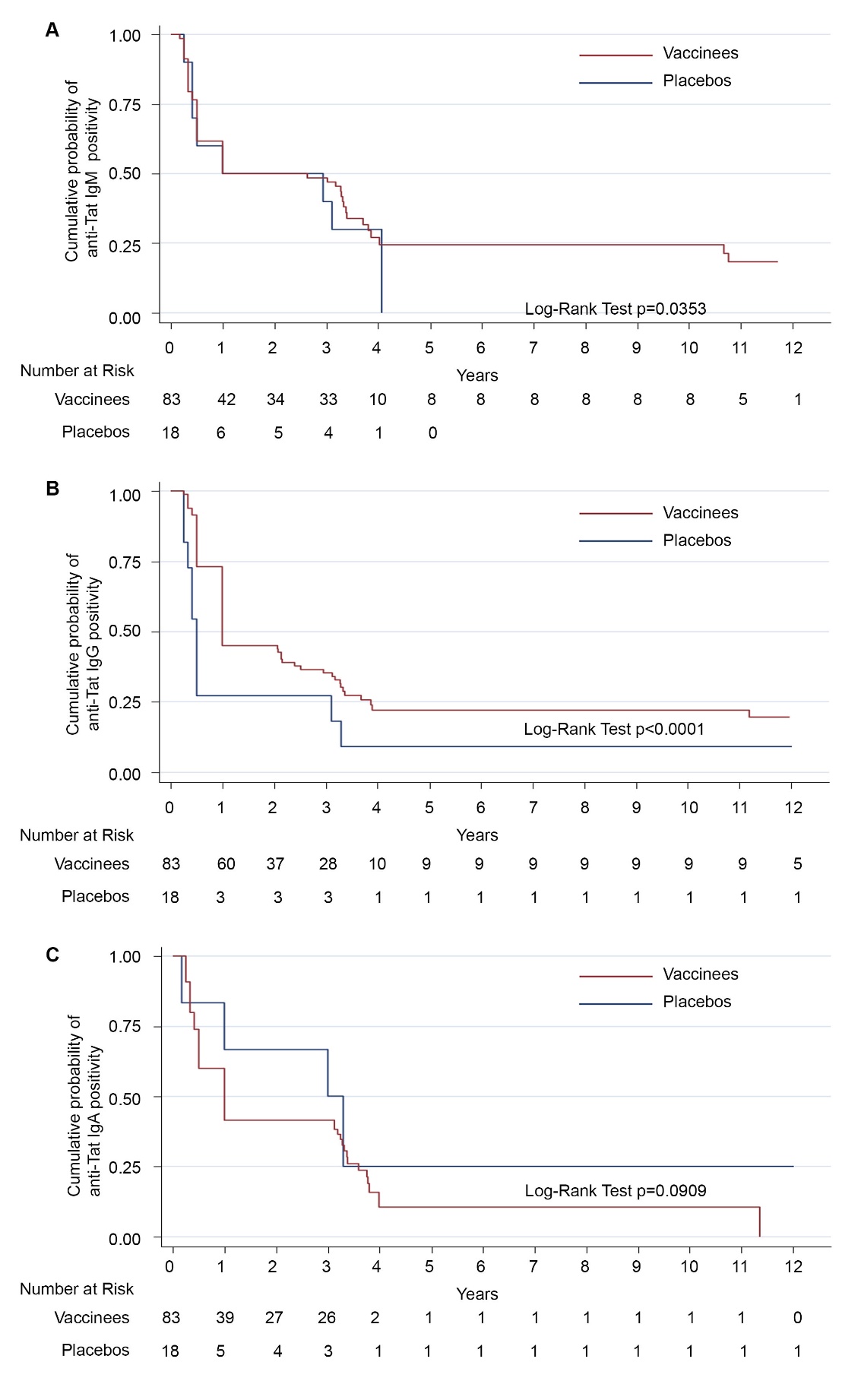
**

Kaplan-Meier estimates showing the cumulative probability of anti-Tat Ab durability in vaccinees and placebos up to 12 years of follow-up, by Ig isotypes: A. IgM, B. IgG, C. IgA. Log-Rank test indicated the statistically significant difference in durability between vaccinees and placebos for each Ig isotype.

**Supplementary Figure 2: Violin plots showing individual changes from baseline in CD4⁺ T-cell and HIV proviral DNA levels in vaccinees and placebos.**

**
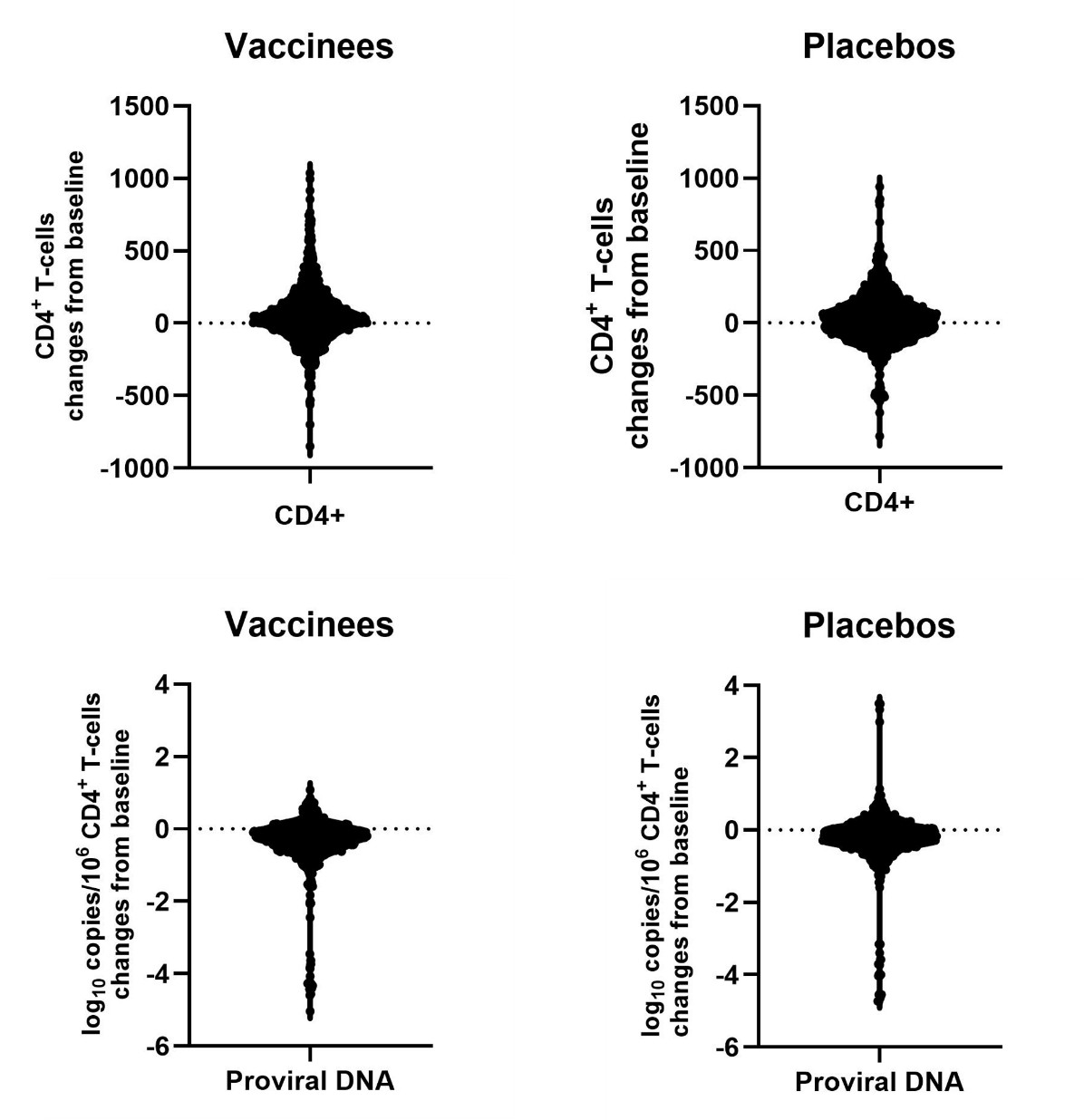
**

Violin plots showing the distribution of **individual changes from baseline in CD4⁺ T-cell and HIV proviral DNA level** in vaccinees and placebos. The width of each violin represents kernel density estimation. Overlaid individual data points represent single observations.
